# Supplementary material for: Has the introduction of direct oral anticoagulants (DOACs) in England increased emergency admissions for bleeding conditions? A longitudinal ecological study
Source: BMJ Open. 2020 May 30;10(5):e033357. doi: 10.1136/bmjopen-2019-033357 (PMC7264699; doi:10.1136/bmjopen-2019-033357)
Supplement: Supplementary data [file bmjopen-2019-033357supp001.pdf]

Appendix 1 BNF codes and ICD10 codes used to define exposure and outcome variables.

BNF codes used to define prescribing

0208020V - Warfarin

0208020X0 – Dabigatran

0208020X0 – edoxaban

0208020Y – rivaroxaban

0208020Z0 - Apixiban

ICD 10 Codes used to define outcomes

1. **Bleeding Complications**

ICD10 code	Name
M79.81	traumatic haematoma of the soft tissue
M25.00	Hemarthrosis, unspecified joint
K92	Other diseases of digestive system
K92.0	Haematemesis
K92.1	Melaena
K92.2	Gastrointestinal haemorrhage, unspecified
K62.5	Haemorrhage of anus and rectum
R58	Haemorrhage, not elsewhere classified
R04.0	Epistaxis
R04.1	Haemorrhage from throat
R04.2	Haemoptysis
R04.8	Haemorrhage from other sites in respiratory passages

R04.9	Haemorrhage from respiratory passages, unspecified
R31	Unspecified haematuria
N02	Recurrent or persistent haematuria
I60	Subarachnoid haemorrhage
I60.0	Subarachnoid haemorrhage from carotid siphon and bifurcation
I60.1	Subarachnoid haemorrhage from middle cerebral artery
I60.2	Subarachnoid haemorrhage from anterior communicating artery
I60.3	Subarachnoid haemorrhage from posterior communicating artery
I60.4	Subarachnoid haemorrhage from basilar artery
I60.5	Subarachnoid haemorrhage from vertebral artery
I60.6	Subarachnoid haemorrhage from other intracranial arteries
I60.7	Subarachnoid haemorrhage from intracranial artery, unspecified
I60.8	Other subarachnoid haemorrhage
I60.9	Subarachnoid haemorrhage, unspecified
I61	Intracerebral haemorrhage
I61.0	Intracerebral haemorrhage in hemisphere, subcortical
I61.1	Intracerebral haemorrhage in hemisphere, cortical
I61.2	Intracerebral haemorrhage in hemisphere, unspecified
I61.3	Intracerebral haemorrhage in brain stem
I61.4	Intracerebral haemorrhage in cerebellum
I61.5	Intracerebral haemorrhage, intraventricular
I61.6	Intracerebral haemorrhage, multiple localized
I61.8	Other intracerebral haemorrhage
I61.9	Intracerebral haemorrhage, unspecified
I62	Other nontraumatic intracranial haemorrhage
I62.0	Subdural haemorrhage (acute)(nontraumatic)
I62.1	Nontraumatic extradural haemorrhage

I62.9	Intracranial haemorrhage (nontraumatic), unspecified

2. Clotting Complications

**ICD10 code**	**Name**
I63	Cerebral infarction
I64	Stroke, not specified as haemorrhage or infarction
I26	Pulmonary embolism
I82	Other venous embolism and thrombosis
I80.0	Phlebitis and thrombophlebitis of superficial vessels of lower extremities
I80.1	Phlebitis and thrombophlebitis of femoral vein
I80.2	Deep vein thrombosis, Phlebitis and thrombophlebitis of other deep vessels of lower extremities
I80.3	Phlebitis and thrombophlebitis of lower extremities, unspecified
I80.8	Phlebitis and thrombophlebitis of other sites
I80.9	Phlebitis and thrombophlebitis of unspecified site
