# Supplementary figures and images for: Has the introduction of direct oral anticoagulants (DOACs) in England increased emergency admissions for bleeding conditions? A longitudinal ecological study
Source: BMJ Open. 2020 May 30;10(5):e033357. doi: 10.1136/bmjopen-2019-033357 (PMC7264699; doi:10.1136/bmjopen-2019-033357)

## Appendix 2 flow outlining inclusion/ exclusion and missing data

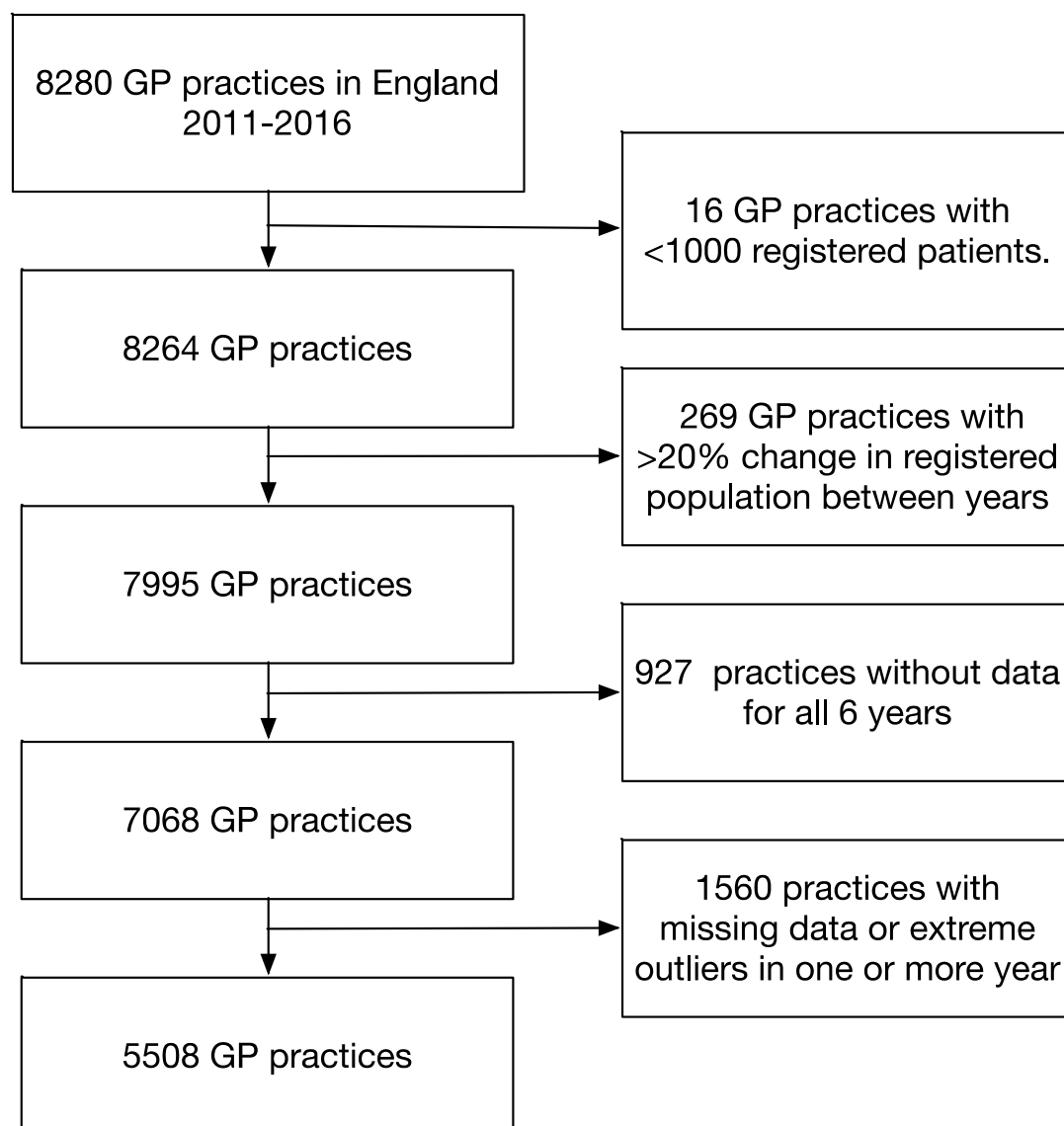

Supplement: Supplementary data [file bmjopen-2019-033357supp002.pdf]
