# Supplementary material for: Has the introduction of direct oral anticoagulants (DOACs) in England increased emergency admissions for bleeding conditions? A longitudinal ecological study
Source: BMJ Open. 2020 May 30;10(5):e033357. doi: 10.1136/bmjopen-2019-033357 (PMC7264699; doi:10.1136/bmjopen-2019-033357)

Appendix 3 alternative model specifications.

1. **Model using all GP practices – without applying exclusion criteria (8032 GP practices – 248 with missing data). Estimates from regression model showing the relative change in the rate of emergency admissions for bleeding and clotting complications associated with each additional 10% of DOACs prescribed as a proportion of all anticoagulants**

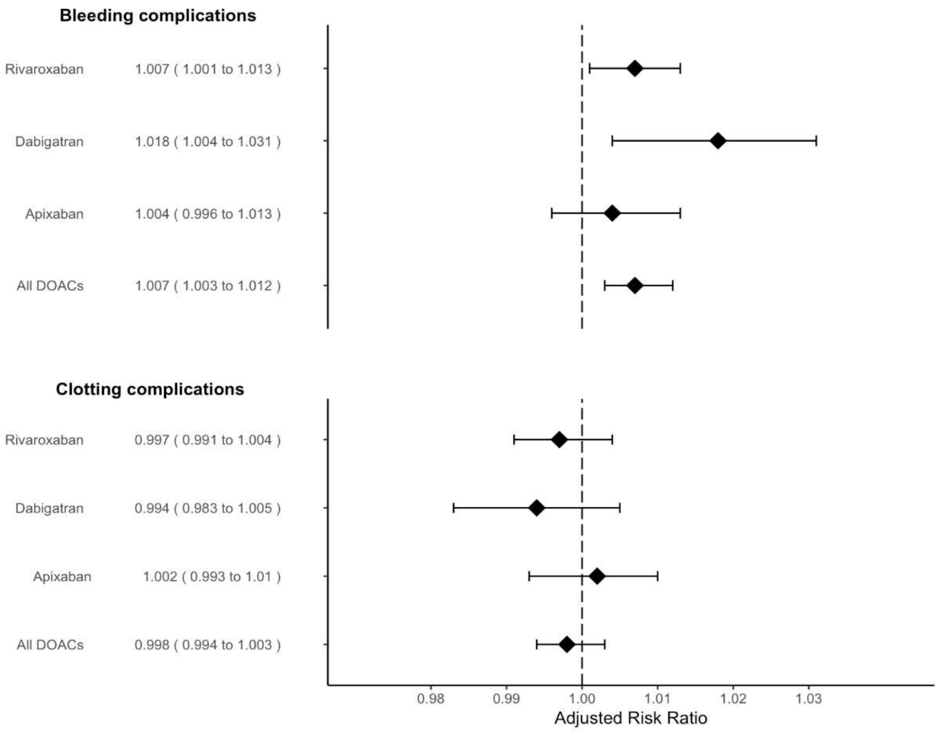

2. Negative Binomial Model

Estimates from regression model showing the relative change in the rate of emergency admissions for bleeding and clotting complications associated with each additional 10% of DOACs prescribed as a proportion of all anticoagulants

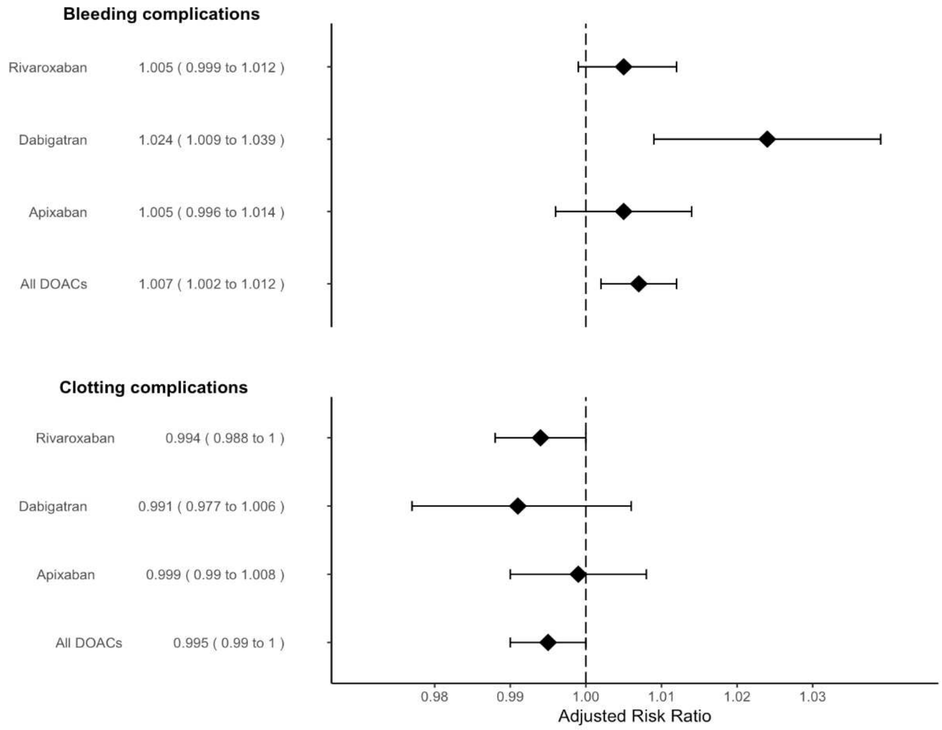

3. Non-equivalent dependent variable.

Estimates from regression model showing the relative change in the rate of emergency admissions for gastrointestinal admissions associated with each additional 10% of DOACs prescribed as a proportion of all anticoagulants.

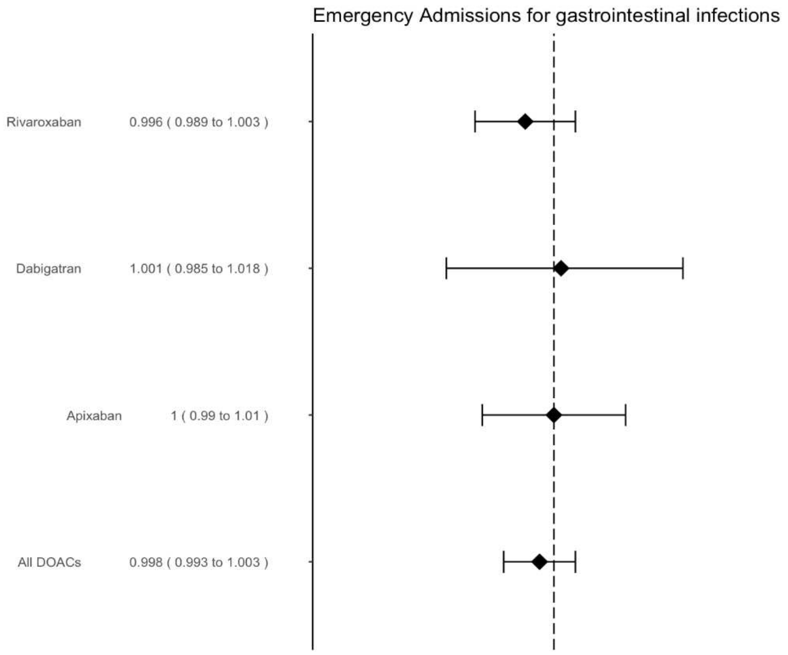

4. **Huber White Standard Errors**

Estimates from regression model showing the relative change in the rate of emergency admissions for bleeding and clotting complications associated with each additional 10% of DOACs prescribed as a proportion of all anticoagulants

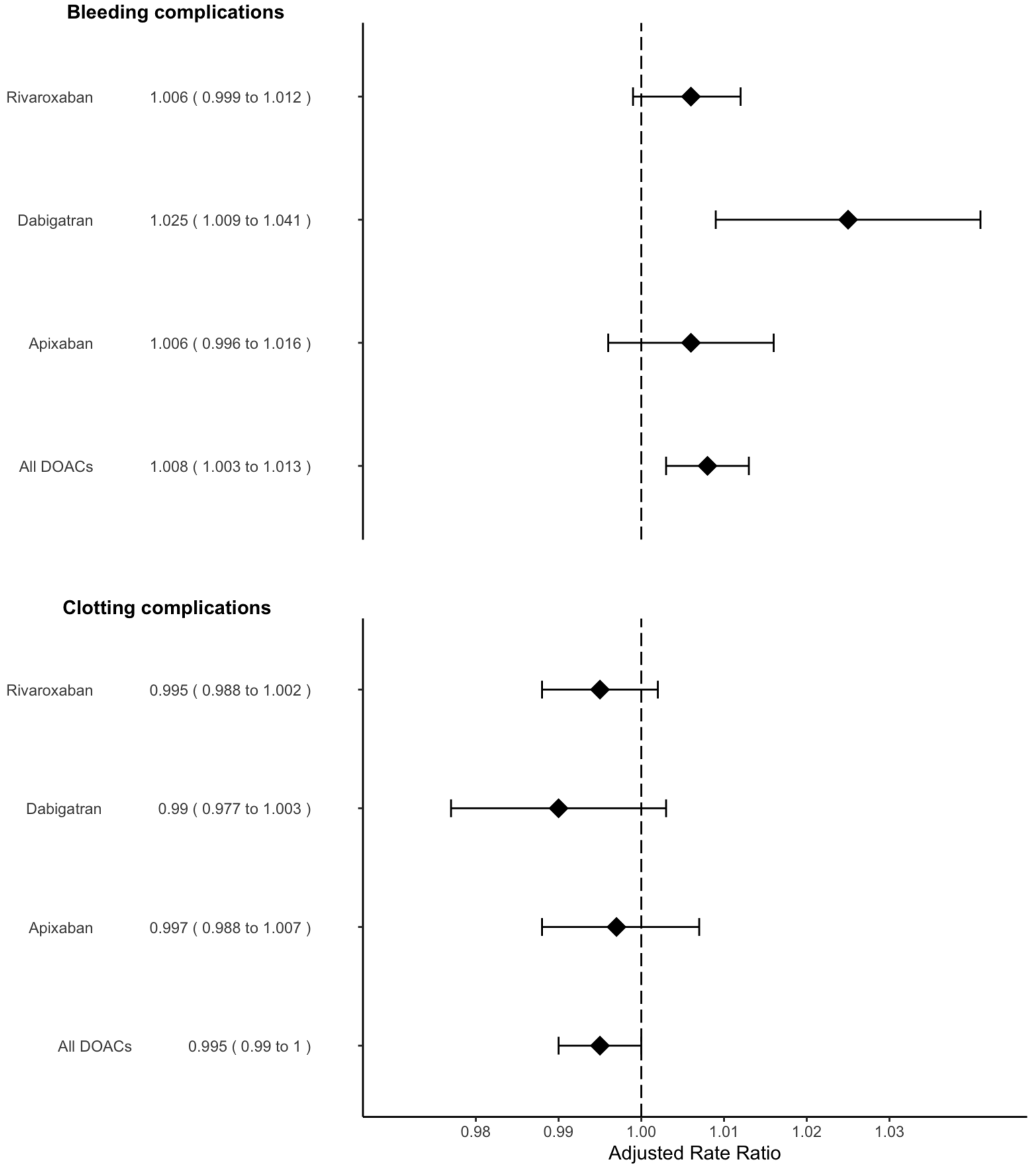

5. General Estimating equation - autoregressive(AR-1) correlation structure. Estimates from regression model showing the relative change in the rate of emergency admissions for bleeding and clotting complications associated with each additional 10% of DOACs prescribed as a proportion of all anticoagulants

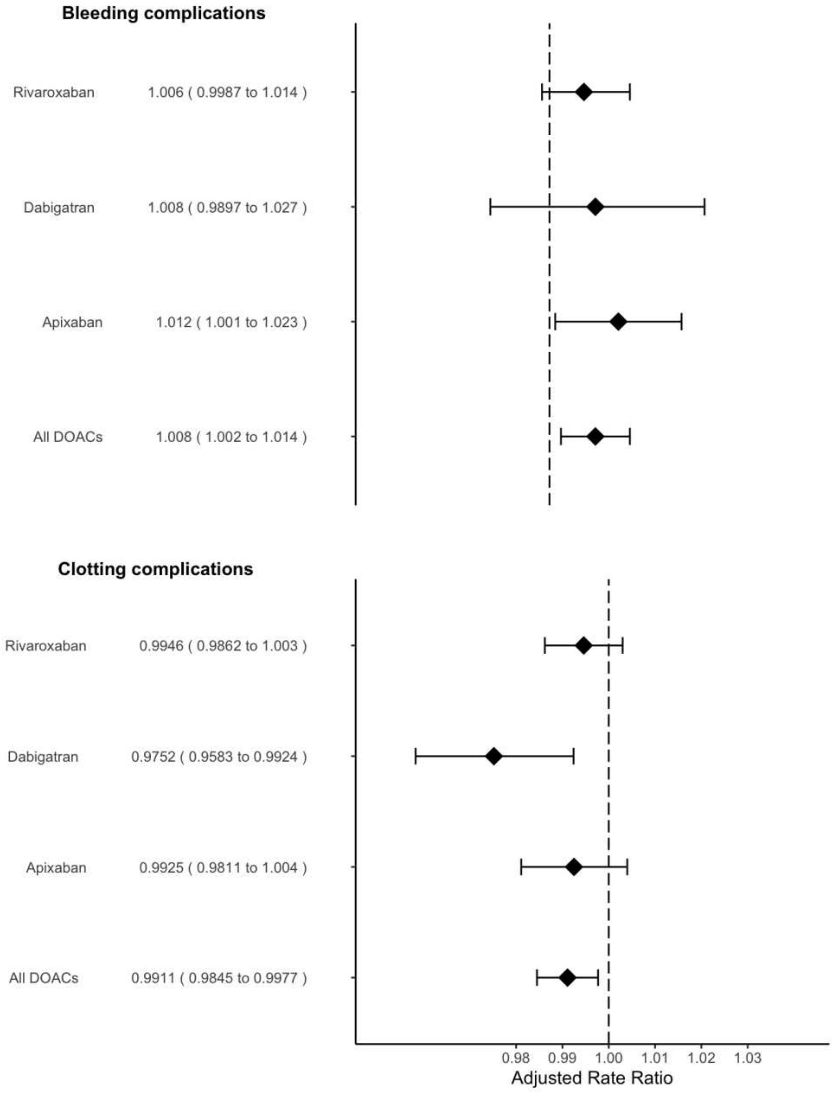

Supplement: Supplementary data [file bmjopen-2019-033357supp003.pdf]
