# Supplementary material for: Has the introduction of direct oral anticoagulants (DOACs) in England increased emergency admissions for bleeding conditions? A longitudinal ecological study
Source: BMJ Open. 2020 May 30;10(5):e033357. doi: 10.1136/bmjopen-2019-033357 (PMC7264699; doi:10.1136/bmjopen-2019-033357)
Supplement: Author's manuscript [file bmjopen-2019-033357.draft_revisions.pdf]

BMJ Open

BMJ Open is committed to open peer review. As part of this commitment we make the peer review history of every article we publish publicly available.

When an article is published we post the peer reviewers' comments and the authors' responses online. We also post the versions of the paper that were used during peer review. These are the versions that the peer review comments apply to.

The versions of the paper that follow are the versions that were submitted during the peer review process. They are not the versions of record or the final published versions. They should not be cited or distributed as the published version of this manuscript.

BMJ Open is an open access journal and the full, final, typeset and author-corrected version of record of the manuscript is available on our site with no access controls, subscription charges or pay-per-view fees (http://bmjopen.bmj.com).

If you have any questions on BMJ Open's open peer review process please email info.bmjopen@bmj.com

BMJ Open

Has the introduction of direct oral anticoagulants (DOACs) in England increased emergency admissions for bleeding conditions? A longitudinal ecological study.

Journal:	*BMJ Open*
Manuscript ID	bmjopen-2019-033357
Article Type:	Original research
Date Submitted by the Author:	01-Aug-2019
Complete List of Authors:	Alfirevic, Ana; University of Liverpool, Molecular and Clinical Pharmacology<br>Downing, Jennifer; University of Liverpool, Molecular and Clinical Pharmacology<br>Daras, Konstantinos ; University of Liverpool School of Environmental Sciences, Geography and Planning<br>Comerford, Terence; University of Liverpool<br>Pirmohamed, Munir; University of Liverpool, Molecular and Clinical Pharmacology<br>Barr, Ben
Keywords:	CLINICAL PHARMACOLOGY, PRIMARY CARE, PUBLIC HEALTH

SCHOLARONE™
Manuscripts

I, the Submitting Author has the right to grant and does grant on behalf of all authors of the Work (as defined in the below author licence), an exclusive licence and/or a non-exclusive licence for contributions from authors who are: i) UK Crown employees; ii) where BMJ has agreed a CC-BY licence shall apply, and/or iii) in accordance with the terms applicable for US Federal Government officers or employees acting as part of their official duties; on a worldwide, perpetual, irrevocable, royalty-free basis to BMJ Publishing Group Ltd ("BMJ") its licensees and where the relevant Journal is co-owned by BMJ to the co-owners of the Journal, to publish the Work in this journal and any other BMJ products and to exploit all rights, as set out in our licence.

The Submitting Author accepts and understands that any supply made under these terms is made by BMJ to the Submitting Author unless you are acting as an employee on behalf of your employer or a postgraduate student of an affiliated institution which is paying any applicable article publishing charge ("APC") for Open Access articles. Where the Submitting Author wishes to make the Work available on an Open Access basis (and intends to pay the relevant APC), the terms of reuse of such Open Access shall be governed by a Creative Commons licence – details of these licences and which Creative Commons licence will apply to this Work are set out in our licence referred to above.

Other than as permitted in any relevant BMJ Author's Self Archiving Policies, I confirm this Work has not been accepted for publication elsewhere, is not being considered for publication elsewhere and does not duplicate material already published. I confirm all authors consent to publication of this Work and authorise the granting of this licence.

For peer review only

Has the introduction of direct oral anticoagulants (DOACs) in England increased emergency admissions for bleeding conditions? A longitudinal ecological study.

Ana Alfirevic[1], Jennifer Downing[1], Konstantinos Daras[2], Terence Comerford[3], Munir Pirmohamed[1] and Ben Barr[2]

[1] The Wolfson Centre for Personalised Medicine, Department of Molecular and Clinical Pharmacology, Institute of Translational Medicine, University of Liverpool, UK

[2] Department of Public Health and Policy, Institute of Psychology, Health and Society, University of Liverpool, UK

[3] CLAHRC NWC Public Advisor Group, University of Liverpool, UK

Corresponding Author:

Ana Alfirevic

The Wolfson Centre for Personalised Medicine,

Department of Molecular and Clinical Pharmacology,

Institute of Translational Medicine

University of Liverpool

Block A, The Waterhouse Building

1-5 Brownlow Street

Liverpool, L69 3GL

Tel: +44 (0)151 7945551

E-mail: Ana.Alfirevic@liv.ac.uk

For peer review only

ABSTRACT

Objective. There is concern about long term safety of DOACs in clinical practice. Our aim was to investigate whether the introduction of DOACs compared with vitamin-K antagonists in England was associated with a change in admissions for bleeding or thromboembolic complications.

Setting: 5508 GP practices in England between 2011 and 2016.

Participants: All GP practices in England with a registered population size of greater than 1000 that had data for all 6 years.

Main outcome measure: The rate of emergency admissions to hospital for bleeding or thromboembolism, per 100,000 population for each GP practice in England.

Main exposure measure: The annual number of DOAC items prescribed for each GP practice population as a proportion of all anticoagulant items prescribed.

Design. This longitudinal ecological study used panel regression models to investigate the association between trends in DOAC prescribing within GP practice populations and trends in emergency admission rates for bleeding and thromboembolic conditions, whilst controlling for confounders.

Results. For each additional 10% of DOACs prescribed as a proportion of all anticoagulants, there was a 0.7% increase in bleeding complications (RR 1.007 95% CI 1.002 to 1.012). The introduction of DOACs between 2011 and 2016 was associated with additional 4929 (95%CI 2489 to 7370) emergency admissions for bleeding complications. Increased DOAC prescribing was associated with a slight decline in admission for thromboembolic conditions.

Conclusion. The rapid increase in prescribing of DOACs after changes in the NICE guidance in 2014 appears to have been associated with a higher rate of emergency admissions for bleeding conditions. These adverse consequences need to be considered in assessing the benefits and costs of the widespread use of DOACs.

Word count 271

Key words Direct oral anticoagulants, warfarin, emergency admissions, bleeding, thromboembolism

For peer review only

For peer review only - http://bmjopen.bmj.com/site/about/guidelines.xhtml

Article Summary

Strengths and limitations of this study

- The majority of GP practices in England were included in our analyses and therefore we could estimate effects across the whole population
- Longitudinal data analyses included the changes in the guidelines for prescribing anticoagulants
- We used effectiveness and safety data on anticoagulants used in England
- It was not possible to link information on the prescribing for specific individuals to particular hospital admissions due to the ecological study design
- No control for adherence to medications was possible

For peer review only

For peer review only - http://bmjopen.bmj.com/site/about/guidelines.xhtml

Introduction

Prescribing of direct oral anticoagulants (DOACs) such as dabigatran, rivaroxaban, apixaban and edoxaban that were licenced between 2013 and 2015, has increased rapidly in the UK. DOACs are used for stroke prevention in atrial fibrillation (AF), treatment of venous thromboembolism (VTE), and medical and surgical thromboprophylaxis.

Warfarin has been the primary oral anticoagulant in patients with AF and VTE for over 60 years. Its use is associated with large inter-individual variability in dose requirements, narrow therapeutic index, slow onset of action and concomitant drug and food interactions. In addition, regular International Normalised Ratio (INR) monitoring required for optimising loading and maintenance doses is burdensome to the patients and healthcare professionals. The DOACs are recommended by NICE guidelines for the treatment of adults with venous thromboembolism, prevention of recurrent deep vein thrombosis, and stroke prevention in patients with non-valvular atrial fibrillation[1]. Several non-inferiority randomised controlled trials have demonstrated that DOACs have comparable efficacy and safety profiles with warfarin [2-5]. This has led to widespread uptake of DOACs into clinical practice. Although DOACs have been shown to be cost-effective in RCTs[6], their high cost compared with the cost of warfarin (inclusive of INR testing) has major budget implications for the NHS in the UK and other healthcare services globally[7]. Concern about the escalating costs to healthcare is increasing not only for DOACs, but also when complications occur, for their reversal agents such as idarucizumab or andexanet alpha[8].

Data on the comparative safety and efficacy of DOACs in real-life observational studies has also become available more recently[6]. For instance, a recent large observational study in the UK found that apixaban was associated with decreased risk of bleeding complications, but rivaroxaban and low dose apixaban were associated with an increased risk of all-cause mortality compared to warfarin[1]. A large study (>14,500 patients) conducted in Scotland demonstrated that patients taking rivaroxaban may be at increased risk of bleeding compared with other DOACs[9]. Recent concern about bleeding

For peer review only - http://bmjopen.bmj.com/site/about/guidelines.xhtml

risks associated with DOACs has prompted the EMA to launch a safety review (https://www.ema.europa.eu/documents/other/direct-oral-anticoagulants-doacs-ema-starts-review-study-bleeding-risk-direct-oral-anticoagulants_en.pdf).

In this study we determined whether differences in prescribing of DOACs compared to warfarin between General Practitioner (GP) practices across England has been associated with variation of trends in emergency hospital admissions for bleeding and thromboembolic events from GP practice registered populations.

Methods

Setting and data sources.

We identified DOACs as dabigatran etexilate, apixaban, edoxaban or rivaroxaban using the BNF codes given in Appendix 1. We used the following NHS Digital data:

1) Practice level prescribing data that are published and made available by the NHS Business Service Authority (BSA) each month. We used the Practice Prescribing Data File with the full 15-digit BNF code to calculate the annual DOAC prescribing rate as the percentage of DOAC items prescribed in proportion to all anticoagulants items prescribed for each GP practice in England between 2011 and 2016. A prescription item refers to a single item prescribed on a prescription form, generally a course of medicine and is routinely used to measure trends in prescribing. We used the Net Ingredient Costs of these items included in this data set to calculate the prescribing costs.

2) Hospital Episode Statistics data were used to calculate the annual emergency admission rate for bleeding and clotting conditions for each GP practice. The ICD10 diagnostic codes included in this indicator is given in Appendix 1. Rates per 100,000 were calculated using annual data on the number of people registered with each GP practice provided by NHS Digital.

To additionally control for trends in the population age profile and underlying trends in morbidity, we also calculated the annual proportion of a GP population that was over 75 and used annual data on the

For peer review only - http://bmjopen.bmj.com/site/about/guidelines.xhtml

prevalence of Atrial Fibrillation (AF), Coronary Heart Disease (CHD) and Chronic Kidney Disease (CKD) for each GP practice population using data reported through the Quality and Outcome Framework (QOF) returns.

We included all GP practices in England with a registered population size of greater than 1000 that had data for all 6 years. We excluded GP practices with values for prevalence estimates from QOF that were clear outliers (more than 2 interquartile ranges (IQRs) below the first quartile or above the third quartile) as these probably represent reporting errors. In addition, we excluded practices where the practice registered population has changed by more than 20% between consecutive years as this would have reflected a large change in the practice population probable due to practice mergers or closures. This provided 5508 practices for the final analysis, each providing 6 years of data – i.e. 33,048 practice years of data. A flow chart is given in Appendix 2 detailing exclusions.

Analyses

Initially, we investigated geographical pattern of increases in DOACs prescribing across England. We mapped GP level prescribing data to Lower Super Output Areas (LSOA), based on the proportion of each GP practice's population that lived in each LSOA. Next, we plotted national maps for the DOAC prescribing rate each year. We then used a fixed effects Poisson regression model to investigate the association between the trend in prescribing within a GP practice and the trend in the rate of admissions for bleeding conditions. The outcome measure was the number of admissions and the log of the GP registered population was used as an offset. As there is potential confounding from unobserved factors that vary between GP practice populations, we used a fixed effects approach to remove these between-GP practice differences[27]. This conservative approach is the equivalent to including dummy variables for each GP practice so that the model assesses the association between the trend in prescribing and the trend in hospital admissions within each GP practice. We additionally included a dummy variable for each year to account for the national trend in prescribing and emergency admissions. To additionally control for differential trends in need for anticoagulants and risk of bleeding complications in each GP population, we included annual measures of the prevalence

For peer review only - http://bmjopen.bmj.com/site/about/guidelines.xhtml

of AF, CHD and CKD and the proportion of the GP population over 75-year olds. We used Huber-White clustered standard errors that are robust to divergence of the data from the assumptions of a Poisson distribution and the clustering of variance between GP practices. To investigate whether there were different effects associated with different types of DOACs, we repeated the analysis for dabigatran, apixaban and rivaroxoban. As edoxaban was only licenced in the middle of 2015, there was insufficient data to analyse this separately.

Sensitivity analysis

We subjected our analysis to a number of tests to assess the robustness of our findings. We estimated a negative binomial model as opposed to a Poisson model which is more robust to over-dispersion in the data and replicated our models using data from all GP practices (i.e. not applying any exclusion). To test the specificity of results, we estimated our model using a gastro-intestinal infection as a Nonequivalent Dependent Variables (NDV)[10]. This outcome should *not* be influenced by a change in the exposure but could be influenced by unobserved confounding factors that influence general trends in hospitalization in a GP practice population.

Patient involvement

No patients were involved in setting the research question or the outcome measures, nor were they involved in developing plans for design or implementation of the study. Patients and patient representatives within the NIHR CLAHRC NWC were asked to revise the manuscript in particular the lay language summary. The results of the study are going to be disseminated to the relevant patient and public groups through the CLAHRC NWC communication programme.

Results

Figure 1 shows the trend in the number of items and net drug costs of DOACs prescribed in England by GP practices between 2011 and 2016. Between 2011 and 2016, the number of items of anticoagulants prescribed had risen from 0.7 million to 1.4 million per month and the proportion of these that were DOACs had risen from <1% to 35%. Costs of anticoagulants rose more steeply, from

For peer review only - http://bmjopen.bmj.com/site/about/guidelines.xhtml

For peer review only

£1.6 million per month in 2011 to £26 million per month by the end of 2016. This rise in costs was due to increased prescribing of DOACs, which by the end of 2016 accounted for 94% of the cost of all anticoagulant prescribing.

Figure 2 shows the geographical pattern of DOAC prescribing each year. The increase in uptake of DOACs has not been consistent across the country, with the prescribing rate increasing markedly more in some part of the country compared to others. In particular, areas around Somerset, Kent, and to a lesser extent the North East, appeared to have adopted DOACs sooner than other areas. By 2016 there was markedly lower usage of DOACs in the North West and East Anglia.

The results of the regression model indicate that differences in the trends in DOAC prescribing between GP practices from 2011 to 2016 were associated with differences in the trends in admissions for bleeding conditions. For each additional 10% of DOACs prescribed as a proportion of all anticoagulants, there was a 0.7% increase in emergency admissions for bleeding complications (RR:1.007 95% CI 1.002 to 1.013). Increased risk of emergency admissions for bleeding complications was particularly associated with increased use of dabigatran. Increased use of DOACs was associated with a small decline in emergency admissions for clotting related conditions; however this was not statistically significant (p=0.065), see Figure 3.

Figure 4 shows the trend in emergency admissions for bleeding conditions between 2011 and 2016 and the trend, estimated from the regression model that would have been expected if DOACs had not been introduced. Overall the introduction of DOACs between 2011 and 2016 was associated with an additional 4929 emergency admissions for bleeding complications (95% CI 2489 to 7370).

Our results were similar when using alternative model specifications (see Appendix 3). We found similar results when using a negative binomial model as opposed to a Poisson regression and when replicating our model using data from all GP practices (i.e. not applying any exclusions). We found no association between the trend in DOAC prescribing and emergency admissions when applying the model with a non-equivalent dependant variable (gastro-intestinal infections).

For peer review only - http://bmjopen.bmj.com/site/about/guidelines.xhtml

Discussion

In this study, we demonstrated a sharp increase in the uptake of DOACs by general practitioners in the UK between 2011 and 2016, compared with the current standard, warfarin. The uptake of DOACs has not been consistent and there are geographical areas with markedly higher rate of DOAC prescribing. Worryingly, higher rates of DOAC prescribing were associated with higher rates of hospital admissions for bleeding events, with a slight decline in emergency admissions for thromboembolic complications. We projected that with every 10% increase of DOAC prescribing as a proportion of all anticoagulants, there was a 0.7% increase in emergency admissions for bleeding complications. Moreover, we estimated from our regression model that between 2011 and 2016, there were an additional 4929 emergency admissions for bleeding complications, more than would have been expected if DOACs had not been introduced. At the end of 2016, 94% of the total expenditure on anticoagulants prescribed by GP practices in England was spent on DOACs, which represented only 35% of all prescribed anticoagulants.

The strength of this study is that we analysed data over several years that included the changes in the guidelines for prescribing anticoagulants. In 2014, the UK National Institute for Health and Care Excellence (NICE) guidance recommended the use of DOACs and warfarin taking into consideration clinician and patient preferences. Comparable efficacy and the evidence from RCTs of better safety of DOACs compared with warfarin, combined with no need for regular monitoring and a wider therapeutic range, resulted in a major increase in prescribing[1 3 4 6]. We captured this increase in our longitudinal analyses. Our sample includes the majority of GP practices in England and is therefore able to estimate effects across the whole population. Our model assessed the association between the trend in prescribing and the trend in hospital admissions within each GP practice and we included a dummy variable for each year to account for the national trends. Any bias in our model of the association between our main exposure variable and outcome is therefore unlikely to be the result of time-invariant confounders that differ between GP practice population, or from any changes over time that effect all GP practices equally. We found a similar effect when using alternative models.

For peer review only - http://bmjopen.bmj.com/site/about/guidelines.xhtml

When replicating the analysis with a common emergency admission we would not expect to be affected by DOAC prescribing such as gastrointestinal infections, we found, as expected, no association. Additional strength of our study is that we included in our analyses both, effectiveness and safety data on anticoagulants used in England. We investigated whether increased bleeding risk has been compensated by a reduction in risk from clotting conditions and found that increased DOAC prescribing was associated with some reduction in emergency admission from thromboembolic conditions however, this effect was small and not statistically significant.

A number of limitations remain. Firstly, the ecological design meant that it was not possible to link information on the prescribing for specific individuals to particular hospital admissions. Therefore the associations observed at the aggregate level may not reflect associations at the level of the individual patients. Secondly, whilst our analysis adjusts for a number of observed and unobserved confounders, it is still possible that there are unobserved trends in factors that increase DOAC prescribing whilst also increasing risk of bleeding complications. Bias could be introduced for example if there are differential trends between GP practices in underlying risk of bleeding complications, that are not accounted for by our control variables and this increased risk of bleeding complications led to increased DOAC prescribing in these practices. As our outcomes were measured at the population level our study will, however, be less at risk of selection bias, than studies using individual follow up, where this results from clinical decisions to preferentially prescribe DOACs to patients with higher risk of bleeding complications. Thirdly, we could not control for adherence to medications in our study. Adherence to anticoagulants has been reviewed recently[11] with some contradictory reports. Although it is expected that adherence would be better for DOACs because of no need for regular monitoring, regular INR tests required to ensure the correct dose of warfarin lead to stringent check-ups for adherence, while DOACs monitoring is not available and therefore adherence declines after the initial period[12]. With long term adherence monitoring our estimates would have been more precise.

Comparisons with previous studies

For peer review only - http://bmjopen.bmj.com/site/about/guidelines.xhtml

Our results differ from the findings of several large-scale non-inferiority RCTs that have demonstrated better bleeding profiles of DOACs over warfarin[5 13-18]. This may not be surprising as trial data may provide limited information on relatively rare adverse effects due to low power and short follow up. In addition, participants in clinical trials may be younger with a fewer comorbidities. For example, renal impairment that occurs in the older population may be relevant. Renal clearance is more dominant for DOACs compared with warfarin, as 80%, 50%, 36% and 27% of unchanged dabigatran, edoxaban, rivaroxaban and apixaban respectively, are excreted in urine[19]. In addition, there is limited information available on the risk-benefit profile of DOACs in patients with severe renal function impairment[20-23] particularly in patients with diverse ethnic backgrounds[24].

Our findings also differ from the results of a recent retrospective cohort study in patients with intracranial haemorrhage who were previously prescribed warfarin or DOACs. Prior use of DOACs compared with prior use of warfarin was associated with lower risk of hospital mortality[25]. A recent network meta-analysis by Lopez-Lopez also showed that DOACs are safer than warfarin in relation to major and intracranial bleeding[6]. However interestingly, in that meta-analysis the risk of gastrointestinal bleeding was higher with dabigatran, edoxaban and rivaroxaban than with warfarin. In addition, edoxaban (30mg and 60 mg twice daily) significantly increased the risk of clinically relevant bleeding compared with warfarin[6]. Although our study uses a longitudinal ecological study design, at least in part, our results are comparable with previous studies. In a large (>59,000 participants) population-based observational study conducted in Canada and the US, the risk of hospital admissions for major bleeding or all-cause mortality in the first 90 days of treatment was similar for DOACs and warfarin [26].

It has been estimated that the cost of DOACs will rise sharply before patent expiry (in 2022) and by the year 2020 will constitute approximately 5% of the total NHS drug budget[12]. The drugs budget is the NHS's second biggest cost after its staff[27]. Cost of warfarin including INR monitoring for one patient per annum has been estimated to be £220 (https://www.gwh.nhs.uk/media/236108/doacs-for-dvt-pe-august-2016-v-9.pdf), while the cost of rivaroxaban (15mg bd for 3/52 loading (provided by

hospital), then 20mg o.d. has been estimated to £657 p.a. (https://www.gwh.nhs.uk/media/236108/doacs-for-dvt-pe-august-2016-v-9.pdf). Similar costs have been estimated for other DOACs. Additionally, there is a huge discrepancy in price of agents that are used to reverse the anticoagulant effects of warfarin (antidote vitamin K at £0.38) or DOACs (idarucizumab, reversal agent for dabigatran at £2,400 for a single treatment course of 2X 2.5 g infusions (https://www.nice.org.uk/advice/esnm73) or andexanet alpha, reversal agent for factor Xa inhibitors approved by the FDA in May 2018, at approximately £1,500). These high costs of DOACs could be avoided if warfarin treatment is optimised, for example through novel methods such as genotype-guided dosing[28 29] and point-of-care INR monitoring[30], and only individuals who are at higher risk of developing bleeding events, for example those with variant alleles that increase the risk of bleeding from warfarin[31], could be prescribed DOACs. Genotype-guided dosing of warfarin has been shown to be cost-effective[32]. It has been shown previously that approximately 55% of variability in warfarin dose requirements can be estimated from clinical and genetic data of three polymorphisms in the warfarin molecular target (vitamin K epoxide reductase gene) and metabolising enzyme (CYP2C9 gene) and genotype-guided dosing can reduce the risk of major bleeding[33 34].

Conclusions

This study is one of a few studies in the UK[1 35 36] that have evaluated the association between the rise in prescribing of DOACs after changes in NICE guidance in 2014 and emergency hospital admissions for bleeding events. We found that the rate of emergency admissions for bleeding conditions increased to a greater extent in GP practices that were more likely to prescribe DOACs compared with warfarin. With rapidly increasing use of DOACs, these potential adverse consequences need to be taken into account when assessing the benefits and costs of anticoagulant treatment in clinical practice. It is not clear whether the DOACs are being prescribed without adequate notice being taken of restrictions and warnings in the summary of product characteristics, and in addition, whether closer monitoring is required in certain patient groups to increase the safety of use in clinical practice. The recently announced review by the EMA is thus important in order to

For peer review only - http://bmjopen.bmj.com/site/about/guidelines.xhtml

further optimise the use of all oral anticoagulants so that risks are minimised while at the same time maximising benefits.

PPI statement

The study was supported by a patient advisory group, which provided input to the NIHR- CLAHRC NWC programme of research. This patient advisory group met on a regular basis for the duration of the study. Due to data protection restrictions and the very technical methods required to do our data analyses they were not involved in the analysis part of the project. PPI representatives will however, write a plain language summary and design a leaflet for dissemination to their peers and distributing to patient groups.

Author contributions

Ana Alfirevic- Substantial contributions to the conception and design of the work, drafting a manuscript. Agreed to be accountable for all aspects of the work in ensuring that questions related to the accuracy or integrity of any part of the work are appropriately investigated and resolved.

Ben Barr- Substantial contribution to the design of the work, the acquisition, analysis and interpretation of data. Agreed to be accountable for all aspects of the work in ensuring that questions related to the accuracy or integrity of any part of the work are appropriately investigated and resolved.

Munir Pirmohamed- Substantial contribution to the interpretation of the results, critical revision of the manuscript for important intellectual content and final approval of the manuscript.

Jennifer Downing- Critical revision of the manuscript for important intellectual content and final approval of the manuscript.

Konstantinos Daras- Substantial contribution to data acquisition, data analysis and interpretation of data for the work. Critical revision of the manuscript for important intellectual content and final approval of the manuscript

Terence Comerford- Critical revision of the manuscript for important intellectual content and final approval of the manuscript. Preparation of plain language summary for dissemination of findings.

Patient consent for publication: *Not required.*
Funding: *This work was supported by the National Institute for Health Research, Collaboration for Leadership and Health Research and Care North West Coast (NIHR CLAHRC NWC) and the state Department of Health and Social Care.*
Disclaimer: *The views expressed are those of the author(s) and not necessarily those of the NHS, the NIHR or the Department of Health.*
Competing interests: *We have read and understood BMJ policy on declaration of interests and declare the following: All authors have completed the ICMJE uniform disclosure form at http://www.icmje.org/coi_disclosure.pdf and declare: no support from any organisation for the submitted work [or describe if any]; no financial relationships with any organisations that might have an interest in the submitted work in the previous three years [or describe if any], no other*

For peer review only - http://bmjopen.bmj.com/site/about/guidelines.xhtml

relationships or activities that could appear to have influenced the submitted work [or describe if any].

Provenance and peer review: *Not commissioned; externally peer reviewed.*
Data sharing statement: *The dataset from this study will be made available on request to benbarr@liverpool.ac.uk*

Summary box

What is already known on this topic?

Direct oral anticoagulants (DOACs) are used for stroke prevention in atrial fibrillation, treatment of venous thromboembolism, and medical and surgical thromboprophylaxis.

Prescribing of DOACs has increased rapidly in the UK after changes in the NICE guidance in 2014, however, there is concern about their long term safety in clinical practice.

What this study adds?

This study shows that the rate of emergency admissions for bleeding conditions increased to a greater extent in GP practices in England that were more likely to prescribe DOACs compared with warfarin.

With rapidly increasing use of DOACs, these potential adverse consequences need to be taken into account when assessing the benefits and costs of anticoagulant treatment in clinical practice.

For peer review only - http://bmjopen.bmj.com/site/about/guidelines.xhtml

References

1. Vinogradova Y, Coupland C, Hill T, et al. Risks and benefits of direct oral anticoagulants versus warfarin in a real world setting: cohort study in primary care. BMJ (Clinical research ed 2018;**362**:k2505 doi: 10.1136/bmj.k2505[published Online First: Epub Date]|.
2. Agnelli G, Buller HR, Cohen A, et al. Oral apixaban for the treatment of acute venous thromboembolism. The New England journal of medicine 2013;**369**(9):799-808 doi: 10.1056/NEJMoa1302507[published Online First: Epub Date]|.
3. Investigators E, Bauersachs R, Berkowitz SD, et al. Oral rivaroxaban for symptomatic venous thromboembolism. The New England journal of medicine 2010;**363**(26):2499-510 doi: 10.1056/NEJMoa1007903[published Online First: Epub Date]|.
4. Investigators E-P, Buller HR, Prins MH, et al. Oral rivaroxaban for the treatment of symptomatic pulmonary embolism. The New England journal of medicine 2012;**366**(14):1287-97 doi: 10.1056/NEJMoa1113572[published Online First: Epub Date]|.
5. Schulman S, Kearon C, Kakkar AK, et al. Dabigatran versus warfarin in the treatment of acute venous thromboembolism. The New England journal of medicine 2009;**361**(24):2342-52 doi: 10.1056/NEJMoa0906598[published Online First: Epub Date]|.
6. Lopez-Lopez JA, Sterne JAC, Thom HHZ, et al. Oral anticoagulants for prevention of stroke in atrial fibrillation: systematic review, network meta-analysis, and cost effectiveness analysis. BMJ (Clinical research ed 2017;**359**:j5058 doi: 10.1136/bmj.j5058[published Online First: Epub Date]|.
7. Protty MB, Hayes J. Dawn of the direct-acting oral anticoagulants: trends in oral anticoagulant prescribing in Wales 2009-2015. Journal of clinical pharmacy and therapeutics 2017;**42**(2):132-34 doi: 10.1111/jcpt.12481[published Online First: Epub Date]|.
8. Burnett A, Siegal D, Crowther M. Specific antidotes for bleeding associated with direct oral anticoagulants. BMJ (Clinical research ed 2017;**357**:j2216 doi: 10.1136/bmj.j2216[published Online First: Epub Date]|.
9. Mueller T, Alvarez-Madrazo S, Robertson C, et al. Comparative safety and effectiveness of direct oral anticoagulants in patients with atrial fibrillation in clinical practice in Scotland. British journal of clinical pharmacology 2019;**85**(2):422-31 doi: 10.1111/bcp.13814[published Online First: Epub Date]|.
10. Shadish WR, Cook TD, Thomas. CD. *Experimental and Quasi-Experimental Designs for Generalised Causal Inference*: Houghton Mifflin, 2002.
11. Obamiro KO, Chalmers L, Bereznicki LR. A Summary of the Literature Evaluating Adherence and Persistence with Oral Anticoagulants in Atrial Fibrillation. American journal of cardiovascular drugs : drugs, devices, and other interventions 2016;**16**(5):349-63 doi: 10.1007/s40256-016-0171-6[published Online First: Epub Date]|.
12. Burn J, Pirmohamed M. Direct oral anticoagulants versus warfarin: is new always better than the old? Open heart 2018;**5**(1):e000712 doi: 10.1136/openhrt-2017-000712[published Online First: Epub Date]|.
13. Connolly SJ, Ezekowitz MD, Yusuf S, et al. Dabigatran versus warfarin in patients with atrial fibrillation. The New England journal of medicine 2009;**361**(12):1139-51 doi: 10.1056/NEJMoa0905561[published Online First: Epub Date]|.
14. Ezekowitz MD, Connolly S, Parekh A, et al. Rationale and design of RE-LY: randomized evaluation of long-term anticoagulant therapy, warfarin, compared with dabigatran.

For peer review only - http://bmjopen.bmj.com/site/about/guidelines.xhtml

American heart journal 2009;**157**(5):805-10, 10 e1-2 doi: 10.1016/j.ahj.2009.02.005[published Online First: Epub Date]|.
15. Ogawa S, Shinohara Y, Kanmuri K. Safety and efficacy of the oral direct factor xa inhibitor apixaban in Japanese patients with non-valvular atrial fibrillation. -The ARISTOTLE-J study. Circulation journal : official journal of the Japanese Circulation Society 2011;**75**(8):1852-9
16. Patel MR, Mahaffey KW, Garg J, et al. Rivaroxaban versus warfarin in nonvalvular atrial fibrillation. The New England journal of medicine 2011;**365**(10):883-91 doi: 10.1056/NEJMoa1009638[published Online First: Epub Date]|.
17. Ruff CT, Giugliano RP, Antman EM, et al. Evaluation of the novel factor Xa inhibitor edoxaban compared with warfarin in patients with atrial fibrillation: design and rationale for the Effective aNticoaGulation with factor xA next GEneration in Atrial Fibrillation-Thrombolysis In Myocardial Infarction study 48 (ENGAGE AF-TIMI 48). American heart journal 2010;**160**(4):635-41 doi: 10.1016/j.ahj.2010.06.042[published Online First: Epub Date]|.
18. Weitz JI, Connolly SJ, Patel I, et al. Randomised, parallel-group, multicentre, multinational phase 2 study comparing edoxaban, an oral factor Xa inhibitor, with warfarin for stroke prevention in patients with atrial fibrillation. Thromb Haemost 2010;**104**(3):633-41 doi: 10.1160/TH10-01-0066[published Online First: Epub Date]|.
19. Lutz J, Jurk K, Schinzel H. Direct oral anticoagulants in patients with chronic kidney disease: patient selection and special considerations. International journal of nephrology and renovascular disease 2017;**10**:135-43 doi: 10.2147/IJNRD.S105771[published Online First: Epub Date]|.
20. Buckley LF, Rybak E, Aldemerdash A, et al. Direct oral anticoagulants in patients with atrial fibrillation and renal impairment, extremes in weight, or advanced age. Clinical cardiology 2017;**40**(1):46-52 doi: 10.1002/clc.22591[published Online First: Epub Date]|.
21. Fanikos J, Burnett AE, Mahan CE, et al. Renal Function and Direct Oral Anticoagulant Treatment for Venous Thromboembolism. The American journal of medicine 2017;**130**(10):1137-43 doi: 10.1016/j.amjmed.2017.06.004[published Online First: Epub Date]|.
22. Fanikos J, Burnett AE, Mahan CE, et al. Renal Function Considerations for Stroke Prevention in Atrial Fibrillation. The American journal of medicine 2017;**130**(9):1015-23 doi: 10.1016/j.amjmed.2017.04.015[published Online First: Epub Date]|.
23. Zou R, Tao J, Shi W, et al. Meta-analysis of safety and efficacy for direct oral anticoagulation treatment of non-valvular atrial fibrillation in relation to renal function. Thrombosis research 2017;**160**:41-50 doi: 10.1016/j.thromres.2017.10.013[published Online First: Epub Date]|.
24. Wang KL, Chiu CC, Giugliano RP, et al. Drug Class, Renal Elimination, and Outcomes of Direct Oral Anticoagulants in Asian Patients: A Meta-Analysis. J Stroke Cerebrovasc Dis 2018;**27**(4):857-64 doi: 10.1016/j.jstrokecerebrovasdis.2017.10.027[published Online First: Epub Date]|.
25. Inohara T, Xian Y, Liang L, et al. Association of Intracerebral Hemorrhage Among Patients Taking Non-Vitamin K Antagonist vs Vitamin K Antagonist Oral Anticoagulants With In-Hospital Mortality. JAMA 2018;**319**(5):463-73 doi: 10.1001/jama.2017.21917[published Online First: Epub Date]|.
26. Jun M, Lix LM, Durand M, et al. Comparative safety of direct oral anticoagulants and warfarin in venous thromboembolism: multicentre, population based, observational

For peer review only

For peer review only - http://bmjopen.bmj.com/site/about/guidelines.xhtml

study. BMJ (Clinical research ed 2017;**359**:j4323 doi: 10.1136/bmj.j4323[published Online First: Epub Date]|.
27. The L. Personalised medicine in the UK. Lancet 2018;**391**(10115):e1 doi: 10.1016/S0140-6736(17)33261-0[published Online First: Epub Date]|.
28. Yang T, Zhou Y, Chen C, et al. Genotype-guided dosing versus conventional dosing of warfarin: A meta-analysis of 15 randomized controlled trials. Journal of clinical pharmacy and therapeutics 2018 doi: 10.1111/jcpt.12782[published Online First: Epub Date]|.
29. Jorgensen AL, Prince C, Fitzgerald G, et al. Implementation of genotype-guided dosing of warfarin with point-of-care genetic testing in three UK clinics: a matched cohort study. BMC Med 2019;**17**(1):76 doi: 10.1186/s12916-019-1308-7[published Online First: Epub Date]|.
30. Wool GD. Benefits and Pitfalls of Point-of-Care Coagulation Testing for Anticoagulation Management: An ACLPS Critical Review. American journal of clinical pathology 2019;**151**(1):1-17 doi: 10.1093/ajcp/aqy087[published Online First: Epub Date]|.
31. Kawai VK, Cunningham A, Vear SI, et al. Genotype and risk of major bleeding during warfarin treatment. Pharmacogenomics 2014;**15**(16):1973-83 doi: 10.2217/pgs.14.153[published Online First: Epub Date]|.
32. Pink J, Pirmohamed M, Lane S, et al. Cost-effectiveness of pharmacogenetics-guided warfarin therapy vs. alternative anticoagulation in atrial fibrillation. Clin Pharmacol Ther 2014;**95**(2):199-207 doi: 10.1038/clpt.2013.190[published Online First: Epub Date]|.
33. Gage BF, Bass AR, Lin H, et al. Effect of Genotype-Guided Warfarin Dosing on Clinical Events and Anticoagulation Control Among Patients Undergoing Hip or Knee Arthroplasty: The GIFT Randomized Clinical Trial. JAMA 2017;**318**(12):1115-24 doi: 10.1001/jama.2017.11469[published Online First: Epub Date]|.
34. Pirmohamed M, Burnside G, Eriksson N, et al. A randomized trial of genotype-guided dosing of warfarin. The New England journal of medicine 2013;**369**(24):2294-303 doi: 10.1056/NEJMoa1311386[published Online First: Epub Date]|.
35. Green L, Tan J, Antoniou S, et al. Haematological management of major bleeding associated with direct oral anticoagulants - UK experience. Br J Haematol 2019 doi: 10.1111/bjh.15808[published Online First: Epub Date]|.
36. Green L, Tan J, Morris JK, et al. A three-year prospective study of the presentation and clinical outcomes of major bleeding episodes associated with oral anticoagulant use in the UK (ORANGE study). Haematologica 2018;**103**(4):738-45 doi: 10.3324/haematol.2017.182220[published Online First: Epub Date]|.

For peer review only

For peer review only - http://bmjopen.bmj.com/site/about/guidelines.xhtml

Figure legends

Figure 1. Trend in anticoagulant prescribing items and costs from GP practices in England and the proportion of direct oral anticoagulants of all anticoagulants prescribed

Figure 2. Geographical pattern in DOAC prescribing as a proportion of all anticoagulant prescribing items in England 2011-2016.

Figure 3 Estimates from regression model showing the relative change in the rate of emergency admissions for bleeding and clotting complications associated with each additional 10% of DOACs prescribed as a proportion of all anticoagulants

Figure 4. Trend in emergency admissions for bleeding conditions between 2011 and 2016 and the trend, estimated from the regression model that would have been expected if DOACs had not been introduced.

For peer review only

For peer review only - http://bmjopen.bmj.com/site/about/guidelines.xhtml

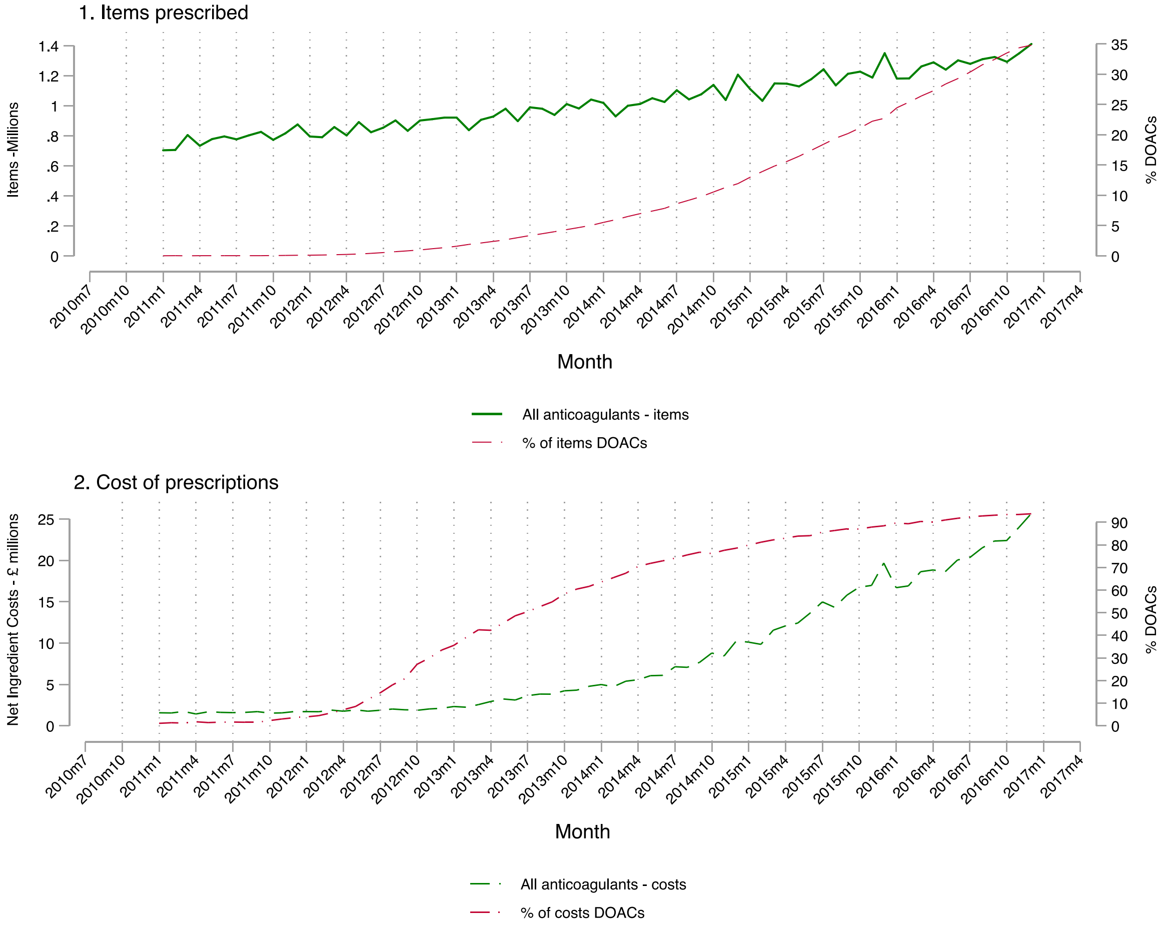
1. Items prescribed
Items -Millions
% DOACs
Month
All anticoagulants - items
% of items DOACs
2. Cost of prescriptions
Net Ingredient Costs - £ millions
% DOACs
Month
All anticoagulants - costs
% of costs DOACs

ew only

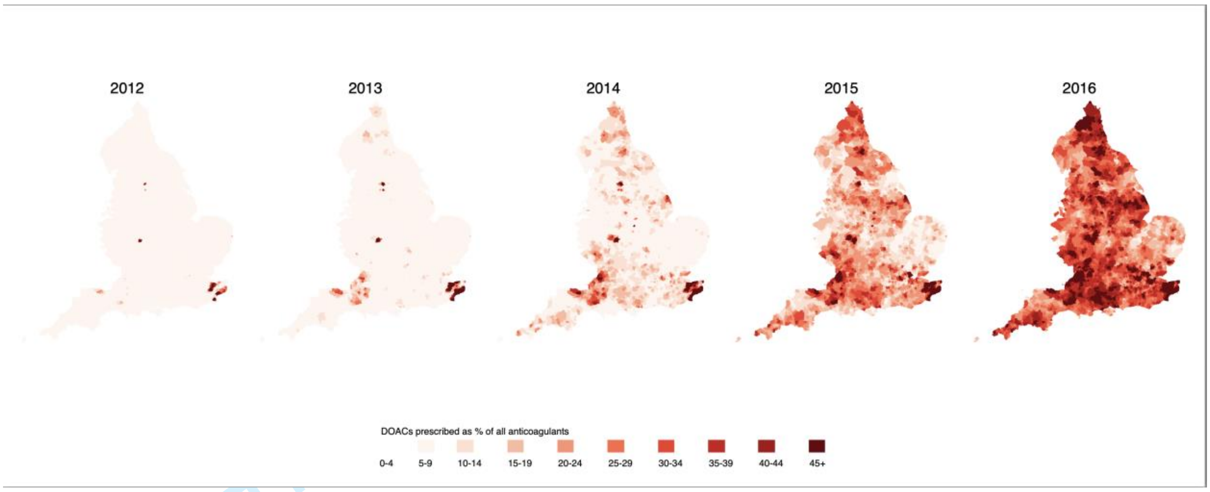

For peer review only

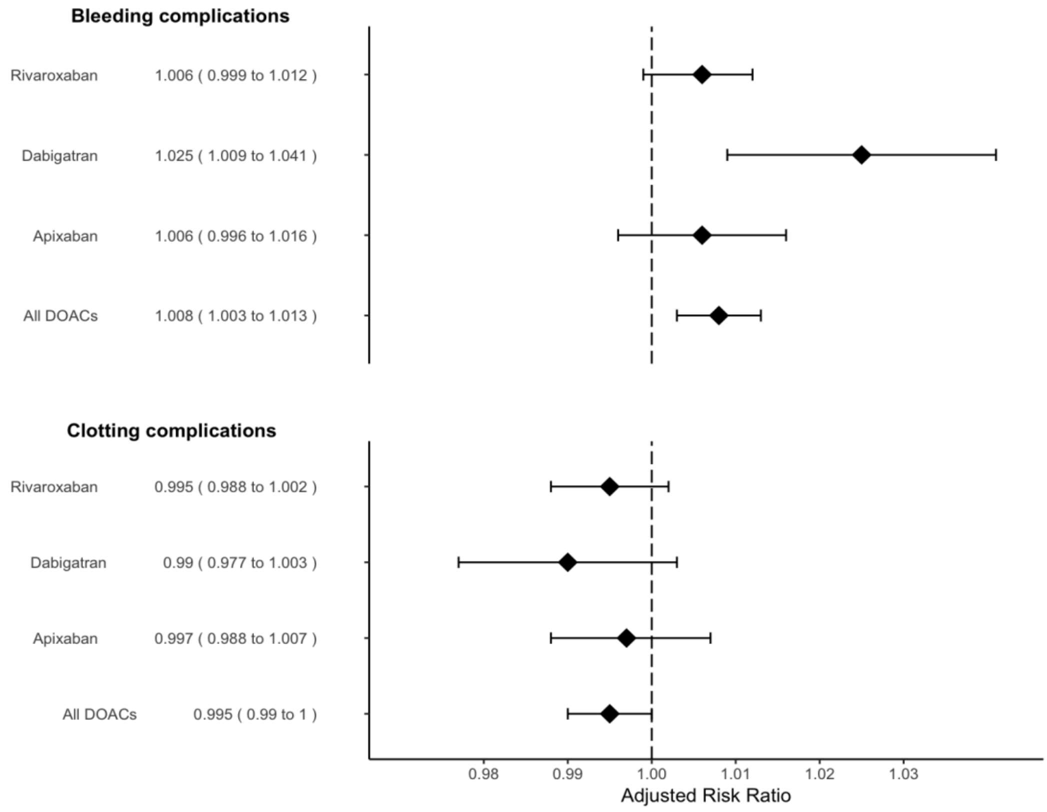

Note: Estimates from a fixed effects Poisson regression model using GP practice panel dataset from 2011 to 2016, also adjusted for the annual prevalence of atrial fibrillation (AF), chronic heart disease (CHD),chronic kidney disease (CKD), the annual proportion of the GP practice over 75 years old and year fixed effects.

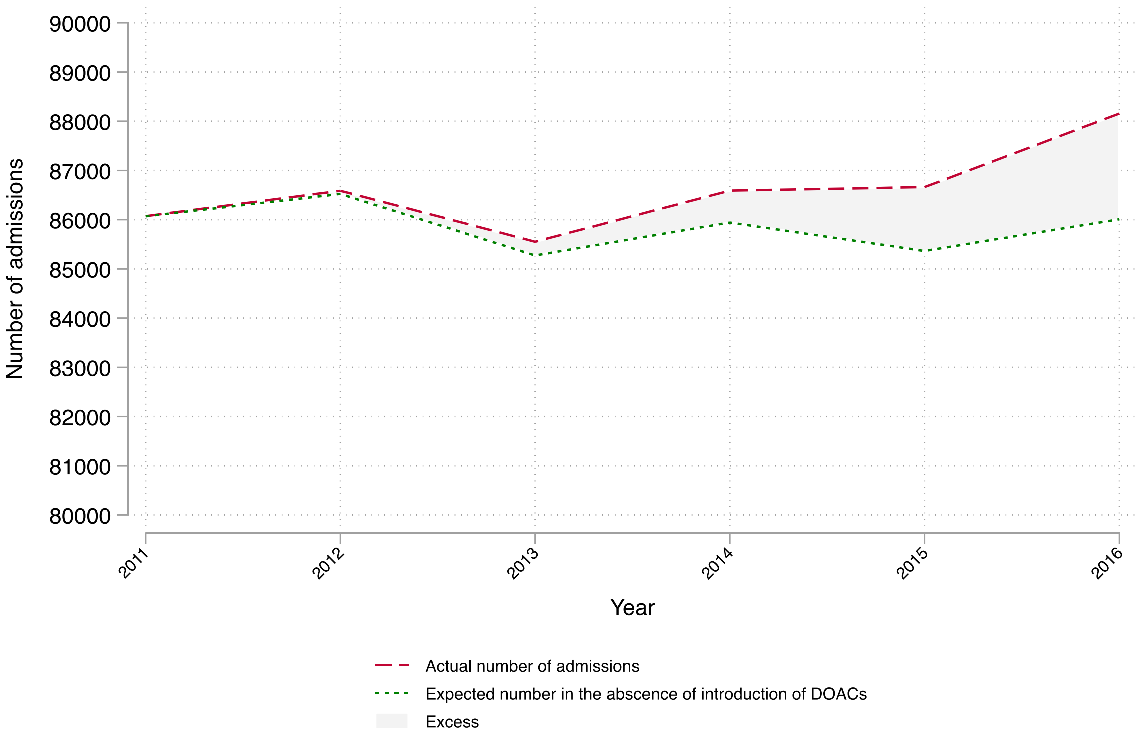

For peer review only

Appendix 1 BNF codes and ICD10 codes used to define exposure and outcome variables.

BNF codes used to define prescribing

0208020V - Warfarin

0208020X0 – Dabigatran

0208020X0 – edoxaban

0208020Y – rivaroxaban

0208020Z0 - Apixiban

ICD 10 Codes used to define outcomes

1. **Bleeding Complications**

ICD10 code	Name
M79.81	traumatic haematoma of the soft tissue
M25.00	Hemarthrosis, unspecified joint
K92	Other diseases of digestive system
K92.0	Haematemesis
K92.1	Melaena
K92.2	Gastrointestinal haemorrhage, unspecified
K62.5	Haemorrhage of anus and rectum
R58	Haemorrhage, not elsewhere classified
R04.0	Epistaxis
R04.1	Haemorrhage from throat
R04.2	Haemoptysis
R04.8	Haemorrhage from other sites in respiratory passages

For peer review only

R04.9	Haemorrhage from respiratory passages, unspecified
R31	Unspecified haematuria
N02	Recurrent or persistent haematuria
I60	Subarachnoid haemorrhage
I60.0	Subarachnoid haemorrhage from carotid siphon and bifurcation
I60.1	Subarachnoid haemorrhage from middle cerebral artery
I60.2	Subarachnoid haemorrhage from anterior communicating artery
I60.3	Subarachnoid haemorrhage from posterior communicating artery
I60.4	Subarachnoid haemorrhage from basilar artery
I60.5	Subarachnoid haemorrhage from vertebral artery
I60.6	Subarachnoid haemorrhage from other intracranial arteries
I60.7	Subarachnoid haemorrhage from intracranial artery, unspecified
I60.8	Other subarachnoid haemorrhage
I60.9	Subarachnoid haemorrhage, unspecified
I61	Intracerebral haemorrhage
I61.0	Intracerebral haemorrhage in hemisphere, subcortical
I61.1	Intracerebral haemorrhage in hemisphere, cortical
I61.2	Intracerebral haemorrhage in hemisphere, unspecified
I61.3	Intracerebral haemorrhage in brain stem
I61.4	Intracerebral haemorrhage in cerebellum
I61.5	Intracerebral haemorrhage, intraventricular
I61.6	Intracerebral haemorrhage, multiple localized
I61.8	Other intracerebral haemorrhage
I61.9	Intracerebral haemorrhage, unspecified
I62	Other nontraumatic intracranial haemorrhage
I62.0	Subdural haemorrhage (acute)(nontraumatic)
I62.1	Nontraumatic extradural haemorrhage

For peer review only

I62.9	Intracranial haemorrhage (nontraumatic), unspecified

2. **Clotting Complications**

**ICD10 code**	**Name**
I63	Cerebral infarction
I64	Stroke, not specified as haemorrhage or infarction
I26	Pulmonary embolism
I82	Other venous embolism and thrombosis
I80.0	Phlebitis and thrombophlebitis of superficial vessels of lower extremities
I80.1	Phlebitis and thrombophlebitis of femoral vein
I80.2	Deep vein thrombosis, Phlebitis and thrombophlebitis of other deep vessels of lower extremities
I80.3	Phlebitis and thrombophlebitis of lower extremities, unspecified
I80.8	Phlebitis and thrombophlebitis of other sites
I80.9	Phlebitis and thrombophlebitis of unspecified site

For peer review only

Appendix 2 flow outlining inclusion/ exclusion and missing data

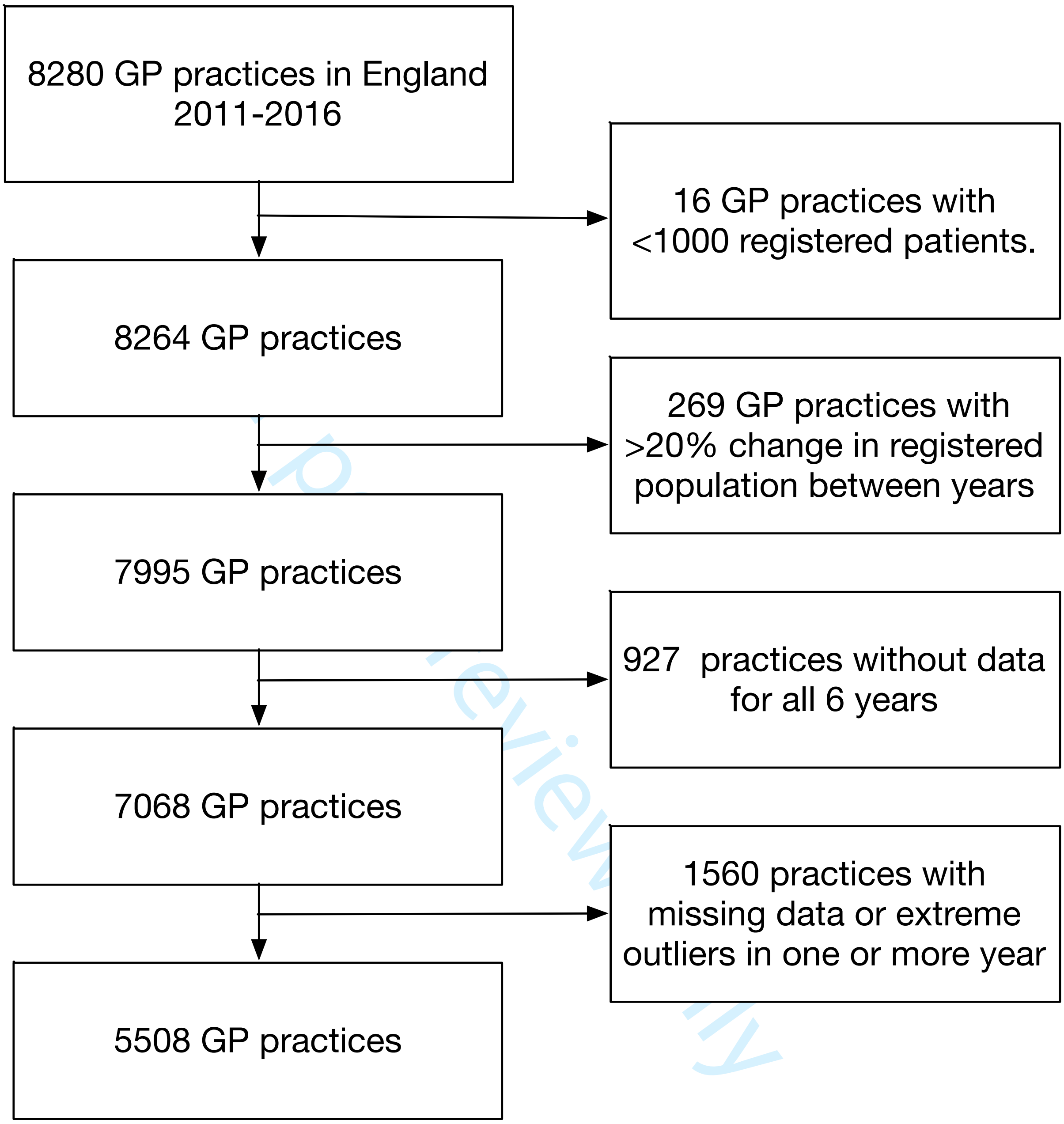

Appendix 3 alternative model specifications.

1. **Model using all GP practices – without applying exclusion criteria (8032 GP practices – 248 with missing data). Estimates from regression model showing the relative change in the rate of emergency admissions for bleeding and clotting complications associated with each additional 10% of DOACs prescribed as a proportion of all anticoagulants**

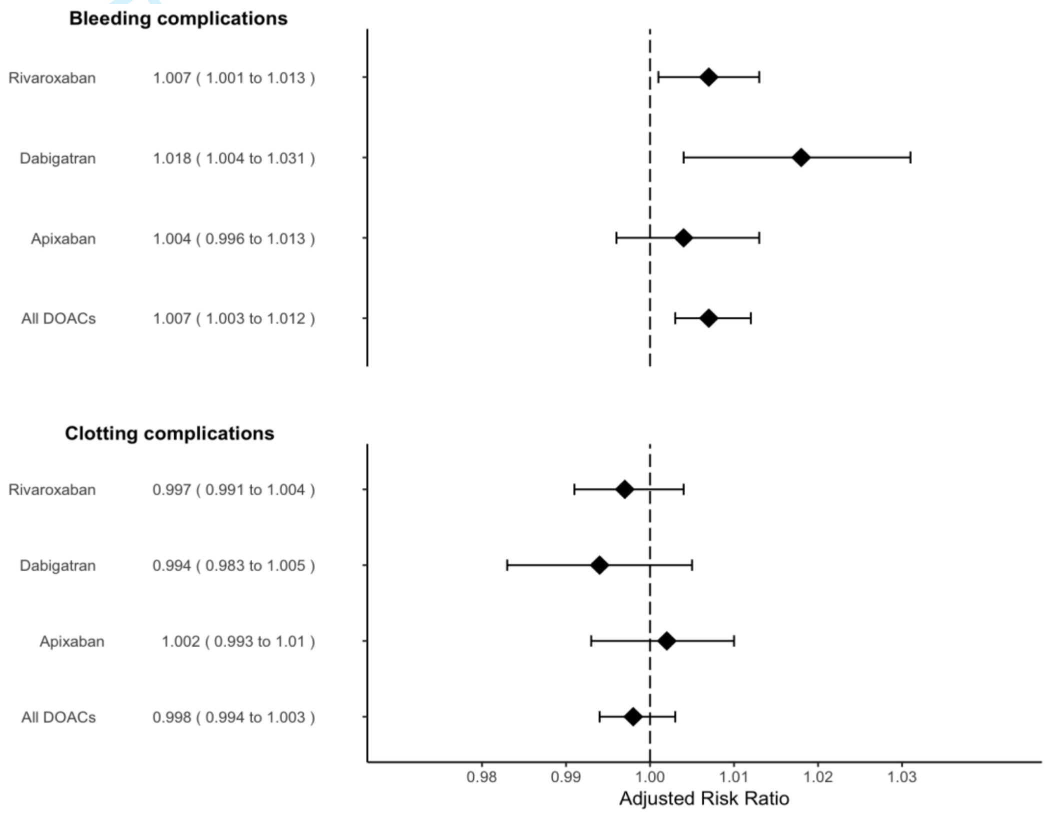

2. Negative Binomial Model

Estimates from regression model showing the relative change in the rate of emergency admissions for bleeding and clotting complications associated with each additional 10% of DOACs prescribed as a proportion of all anticoagulants

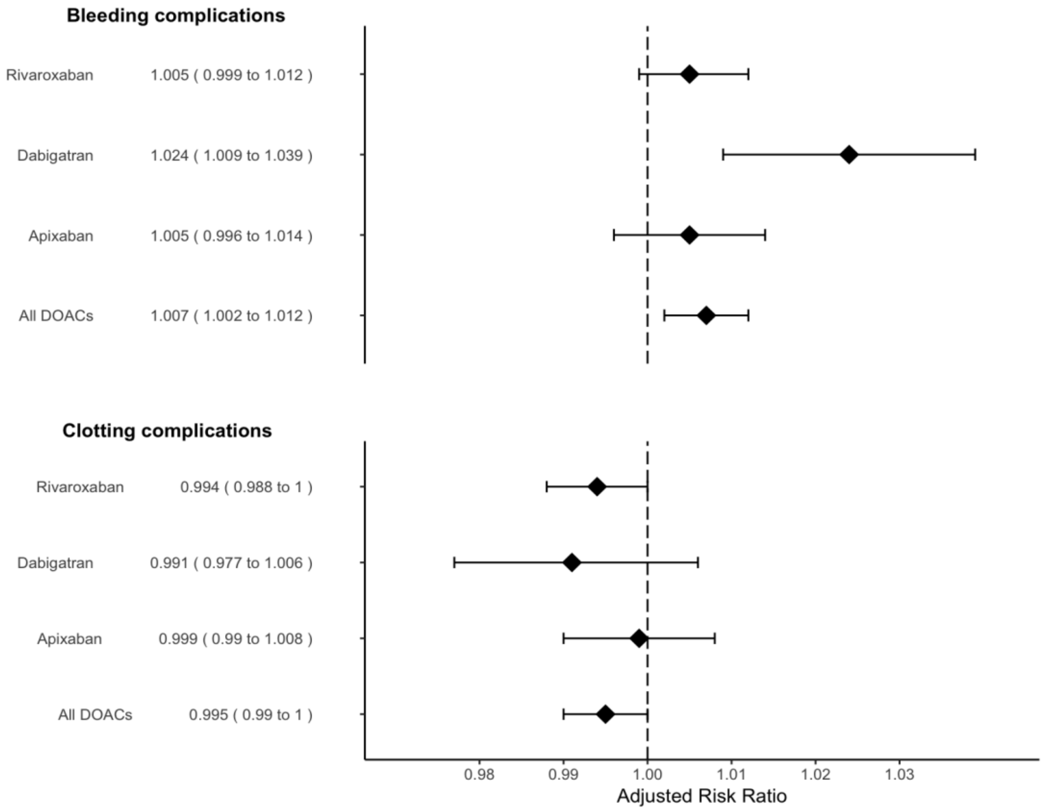

only

3. Non-equivalent dependent variable.

Estimates from regression model showing the relative change in the rate of emergency admissions for gastrointestinal admissions associated with each additional 10% of DOACs prescribed as a proportion of all anticoagulants.

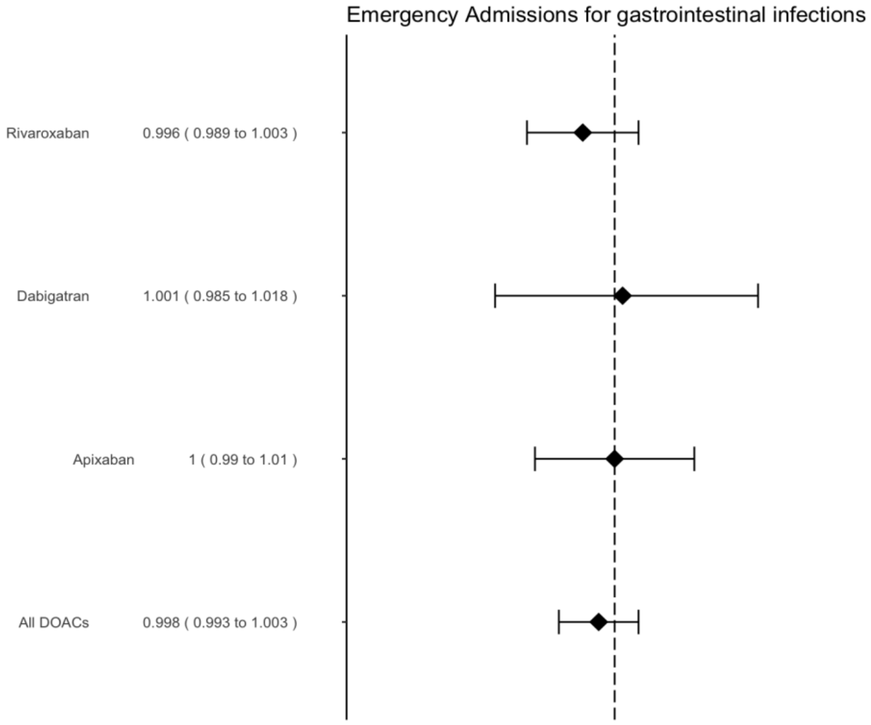

STROBE Statement—checklist of items that should be included in reports of observational studies
Paper submission:
Alfirevic et al. Has the introduction of direct oral anticoagulants (DOACs) in England increased emergency admissions for bleeding conditions? A longitudinal ecological study.

	Item No	Recommendation
**Title and abstract**	1	(*a*) We've indicated the study's design in the title or the abstract
		(*b*) We've provided in the abstract an informative and balanced summary of what was done and what was found, page 2
**Introduction**		
Background/rationale	2	We've explained the scientific background and rationale for the investigation being reported, pages 3 and 4
Objectives	3	State specific objectives, including any prespecified hypotheses- done, page 4, line 12 to 18
**Methods**		
Study design	4	Key elements of study design were presented on page 5, lines 12 to 27
Setting	5	The setting, locations, and relevant dates, including periods of recruitment, exposure, follow-up, and data collection were presented on pages 4 and 5
Participants	6	We included all GP practices in England with a registered population size greater than 1000, which had data for all 6 years (N=5508 GP practices with 33,048 practice years of data. Page 5
		Inclusions/exclusions and missing data are outlined in Supplementary material- Appendix 2
Variables	7	Clearly defined outcomes (BNF and ICD10 codes) are presented in Supplementary material, Appendix 1
Data sources/ measurement	8*	Sources of data and details of methods of assessment are presented on pages 4 and 5. Describe comparability of assessment methods if there is more than one group
Bias	9	Potential sources of bias such as confounding from unobserved factors that vary between GP practice populations, we used a fixed effects approach and a dummy variable for each year to account for the national trend in prescribing and emergency admissions. In addition, we performed sensitivity analyses
Study size	10	All eligible GP practices in England were included in this study
Quantitative variables	11	We've explained how quantitative variables were handled in the analyses on pages 5 and 6
Statistical methods	12	(*a*) Describe all statistical methods, including those used to control for confounding- done, page 5 and 6
		(*b*) Describe any methods used to examine subgroups and interactions, done page 5 and 6
		(*c*) Explain how missing data were addressed- done flow chart Supplementary material Appendix 2
		(*e*) Sensitivity analyses are described on page 6

Continued on next page

For peer review only - http://bmjopen.bmj.com/site/about/guidelines.xhtml

**Results**		
Participants	13*	(a) Report numbers of individuals at each stage of study-done, Supplementary material Appendix 2
		(b) Give reasons for non-participation at each stage- done Appendix 2
		(c) Flow diagram- done and shown in Appendix 2
Descriptive data	14*	(a) Give characteristics of study participants – GP practice data included, no data on individual participants are available
		(b) We've indicated the number of practices with missing data in Appendix 2
		(c) *F*ollow-up time of 6 years has been clearly shown in the manuscript (page 5 and 7) and Figures 1 and 2
Outcome data	15*	
		Outcome data are described in the manuscript, page 7
Main results	16	(*a*) We have given unadjusted estimates and their precision (95% confidence interval), page 7
		(*c*) We haven't translated estimates of relative risk into absolute risk
Other analyses	17	We have reported sensitivity analyses, page 7, lines 50-59
**Discussion**		
Key results	18	Summarise key results with reference to study objectives- done, page 8
Limitations	19	Discuss limitations of the study, taking into account sources of potential bias or imprecision. Discuss both direction and magnitude of any potential bias- done page 9 line 22
Interpretation	20	Give a cautious overall interpretation of results considering objectives, limitations, multiplicity of analyses, results from similar studies, and other relevant evidence done page 10
Generalisability	21	Discuss the generalisability (external validity) of the study results – we have compared our results with previous studies, page 10
**Other information**		
Funding	22	Give the source of funding and the role of the funders for the present study and, if applicable, for the original study on which the present article is based- Done, we have included a sentence at the end of the manuscript to acknowledge our NIHR CLAHRC NWC funding

*Give information separately for cases and controls in case-control studies and, if applicable, for exposed and unexposed groups in cohort and cross-sectional studies.

Note: An Explanation and Elaboration article discusses each checklist item and gives methodological background and published examples of transparent reporting. The STROBE checklist is best used in conjunction with this article (freely available on the Web sites of PLoS Medicine at http://www.plosmedicine.org/, Annals of Internal Medicine at http://www.annals.org/, and Epidemiology at http://www.epidem.com/). Information on the STROBE Initiative is available at www.strobe-statement.org.

For peer review only

BMJ Open

Has the introduction of direct oral anticoagulants (DOACs) in England increased emergency admissions for bleeding conditions? A longitudinal ecological study.

Journal:	*BMJ Open*
Manuscript ID	bmjopen-2019-033357.R1
Article Type:	Original research
Date Submitted by the Author:	22-Nov-2019
Complete List of Authors:	Alfirevic, Ana; University of Liverpool, Molecular and Clinical Pharmacology<br>Downing, Jennifer; University of Liverpool, Molecular and Clinical Pharmacology<br>Daras, Konstantinos ; University of Liverpool School of Environmental Sciences, Geography and Planning<br>Comerford, Terence; University of Liverpool<br>Pirmohamed, Munir; University of Liverpool, Molecular and Clinical Pharmacology<br>Barr, Ben
**Primary Subject Heading**:	Public health
Secondary Subject Heading:	Pharmacology and therapeutics
Keywords:	CLINICAL PHARMACOLOGY, PRIMARY CARE, PUBLIC HEALTH

SCHOLARONE™
Manuscripts

I, the Submitting Author has the right to grant and does grant on behalf of all authors of the Work (as defined in the below author licence), an exclusive licence and/or a non-exclusive licence for contributions from authors who are: i) UK Crown employees; ii) where BMJ has agreed a CC-BY licence shall apply, and/or iii) in accordance with the terms applicable for US Federal Government officers or employees acting as part of their official duties; on a worldwide, perpetual, irrevocable, royalty-free basis to BMJ Publishing Group Ltd ("BMJ") its licensees and where the relevant Journal is co-owned by BMJ to the co-owners of the Journal, to publish the Work in this journal and any other BMJ products and to exploit all rights, as set out in our licence.

The Submitting Author accepts and understands that any supply made under these terms is made by BMJ to the Submitting Author unless you are acting as an employee on behalf of your employer or a postgraduate student of an affiliated institution which is paying any applicable article publishing charge ("APC") for Open Access articles. Where the Submitting Author wishes to make the Work available on an Open Access basis (and intends to pay the relevant APC), the terms of reuse of such Open Access shall be governed by a Creative Commons licence – details of these licences and which Creative Commons licence will apply to this Work are set out in our licence referred to above.

Other than as permitted in any relevant BMJ Author's Self Archiving Policies, I confirm this Work has not been accepted for publication elsewhere, is not being considered for publication elsewhere and does not duplicate material already published. I confirm all authors consent to publication of this Work and authorise the granting of this licence.

For peer review only

Has the introduction of direct oral anticoagulants (DOACs) in England increased emergency admissions for bleeding conditions? A longitudinal ecological study.

Ana Alfirevic[1], Jennifer Downing[1], Konstantinos Daras[2], Terence Comerford[3], Munir Pirmohamed[1] and Ben Barr[2]

[1] The Wolfson Centre for Personalised Medicine, Department of Molecular and Clinical Pharmacology, Institute of Translational Medicine, University of Liverpool, UK

[2] Department of Public Health and Policy, Institute of Psychology, Health and Society, University of Liverpool, UK

[3] CLAHRC NWC Public Advisor Group, University of Liverpool, UK

Corresponding Author:

Ana Alfirevic

The Wolfson Centre for Personalised Medicine,

Department of Molecular and Clinical Pharmacology,

Institute of Translational Medicine

University of Liverpool

Block A, The Waterhouse Building

1-5 Brownlow Street

Liverpool, L69 3GL

Tel: +44 (0)151 7945551

E-mail: Ana.Alfirevic@liv.ac.uk

For peer review only

For peer review only - http://bmjopen.bmj.com/site/about/guidelines.xhtml

ABSTRACT

Objective. There is concern about long term safety of direct oral coagulants (DOACs) in clinical practice. Our aim was to investigate whether the introduction of DOACs compared with vitamin-K antagonists in England was associated with a change in admissions for bleeding or thromboembolic complications.

Setting: 5508 General Practitioner (GP) practices in England between 2011 and 2016.

Participants: All GP practices in England with a registered population size of greater than 1000 that had data for all 6 years.

Main outcome measure: The rate of emergency admissions to hospital for bleeding or thromboembolism, per 100,000 population for each GP practice in England.

Main exposure measure: The annual number of DOAC items prescribed for each GP practice population as a proportion of all anticoagulant items prescribed.

Design. This longitudinal ecological study used panel regression models to investigate the association between trends in DOAC prescribing within GP practice populations and trends in emergency admission rates for bleeding and thromboembolic conditions, whilst controlling for confounders.

Results. For each additional 10% of DOACs prescribed as a proportion of all anticoagulants, there was a 0.7% increase in bleeding complications (rate ratio (RR) 1.007 95% confidence intervals (CI) 1.002 to 1.012). The introduction of DOACs between 2011 and 2016 was associated with additional 4929 (95%CI 2489 to 7370) emergency admissions for bleeding complications. Increased DOAC prescribing was associated with a slight decline in admission for thromboembolic conditions.

Conclusion. Our data show that the rapid increase in prescribing of DOACs after changes in the NICE guidance in 2014 may have been associated with a higher rate of emergency admissions for bleeding conditions. These consequences need to be considered in assessing the benefits and costs of the widespread use of DOACs.

Word count 282

Key words Direct oral anticoagulants, warfarin, emergency admissions, bleeding, thromboembolism

For peer review only

Article Summary

Strengths and limitations of this study

- The majority of GP practices in England were included in our analyses and therefore we could estimate effects across the whole population
- Longitudinal data analyses included the changes in the guidelines for prescribing anticoagulants
- We used effectiveness and safety data on anticoagulants used in England
- It was not possible to link information on the prescribing for specific individuals to particular hospital admissions due to the ecological study design
- No control for adherence to medications was possible

For peer review only

For peer review only - http://bmjopen.bmj.com/site/about/guidelines.xhtml

Introduction

Prescribing of direct oral anticoagulants (DOACs) such as dabigatran, rivaroxaban, apixaban and edoxaban that were licenced between 2013 and 2015, has increased rapidly in the UK. DOACs are used for stroke prevention in atrial fibrillation (AF), treatment of venous thromboembolism (VTE), and medical and surgical thromboprophylaxis.

Warfarin has been the primary oral anticoagulant in patients with AF and VTE for over 60 years. Its use is associated with large inter-individual variability in dose requirements, narrow therapeutic index, slow onset of action, low time in therapeutic range and concomitant drug and food interactions. In addition, regular International Normalised Ratio (INR) monitoring required for optimising loading and maintenance doses is burdensome to the patients and healthcare professionals. The DOACs are recommended by NICE guidelines for the treatment of adults with venous thromboembolism, prevention of recurrent deep vein thrombosis, and stroke prevention in patients with non-valvular atrial fibrillation[1]. Several non-inferiority randomised controlled trials have demonstrated that DOACs have comparable efficacy and safety profiles with warfarin [2-5]. In addition, in clinical trials fixed-dose administration was proposed for DOACs, without the need for routine laboratory monitoring. This has led to widespread uptake of DOACs into clinical practice. Although DOACs have been shown to be cost-effective in RCTs[6], their high cost compared with the cost of warfarin (inclusive of INR testing) has major budget implications for the NHS in the UK and other healthcare services globally[7]. Concern about the escalating costs to healthcare is increasing not only for DOACs, but also when complications occur, for their reversal agents such as idarucizumab or andexanet alpha[8].

Data on the comparative safety and efficacy of DOACs in real-life observational studies has also become available more recently[6]. For instance, a recent large observational study in the UK found that apixaban was associated with decreased risk of bleeding complications, but rivaroxaban and low dose apixaban were associated with an increased risk of all-cause mortality compared to warfarin[1]. A large study (>14,500 patients) conducted in Scotland demonstrated that patients taking rivaroxaban may be

For peer review only - http://bmjopen.bmj.com/site/about/guidelines.xhtml

at increased risk of bleeding compared with other DOACs[9]. Recent concern about bleeding risks associated with DOACs has prompted the EMA to launch a safety review (https://www.ema.europa.eu/documents/other/direct-oral-anticoagulants-doacs-ema-starts-review-study-bleeding-risk-direct-oral-anticoagulants_en.pdf).

In this study we determined whether differences in prescribing of DOACs compared to warfarin between General Practitioner (GP) practices across England has been associated with variation of trends in emergency hospital admissions for bleeding and thromboembolic events from GP practice registered populations.

Methods

Setting and data sources.

We identified DOACs as dabigatran etexilate, apixaban, edoxaban or rivaroxaban using the BNF codes given in Appendix 1. We used the following NHS Digital data:

1) Practice level prescribing data that are published and made available by the NHS Business Service Authority (BSA) each month. We used the Practice Prescribing Data File with the full 15-digit BNF code to calculate the annual DOAC prescribing rate as the percentage of DOAC items prescribed in proportion to all anticoagulants items prescribed for each GP practice in England between 2011 and 2016. The prescribing rate for each separate DOAC (dabigatran etexilate, apixaban, edoxaban or rivaroxaban) was calculated as the percentage of items of these medications prescribed as a proportion of all anticoagulants items prescribed. A prescription item refers to a single item prescribed on a prescription form, generally a course of medicine and is routinely used to measure trends in prescribing. We used the Net Ingredient Costs of these items included in this data set to calculate the prescribing costs.

2) Hospital Episode Statistics data were used to calculate the annual emergency admission rate for bleeding and clotting conditions for each GP practice. The ICD10 diagnostic codes included in this

For peer review only - http://bmjopen.bmj.com/site/about/guidelines.xhtml

indicator is given in Appendix 1. Rates per 100,000 were calculated using annual data on the number of people registered with each GP practice provided by NHS Digital.

To additionally control for trends in the population age profile and underlying trends in morbidity, we also calculated the annual proportion of a GP population that was over 75 and used annual data on the prevalence of Atrial Fibrillation (AF), Coronary Heart Disease (CHD) and Chronic Kidney Disease (CKD) for each GP practice population using data reported through the Quality and Outcome Framework (QOF) returns.

We included all GP practices in England with a registered population size of greater than 1000 that had data for all 6 years. We excluded GP practices with values for prevalence estimates from QOF that were clear outliers (more than 2 interquartile ranges (IQRs) below the first quartile or above the third quartile) as these probably represent reporting errors. In addition, we excluded practices where the practice registered population has changed by more than 20% between consecutive years as this would have reflected a large change in the practice population probable due to practice mergers or closures. This provided 5508 practices for the final analysis, each providing 6 years of data – i.e. 33,048 practice years of data. A flow chart is given in Appendix 2 detailing exclusions.

Analyses

Initially, we investigated geographical pattern of increases in DOACs prescribing across England. We mapped GP level prescribing data to Lower Super Output Areas (LSOA), based on the proportion of each GP practice's population that lived in each LSOA. Next, we plotted national maps for the DOAC prescribing rate each year. We then used a fixed effects Poisson regression model to investigate the association between the trend in prescribing within a GP practice and the trend in the rate of admissions for bleeding conditions. The outcome measure was the number of admissions and the log of the GP registered population was used as an offset. As there is potential confounding from unobserved factors that vary between GP practice populations, we used a fixed effects approach to remove these between-GP practice differences[10]. This conservative approach is the equivalent to including dummy variables

For peer review only - http://bmjopen.bmj.com/site/about/guidelines.xhtml

for each GP practice so that the model assesses the association between the trend in prescribing and the trend in hospital admissions within each GP practice. We additionally included a dummy variable for each year to account for the national trend in prescribing and emergency admissions. To additionally control for differential trends in need for anticoagulants and risk of bleeding complications in each GP population, we included annual measures of the prevalence of AF, CHD and CKD and the proportion of the GP population over 75-year olds. We used Huber-White clustered standard errors that are robust to divergence of the data from the assumptions of a Poisson distribution and the clustering of variance between GP practices[11]. To investigate whether there were different effects associated with different types of DOACs, we repeated the analysis for dabigatran, apixaban and rivaroxoban. As edoxaban was only licenced in the middle of 2015, there was insufficient data to analyse this separately.

Ethical approval was not required for this ecological study data analyses.

Sensitivity analysis

We subjected our analysis to a number of tests to assess the robustness of our findings. We estimated a negative binomial model as opposed to a Poisson model which is more robust to over-dispersion in the data, estimated General Estimating Equations with exchangeable and autoregressive correlation structures and replicated our models using data from all GP practices (i.e. not applying any exclusion). To test the specificity of results, we estimated our model using a gastro-intestinal infection as a Nonequivalent Dependent Variables (NDV)[12]. This outcome should *not* be influenced by a change in the exposure but could be influenced by unobserved confounding factors that influence general trends in hospitalization in a GP practice population.

Patient involvement

No patients were involved in setting the research question or the outcome measures, nor were they involved in developing plans for design or implementation of the study. Patients and patient representatives within the NIHR CLAHRC NWC were asked to revise the manuscript in particular the lay language summary. The results of the study are going to be disseminated to the relevant patient and public groups through the CLAHRC NWC communication programme.

For peer review only - http://bmjopen.bmj.com/site/about/guidelines.xhtml

Results

Figure 1 shows the trend in the number of items and net drug costs of DOACs prescribed in England by GP practices between 2011 and 2016. Between 2011 and 2016, the number of items of anticoagulants prescribed had risen from 0.7 million to 1.4 million per month and the proportion of these that were DOACs had risen from <1% to 35%. Costs of anticoagulants rose more steeply, from £1.6 million per month in 2011 to £26 million per month by the end of 2016. This rise in costs was due to increased prescribing of DOACs, which by the end of 2016 accounted for 94% of the cost of all anticoagulant prescribing.

Figure 2 shows the geographical pattern of DOAC prescribing each year. The increase in uptake of DOACs has not been consistent across the country, with the prescribing rate increasing markedly more in some part of the country compared to others. In particular, areas around Somerset, Kent, and to a lesser extent the North East, appeared to have adopted DOACs sooner than other areas. By 2016 there was markedly lower usage of DOACs in the North West and East Anglia.

The results of the regression model indicate that differences in the trends in DOAC prescribing between GP practices from 2011 to 2016 were associated with differences in the trends in admissions for bleeding conditions. For each additional 10% of DOACs prescribed as a proportion of all anticoagulants, there was a 0.7% increase in emergency admissions for bleeding complications (RR:1.007 95% CI 1.002 to 1.013). We present the effect estimates per additional 10% of DOACs prescribed as this reflects a more meaningful level of increase than a 1 percentage point increase. Increased risk of emergency admissions for bleeding complications was particularly associated with increased use of dabigatran. Increased use of DOACs was associated with a small decline in emergency admissions for clotting related conditions; however this was not statistically significant (p=0.065), see Figure 3.

Figure 4 shows the trend in emergency admissions for bleeding conditions between 2011 and 2016 and the trend, estimated from the regression model that would have been expected if DOACs had not been introduced. Overall the introduction of DOACs between 2011 and 2016 was associated with 1.94

For peer review only

additional emergency admissions for bleeding complications per 100,000 people per year [0.98 to 2.9], the equivalent to a total of 4929 emergency admissions for bleeding complications (95% CI 2489 to 7370).

Our results were similar when using alternative model specifications (see Appendix 3). We found similar results when using a negative binomial model as opposed to a Poisson regression, when using General Estimating Equations with alternative correlation structures and when replicating our model using data from all GP practices (i.e. not applying any exclusions). We found no association between the trend in DOAC prescribing and emergency admissions when applying the model with a non-equivalent dependant variable (gastro-intestinal infections).

Discussion

In this study, we demonstrated a sharp increase in the uptake of DOACs by general practitioners in the UK between 2011 and 2016, compared with the current standard, warfarin. The uptake of DOACs has not been consistent and there are geographical areas with markedly higher rate of DOAC prescribing. Worryingly, higher rates of DOAC prescribing were associated with higher rates of hospital admissions for bleeding events, with a slight decline in emergency admissions for thromboembolic complications. We projected that with every 10% increase of DOAC prescribing as a proportion of all anticoagulants, there was a 0.7% increase in emergency admissions for bleeding complications. Moreover, we estimated from our regression model that between 2011 and 2016, there were an additional 4929 emergency admissions for bleeding complications, more than would have been expected if DOACs had not been introduced. At the end of 2016, 94% of the total expenditure on anticoagulants prescribed by GP practices in England was spent on DOACs, which represented only 35% of all prescribed anticoagulants.

The strengths and limitations of the study

The strength of this study is that we analysed data over several years that included the changes in the guidelines for prescribing anticoagulants. In 2014, the UK National Institute for Health and Care Excellence (NICE) guidance recommended the use of DOACs and warfarin taking into consideration

For peer review only - http://bmjopen.bmj.com/site/about/guidelines.xhtml

clinician and patient preferences. Comparable efficacy and the evidence from RCTs of better safety of DOACs compared with warfarin, combined with no need for regular monitoring and a wider therapeutic range, resulted in a major increase in prescribing[1 3 4 6]. We captured this increase in our longitudinal analyses. Our sample includes the majority of GP practices in England and is therefore able to estimate effects across the whole population. Our model assessed the association between the trend in prescribing and the trend in hospital admissions within each GP practice and we included a dummy variable for each year to account for the national trends. Any bias in our model of the association between our main exposure variable and outcome is therefore unlikely to be the result of time-invariant confounders that differ between GP practice population, or from any changes over time that effect all GP practices equally. We found a similar effect when using alternative models. When replicating the analysis with a common emergency admission we would not expect to be affected by DOAC prescribing such as gastrointestinal infections, we found, as expected, no association. Additional strength of our study is that we included in our analyses both, effectiveness and safety data on anticoagulants used in England. We investigated whether increased bleeding risk has been compensated by a reduction in risk from clotting conditions and found that increased DOAC prescribing was associated with some reduction in emergency admission from thromboembolic conditions however, this effect was small and not statistically significant.

A number of limitations remain. Firstly, the ecological design meant that it was not possible to link information on the prescribing for specific individuals to particular hospital admissions. Therefore the associations observed at the aggregate level may not reflect associations at the level of the individual patients. Secondly, whilst our analysis adjusts for a number of observed and unobserved confounders, it is still possible that there are unobserved trends in factors that increase DOAC prescribing whilst also increasing risk of bleeding complications. Bias could be introduced for example if there are differential trends between GP practices in underlying risk of bleeding complications, that are not accounted for by our control variables and this increased risk of bleeding complications led to increased DOAC prescribing in these practices. As our outcomes were measured at the population level our study will, however, be less at risk of selection bias, than studies using individual follow up, where this results

For peer review only - http://bmjopen.bmj.com/site/about/guidelines.xhtml

from clinical decisions to preferentially prescribe DOACs to patients with higher risk of bleeding complications. Thirdly, we could not control for adherence to medications in our study. Adherence to anticoagulants has been reviewed recently[13] with some contradictory reports. Although it is expected that adherence would be better for DOACs because of no need for regular monitoring, regular INR tests required to ensure the correct dose of warfarin lead to stringent check-ups for adherence, while DOACs monitoring is not recommended and therefore adherence declines after the initial period[14]. With long term adherence monitoring our estimates would have been more precise.

Comparisons with previous studies

Our results differ from the findings of several large-scale non-inferiority RCTs that have demonstrated better bleeding profiles of DOACs over warfarin[5 15-20]. This may not be surprising as trial data may provide limited information on relatively rare adverse effects due to low power and short follow up. In addition, participants in clinical trials may be younger with a fewer comorbidities. For example, renal impairment that occurs in the older population may be relevant. Renal clearance is more dominant for DOACs compared with warfarin, as 80%, 50%, 36% and 27% of unchanged dabigatran, edoxaban, rivaroxaban and apixaban respectively, are excreted in urine[21]. In addition, there is limited information available on the risk-benefit profile of DOACs in patients with severe renal function impairment[22-25] particularly in patients with diverse ethnic backgrounds[26]. Recently, high inter-individual variability in DOAC plasma levels was observed in clinical practice [27].

Our findings also differ from the results of a recent retrospective cohort study in patients with intracranial haemorrhage who were previously prescribed warfarin or DOACs. Prior use of DOACs compared with prior use of warfarin was associated with lower risk of hospital mortality[28]. A recent network meta-analysis by Lopez-Lopez also showed that DOACs are safer than warfarin in relation to major and intracranial bleeding[6]. However interestingly, in that meta-analysis the risk of gastrointestinal bleeding was higher with dabigatran, edoxaban and rivaroxaban than with warfarin. In addition, edoxaban (30mg and 60 mg twice daily) significantly increased the risk of clinically relevant bleeding compared with warfarin[6]. Although our study uses a longitudinal ecological study design, at least in

For peer review only - http://bmjopen.bmj.com/site/about/guidelines.xhtml

part, our results are comparable with previous studies. In a large (>59,000 participants) population-based observational study conducted in Canada and the US, the risk of hospital admissions for major bleeding or all-cause mortality in the first 90 days of treatment was similar for DOACs and warfarin [29]. Furthermore, in a study that used an US commercial database of 38 million people warfarin users were hospitalised longer, stayed longer in an intensive care units than dabigatran or rivaroxaban users, but there was no difference in 30- or 90-day all-cause mortality[30].

It has been estimated that the cost of DOACs will rise sharply before patent expiry (in 2022) and by the year 2020 will constitute approximately 5% of the total NHS drug budget[14]. The drugs budget is the NHS's second biggest cost after its staff[31]. Cost of warfarin including INR monitoring for one patient per annum has been estimated to be £220 (https://www.gwh.nhs.uk/media/236108/doacs-for-dvt-pe-august-2016-v-9.pdf), while the cost of rivaroxaban (15mg bd for 3/52 loading (provided by hospital), then 20mg o.d. has been estimated to £657 p.a. (https://www.gwh.nhs.uk/media/236108/doacs-for-dvt-pe-august-2016-v-9.pdf). Similar costs have been estimated for other DOACs. Additionally, there is a huge discrepancy in price of agents that are used to reverse the anticoagulant effects of warfarin (antidote vitamin K at £0.38) or DOACs (idarucizumab, reversal agent for dabigatran at £2,400 for a single treatment course of 2X 2.5 g infusions (https://www.nice.org.uk/advice/esnm73) or andexanet alpha, reversal agent for factor Xa inhibitors approved by the FDA in May 2018, at approximately £1,500). These high costs of DOACs could be avoided if warfarin treatment is optimised, for example through novel methods such as genotype-guided dosing[32 33] and point-of-care INR monitoring[34], and only individuals who are at higher risk of developing bleeding events, for example those with variant alleles that increase the risk of bleeding from warfarin[35], could be prescribed DOACs. Genotype-guided dosing of warfarin has been shown to be cost-effective[36]. It has been shown previously that approximately 55% of variability in warfarin dose requirements can be estimated from clinical and genetic data of three polymorphisms in the warfarin molecular target (vitamin K epoxide reductase gene) and metabolising enzyme (CYP2C9 gene) and genotype-guided dosing can reduce the risk of major bleeding[37 38].

For peer review only - http://bmjopen.bmj.com/site/about/guidelines.xhtml

Conclusions

This study is one of a few studies in the UK[1 39 40] that have evaluated the association between the rise in prescribing of DOACs after changes in NICE guidance in 2014 and emergency hospital admissions for bleeding events. We found that the rate of emergency admissions for bleeding conditions increased to a greater extent in GP practices that were more likely to prescribe DOACs compared with warfarin. With rapidly increasing use of DOACs, these potential adverse consequences need to be taken into account when assessing the benefits and costs of anticoagulant treatment in clinical practice. It is not clear whether the DOACs are being prescribed without adequate notice being taken of restrictions and warnings in the summary of product characteristics, and in addition, whether closer monitoring is required in certain patient groups to increase the safety of use in clinical practice. The recently announced review by the EMA is thus important in order to further optimise the use of all oral anticoagulants so that risks are minimised while at the same time maximising benefits.

PPI statement

The study was supported by a patient advisory group, which provided input to the NIHR- CLAHRC NWC programme of research. This patient advisory group met on a regular basis for the duration of the study. Due to data protection restrictions and the very technical methods required to do our data analyses they were not involved in the analysis part of the project. PPI representatives will however, write a plain language summary and design a leaflet for dissemination to their peers and distributing to patient groups.

Author contributions

Ana Alfirevic- Substantial contributions to the conception and design of the work, drafting a manuscript. Agreed to be accountable for all aspects of the work in ensuring that questions related to the accuracy or integrity of any part of the work are appropriately investigated and resolved.

Ben Barr- Substantial contribution to the design of the work, the acquisition, analysis and interpretation of data. Agreed to be accountable for all aspects of the work in ensuring that questions related to the accuracy or integrity of any part of the work are appropriately investigated and resolved.

Munir Pirmohamed- Substantial contribution to the interpretation of the results, critical revision of the manuscript for important intellectual content and final approval of the manuscript.

Jennifer Downing- Critical revision of the manuscript for important intellectual content and final approval of the manuscript.

For peer review only - http://bmjopen.bmj.com/site/about/guidelines.xhtml

Konstantinos Daras- Substantial contribution to data acquisition, data analysis and interpretation of data for the work. Critical revision of the manuscript for important intellectual content and final approval of the manuscript

Terence Comerford- Critical revision of the manuscript for important intellectual content and final approval of the manuscript. Preparation of plain language summary for dissemination of findings.

Patient consent for publication: *Not required.*
Funding: *This work was supported by the National Institute for Health Research, Collaboration for Leadership and Health Research and Care North West Coast (NIHR CLAHRC NWC) and the state Department of Health and Social Care.*
Disclaimer: *The views expressed are those of the author(s) and not necessarily those of the NHS, the NIHR or the Department of Health.*
Competing interests: *We have read and understood BMJ policy on declaration of interests and declare the following: All authors have completed the ICMJE uniform disclosure form at http://www.icmje.org/coi_disclosure.pdf and declare: no support from any organisation for the submitted work [or describe if any]; no financial relationships with any organisations that might have an interest in the submitted work in the previous three years [or describe if any], no other relationships or activities that could appear to have influenced the submitted work [or describe if any].*

Provenance and peer review: *Not commissioned; externally peer reviewed.*
Data sharing statement: *The dataset from this study will be made available on request to benbarr@liverpool.ac.uk*

References

1. Vinogradova Y, Coupland C, Hill T, et al. Risks and benefits of direct oral anticoagulants versus warfarin in a real world setting: cohort study in primary care. *BMJ (Clinical research ed* 2018;362:k2505. doi: 10.1136/bmj.k2505
2. Agnelli G, Buller HR, Cohen A, et al. Oral apixaban for the treatment of acute venous thromboembolism. *The New England journal of medicine* 2013;369(9):799-808. doi: 10.1056/NEJMoa1302507
3. Investigators E, Bauersachs R, Berkowitz SD, et al. Oral rivaroxaban for symptomatic venous thromboembolism. *The New England journal of medicine* 2010;363(26):2499-510. doi: 10.1056/NEJMoa1007903
4. Investigators E-P, Buller HR, Prins MH, et al. Oral rivaroxaban for the treatment of symptomatic pulmonary embolism. *The New England journal of medicine* 2012;366(14):1287-97. doi: 10.1056/NEJMoa1113572

For peer review only - http://bmjopen.bmj.com/site/about/guidelines.xhtml

5. Schulman S, Kearon C, Kakkar AK, et al. Dabigatran versus warfarin in the treatment of acute venous thromboembolism. *The New England journal of medicine* 2009;361(24):2342-52. doi: 10.1056/NEJMoa0906598
6. Lopez-Lopez JA, Sterne JAC, Thom HHZ, et al. Oral anticoagulants for prevention of stroke in atrial fibrillation: systematic review, network meta-analysis, and cost effectiveness analysis. *BMJ (Clinical research ed* 2017;359:j5058. doi: 10.1136/bmj.j5058
7. Protty MB, Hayes J. Dawn of the direct-acting oral anticoagulants: trends in oral anticoagulant prescribing in Wales 2009-2015. *Journal of clinical pharmacy and therapeutics* 2017;42(2):132-34. doi: 10.1111/jcpt.12481
8. Burnett A, Siegal D, Crowther M. Specific antidotes for bleeding associated with direct oral anticoagulants. *BMJ (Clinical research ed* 2017;357:j2216. doi: 10.1136/bmj.j2216
9. Mueller T, Alvarez-Madrazo S, Robertson C, et al. Comparative safety and effectiveness of direct oral anticoagulants in patients with atrial fibrillation in clinical practice in Scotland. *British journal of clinical pharmacology* 2019;85(2):422-31. doi: 10.1111/bcp.13814
10. Gunasekara FI, Richardson K, Carter K, et al. Fixed effects analysis of repeated measures data. *International journal of epidemiology* 2014;43(1):264-9. doi: 10.1093/ije/dyt221 [published Online First: 2013/12/25]
11. Rogers W. Regression standard errors in clustered samples. *Stata Technical Bulletin* 1994;3
12. Shadish WR, Cook TD, Thomas. CD. Experimental and Quasi-Experimental Designs for Generalised Causal Inference: Houghton Mifflin 2002.
13. Obamiro KO, Chalmers L, Bereznicki LR. A Summary of the Literature Evaluating Adherence and Persistence with Oral Anticoagulants in Atrial Fibrillation. *American journal of cardiovascular drugs : drugs, devices, and other interventions* 2016;16(5):349-63. doi: 10.1007/s40256-016-0171-6
14. Burn J, Pirmohamed M. Direct oral anticoagulants versus warfarin: is new always better than the old? *Open heart* 2018;5(1):e000712. doi: 10.1136/openhrt-2017-000712
15. Connolly SJ, Ezekowitz MD, Yusuf S, et al. Dabigatran versus warfarin in patients with atrial fibrillation. *The New England journal of medicine* 2009;361(12):1139-51. doi: 10.1056/NEJMoa0905561
16. Ezekowitz MD, Connolly S, Parekh A, et al. Rationale and design of RE-LY: randomized evaluation of long-term anticoagulant therapy, warfarin, compared with dabigatran. *American heart journal* 2009;157(5):805-10, 10 e1-2. doi: 10.1016/j.ahj.2009.02.005
17. Ogawa S, Shinohara Y, Kanmuri K. Safety and efficacy of the oral direct factor xa inhibitor apixaban in Japanese patients with non-valvular atrial fibrillation. -The ARISTOTLE-J study. *Circulation journal : official journal of the Japanese Circulation Society* 2011;75(8):1852-9.
18. Patel MR, Mahaffey KW, Garg J, et al. Rivaroxaban versus warfarin in nonvalvular atrial fibrillation. *The New England journal of medicine* 2011;365(10):883-91. doi: 10.1056/NEJMoa1009638
19. Ruff CT, Giugliano RP, Antman EM, et al. Evaluation of the novel factor Xa inhibitor edoxaban compared with warfarin in patients with atrial fibrillation: design and rationale for the Effective aNticoaGulation with factor xA next GEneration in Atrial Fibrillation-Thrombolysis In Myocardial Infarction study 48 (ENGAGE AF-TIMI 48). *American heart journal* 2010;160(4):635-41. doi: 10.1016/j.ahj.2010.06.042
20. Weitz JI, Connolly SJ, Patel I, et al. Randomised, parallel-group, multicentre, multinational phase 2 study comparing edoxaban, an oral factor Xa inhibitor, with warfarin for stroke prevention in patients with atrial fibrillation. *Thromb Haemost* 2010;104(3):633-41. doi: 10.1160/TH10-01-0066

For peer review only

21. Lutz J, Jurk K, Schinzel H. Direct oral anticoagulants in patients with chronic kidney disease: patient selection and special considerations. *International journal of nephrology and renovascular disease* 2017;10:135-43. doi: 10.2147/IJNRD.S105771
22. Buckley LF, Rybak E, Aldemerdash A, et al. Direct oral anticoagulants in patients with atrial fibrillation and renal impairment, extremes in weight, or advanced age. *Clinical cardiology* 2017;40(1):46-52. doi: 10.1002/clc.22591
23. Fanikos J, Burnett AE, Mahan CE, et al. Renal Function and Direct Oral Anticoagulant Treatment for Venous Thromboembolism. *The American journal of medicine* 2017;130(10):1137-43. doi: 10.1016/j.amjmed.2017.06.004
24. Fanikos J, Burnett AE, Mahan CE, et al. Renal Function Considerations for Stroke Prevention in Atrial Fibrillation. *The American journal of medicine* 2017;130(9):1015-23. doi: 10.1016/j.amjmed.2017.04.015
25. Zou R, Tao J, Shi W, et al. Meta-analysis of safety and efficacy for direct oral anticoagulation treatment of non-valvular atrial fibrillation in relation to renal function. *Thrombosis research* 2017;160:41-50. doi: 10.1016/j.thromres.2017.10.013
26. Wang KL, Chiu CC, Giugliano RP, et al. Drug Class, Renal Elimination, and Outcomes of Direct Oral Anticoagulants in Asian Patients: A Meta-Analysis. *J Stroke Cerebrovasc Dis* 2018;27(4):857-64. doi: 10.1016/j.jstrokecerebrovasdis.2017.10.027
27. Testa S, Tripodi A, Legnani C, et al. Plasma levels of direct oral anticoagulants in real life patients with atrial fibrillation: Results observed in four anticoagulation clinics. *Thrombosis research* 2016;137:178-83. doi: 10.1016/j.thromres.2015.12.001 [published Online First: 2015/12/18]
28. Inohara T, Xian Y, Liang L, et al. Association of Intracerebral Hemorrhage Among Patients Taking Non-Vitamin K Antagonist vs Vitamin K Antagonist Oral Anticoagulants With In-Hospital Mortality. *JAMA* 2018;319(5):463-73. doi: 10.1001/jama.2017.21917
29. Jun M, Lix LM, Durand M, et al. Comparative safety of direct oral anticoagulants and warfarin in venous thromboembolism: multicentre, population based, observational study. *BMJ (Clinical research ed* 2017;359:j4323. doi: 10.1136/bmj.j4323
30. Charlton B, Adeboyeje G, Barron JJ, et al. Length of hospitalization and mortality for bleeding during treatment with warfarin, dabigatran, or rivaroxaban. *PLoS One* 2018;13(3):e0193912. doi: 10.1371/journal.pone.0193912
31. The L. Personalised medicine in the UK. *Lancet* 2018;391(10115):e1. doi: 10.1016/S0140-6736(17)33261-0
32. Yang T, Zhou Y, Chen C, et al. Genotype-guided dosing versus conventional dosing of warfarin: A meta-analysis of 15 randomized controlled trials. *Journal of clinical pharmacy and therapeutics* 2018 doi: 10.1111/jcpt.12782
33. Jorgensen AL, Prince C, Fitzgerald G, et al. Implementation of genotype-guided dosing of warfarin with point-of-care genetic testing in three UK clinics: a matched cohort study. *BMC Med* 2019;17(1):76. doi: 10.1186/s12916-019-1308-7
34. Wool GD. Benefits and Pitfalls of Point-of-Care Coagulation Testing for Anticoagulation Management: An ACLPS Critical Review. *American journal of clinical pathology* 2019;151(1):1-17. doi: 10.1093/ajcp/aqy087
35. Kawai VK, Cunningham A, Vear SI, et al. Genotype and risk of major bleeding during warfarin treatment. *Pharmacogenomics* 2014;15(16):1973-83. doi: 10.2217/pgs.14.153
36. Pink J, Pirmohamed M, Lane S, et al. Cost-effectiveness of pharmacogenetics-guided warfarin therapy vs. alternative anticoagulation in atrial fibrillation. *Clin Pharmacol Ther* 2014;95(2):199-207. doi: 10.1038/clpt.2013.190
37. Gage BF, Bass AR, Lin H, et al. Effect of Genotype-Guided Warfarin Dosing on Clinical Events and Anticoagulation Control Among Patients Undergoing Hip or Knee

For peer review only - http://bmjopen.bmj.com/site/about/guidelines.xhtml

Arthroplasty: The GIFT Randomized Clinical Trial. *JAMA* 2017;318(12):1115-24. doi: 10.1001/jama.2017.11469
38. Pirmohamed M, Burnside G, Eriksson N, et al. A randomized trial of genotype-guided dosing of warfarin. *The New England journal of medicine* 2013;369(24):2294-303. doi: 10.1056/NEJMoa1311386
39. Green L, Tan J, Antoniou S, et al. Haematological management of major bleeding associated with direct oral anticoagulants - UK experience. *Br J Haematol* 2019 doi: 10.1111/bjh.15808
40. Green L, Tan J, Morris JK, et al. A three-year prospective study of the presentation and clinical outcomes of major bleeding episodes associated with oral anticoagulant use in the UK (ORANGE study). *Haematologica* 2018;103(4):738-45. doi: 10.3324/haematol.2017.182220

For peer review only

Figure legends

Figure 1. Trend in anticoagulant prescribing items and costs from GP practices in England and the proportion of direct oral anticoagulants of all anticoagulants prescribed. In 2014, NICE guidance on use of direct oral anticoagulants has changed.

Figure 2. Geographical pattern in DOAC prescribing as a proportion of all anticoagulant prescribing items in England 2011-2016 that includes changes in NICE guidance in 2014.

Figure 3 Estimates from regression model showing the relative change in the rate of emergency admissions for bleeding and clotting complications associated with each additional 10% of DOACs prescribed as a proportion of all anticoagulants.

Figure 4. Trend in emergency admissions for bleeding conditions between 2011 and 2016 and the trend, estimated from the regression model that would have been expected if DOACs had not been introduced.

For peer review only - http://bmjopen.bmj.com/site/about/guidelines.xhtml

For peer review only

For peer review only - http://bmjopen.bmj.com/site/about/guidelines.xhtml

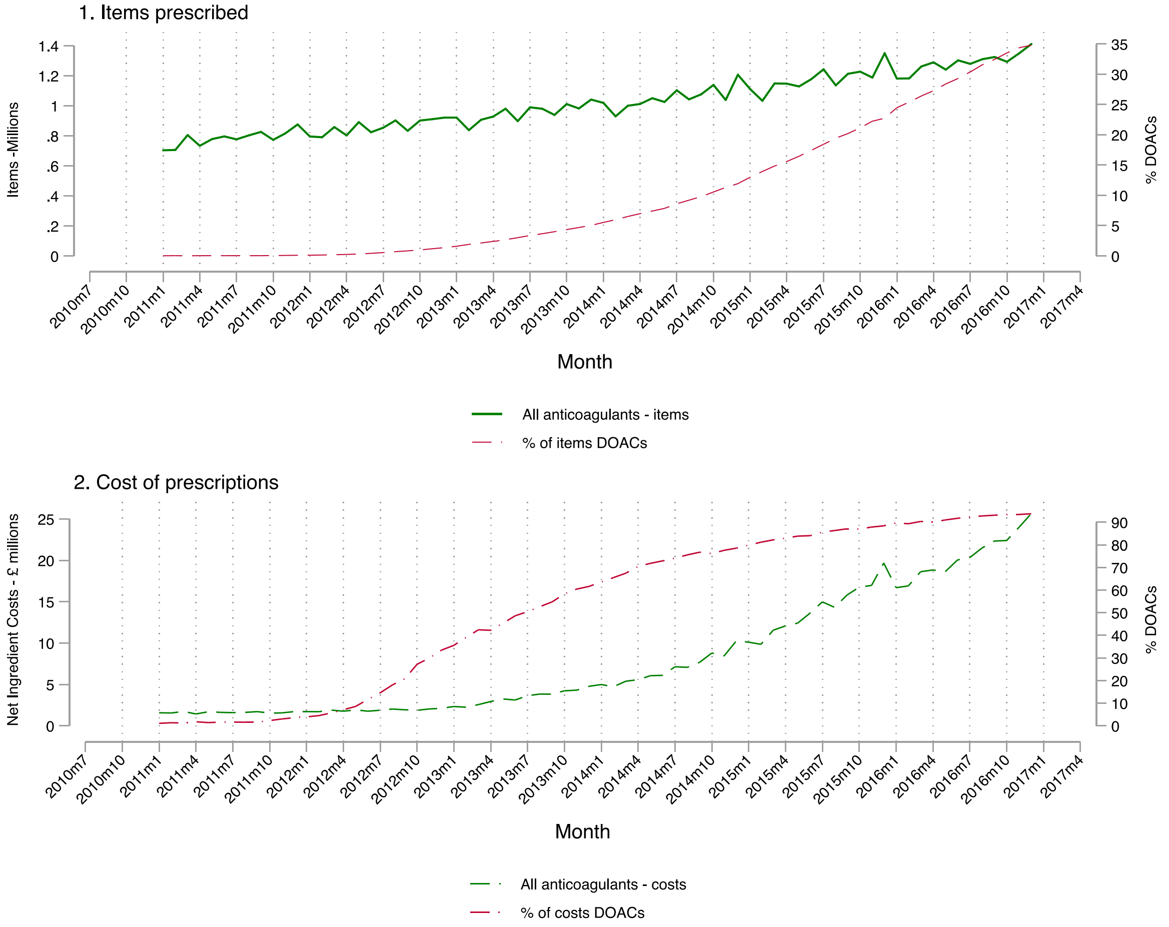
1. Items prescribed
Items -Millions
% DOACs
Month
All anticoagulants - items
% of items DOACs
2. Cost of prescriptions
Net Ingredient Costs - £ millions
% DOACs
Month
All anticoagulants - costs
% of costs DOACs

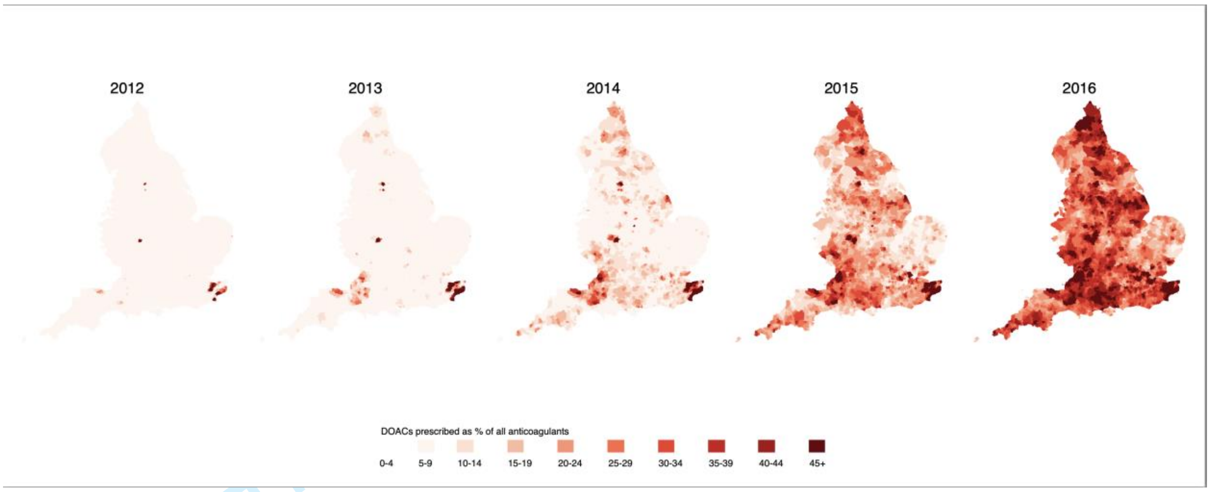

For peer review only

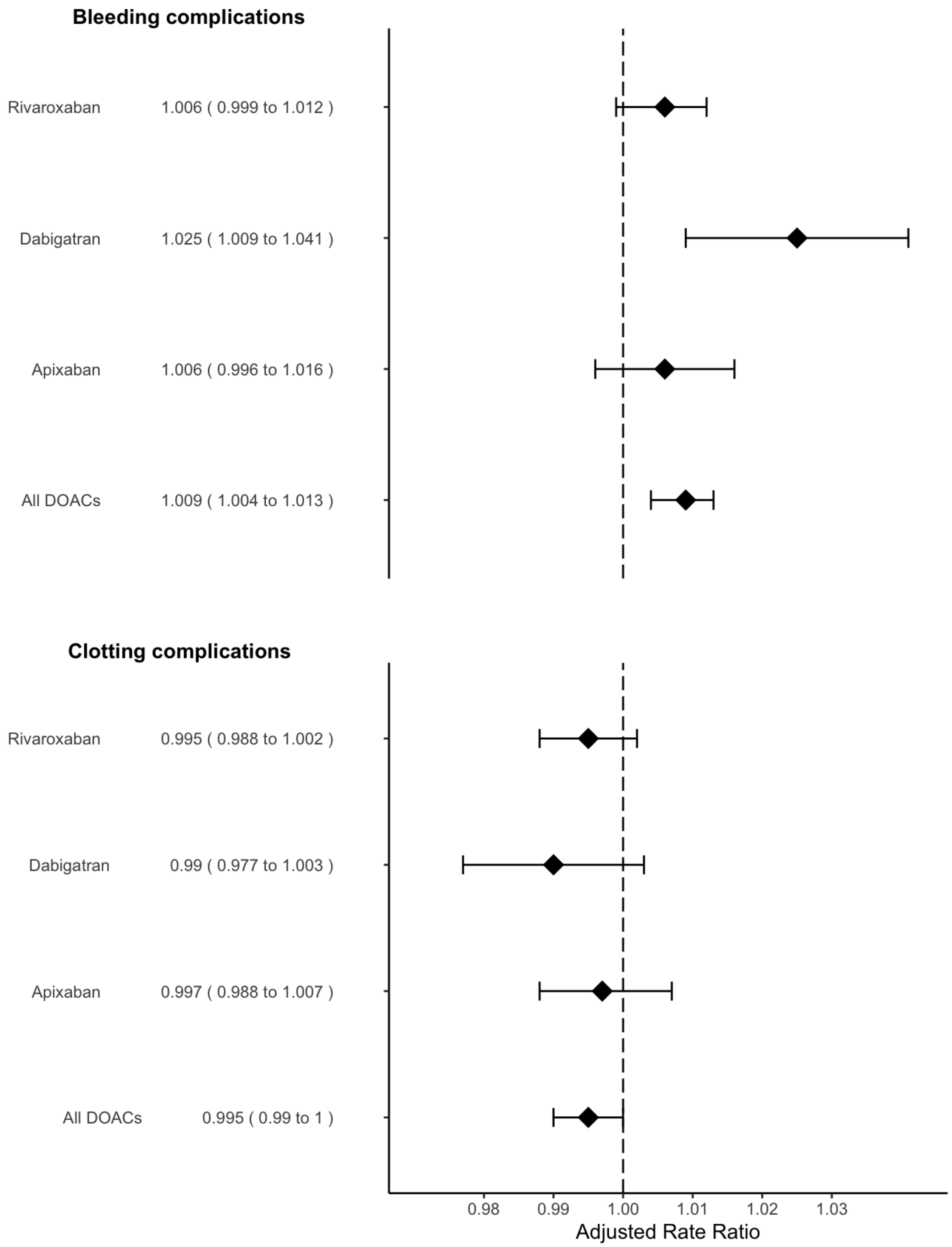
Bleeding complications
Rivaroxaban
1.006 (0.999 to 1.012)
Dabigatran
1.025 (1.009 to 1.041)
Apixaban
1.006 (0.996 to 1.016)
All DOACs
1.009 (1.004 to 1.013)
Clotting complications
Rivaroxaban
0.995 (0.988 to 1.002)
Dabigatran
0.99 (0.977 to 1.003)
Apixaban
0.997 (0.988 to 1.007)
All DOACs
0.995 (0.99 to 1)
0.98
0.99
1.00
1.01
1.02
1.03
Adjusted Rate Ratio

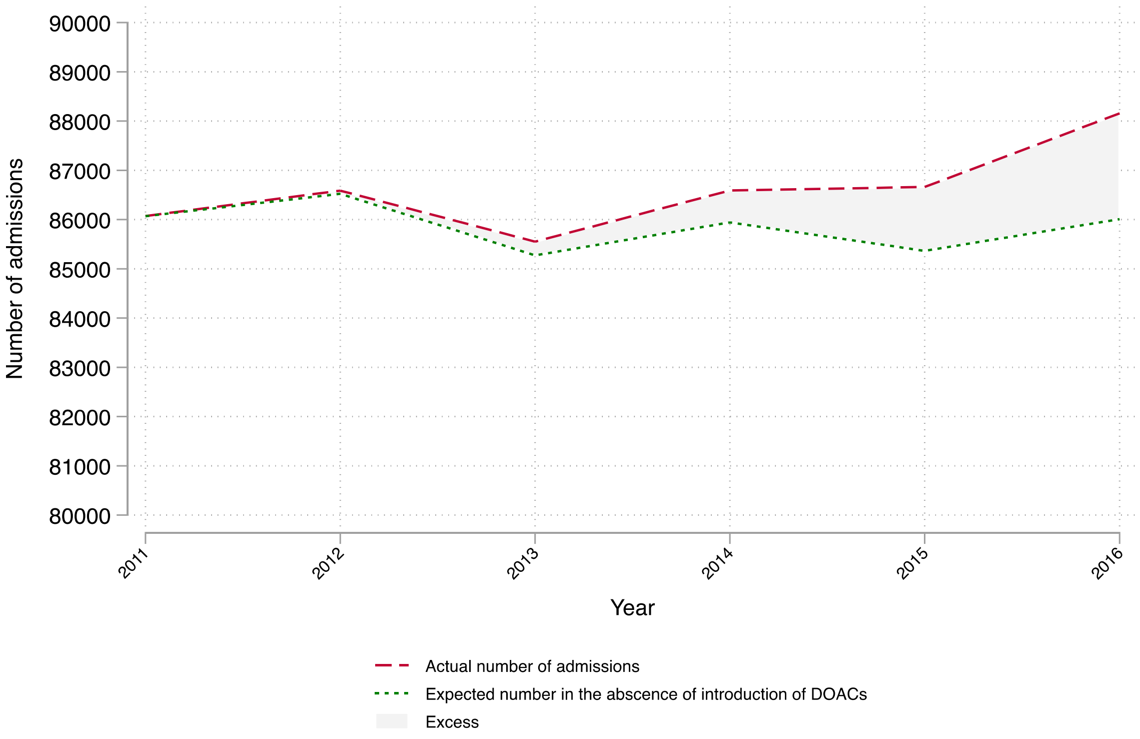
90000
89000
88000
87000
86000
85000
84000
83000
82000
81000
80000
Number of admissions
2011
2012
2013
2014
2015
2016
Year
Actual number of admissions
Expected number in the absence of introduction of DOACs
Excess

Appendix 1 BNF codes and ICD10 codes used to define exposure and outcome variables.

BNF codes used to define prescribing

0208020V - Warfarin

0208020X0 – Dabigatran

0208020X0 – edoxaban

0208020Y – rivaroxaban

0208020Z0 - Apixiban

ICD 10 Codes used to define outcomes

1. **Bleeding Complications**

ICD10 code	Name
M79.81	traumatic haematoma of the soft tissue
M25.00	Hemarthrosis, unspecified joint
K92	Other diseases of digestive system
K92.0	Haematemesis
K92.1	Melaena
K92.2	Gastrointestinal haemorrhage, unspecified
K62.5	Haemorrhage of anus and rectum
R58	Haemorrhage, not elsewhere classified
R04.0	Epistaxis
R04.1	Haemorrhage from throat
R04.2	Haemoptysis
R04.8	Haemorrhage from other sites in respiratory passages

For peer review only

R04.9	Haemorrhage from respiratory passages, unspecified
R31	Unspecified haematuria
N02	Recurrent or persistent haematuria
I60	Subarachnoid haemorrhage
I60.0	Subarachnoid haemorrhage from carotid siphon and bifurcation
I60.1	Subarachnoid haemorrhage from middle cerebral artery
I60.2	Subarachnoid haemorrhage from anterior communicating artery
I60.3	Subarachnoid haemorrhage from posterior communicating artery
I60.4	Subarachnoid haemorrhage from basilar artery
I60.5	Subarachnoid haemorrhage from vertebral artery
I60.6	Subarachnoid haemorrhage from other intracranial arteries
I60.7	Subarachnoid haemorrhage from intracranial artery, unspecified
I60.8	Other subarachnoid haemorrhage
I60.9	Subarachnoid haemorrhage, unspecified
I61	Intracerebral haemorrhage
I61.0	Intracerebral haemorrhage in hemisphere, subcortical
I61.1	Intracerebral haemorrhage in hemisphere, cortical
I61.2	Intracerebral haemorrhage in hemisphere, unspecified
I61.3	Intracerebral haemorrhage in brain stem
I61.4	Intracerebral haemorrhage in cerebellum
I61.5	Intracerebral haemorrhage, intraventricular
I61.6	Intracerebral haemorrhage, multiple localized
I61.8	Other intracerebral haemorrhage
I61.9	Intracerebral haemorrhage, unspecified
I62	Other nontraumatic intracranial haemorrhage
I62.0	Subdural haemorrhage (acute)(nontraumatic)
I62.1	Nontraumatic extradural haemorrhage

For peer review only

I62.9	Intracranial haemorrhage (nontraumatic), unspecified

2. Clotting Complications

**ICD10 code**	**Name**
I63	Cerebral infarction
I64	Stroke, not specified as haemorrhage or infarction
I26	Pulmonary embolism
I82	Other venous embolism and thrombosis
I80.0	Phlebitis and thrombophlebitis of superficial vessels of lower extremities
I80.1	Phlebitis and thrombophlebitis of femoral vein
I80.2	Deep vein thrombosis, Phlebitis and thrombophlebitis of other deep vessels of lower extremities
I80.3	Phlebitis and thrombophlebitis of lower extremities, unspecified
I80.8	Phlebitis and thrombophlebitis of other sites
I80.9	Phlebitis and thrombophlebitis of unspecified site

For peer review only

Appendix 2 flow outlining inclusion/ exclusion and missing data

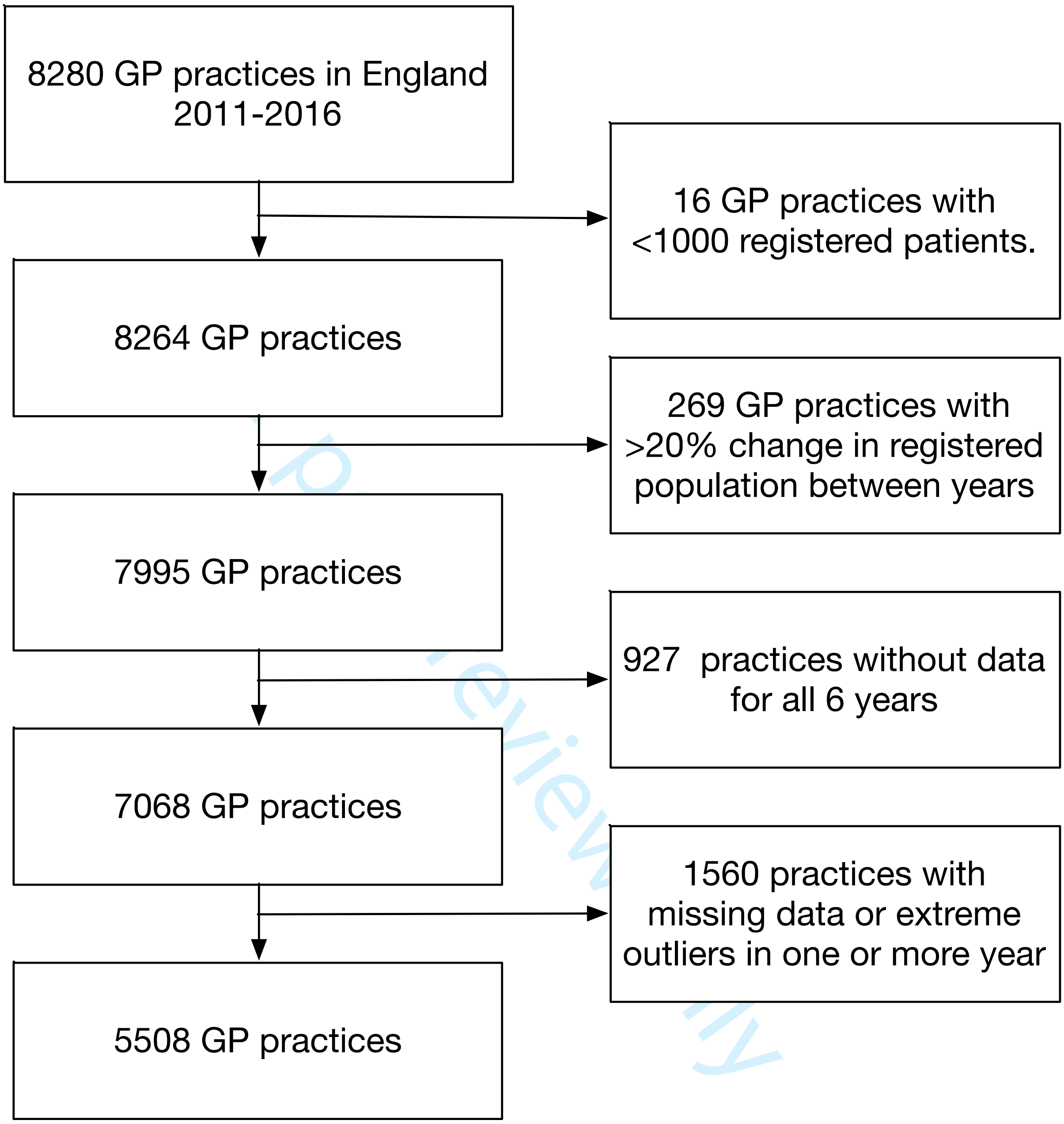

Appendix 3 alternative model specifications.

1. **Model using all GP practices – without applying exclusion criteria (8032 GP practices – 248 with missing data). Estimates from regression model showing the relative change in the rate of emergency admissions for bleeding and clotting complications associated with each additional 10% of DOACs prescribed as a proportion of all anticoagulants**

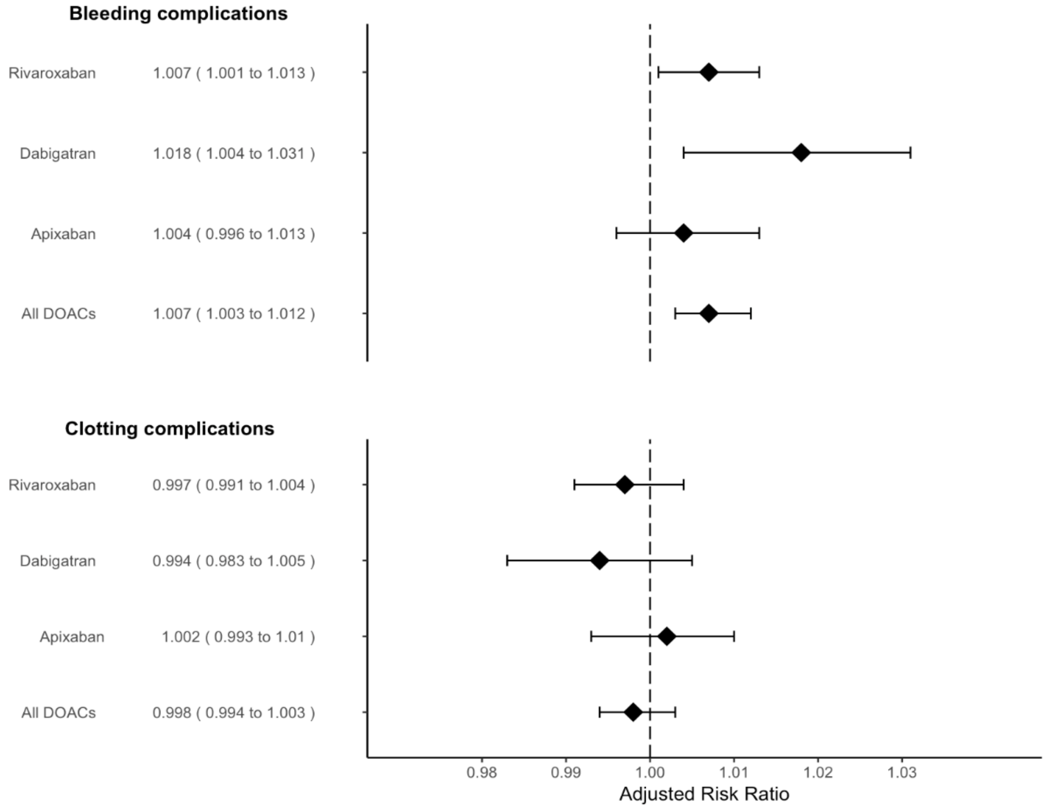

2. Negative Binomial Model

Estimates from regression model showing the relative change in the rate of emergency admissions for bleeding and clotting complications associated with each additional 10% of DOACs prescribed as a proportion of all anticoagulants

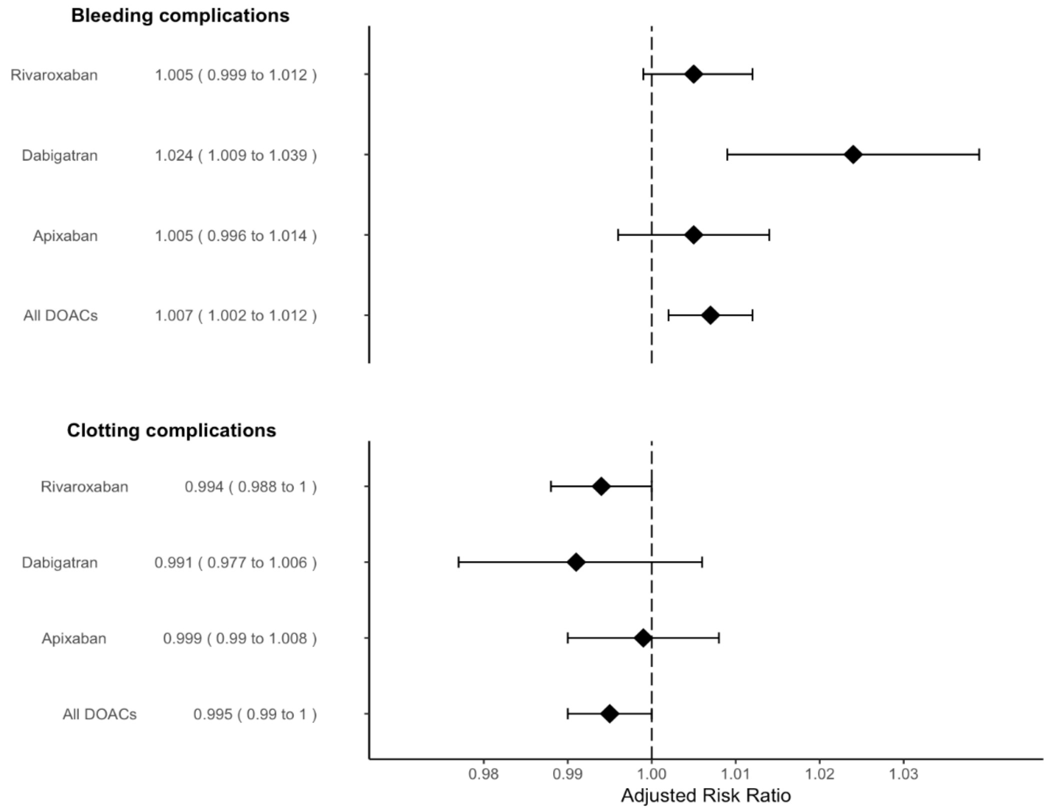

only

3. **Non-equivalent dependent variable.**

Estimates from regression model showing the relative change in the rate of emergency admissions for gastrointestinal admissions associated with each additional 10% of DOACs prescribed as a proportion of all anticoagulants.

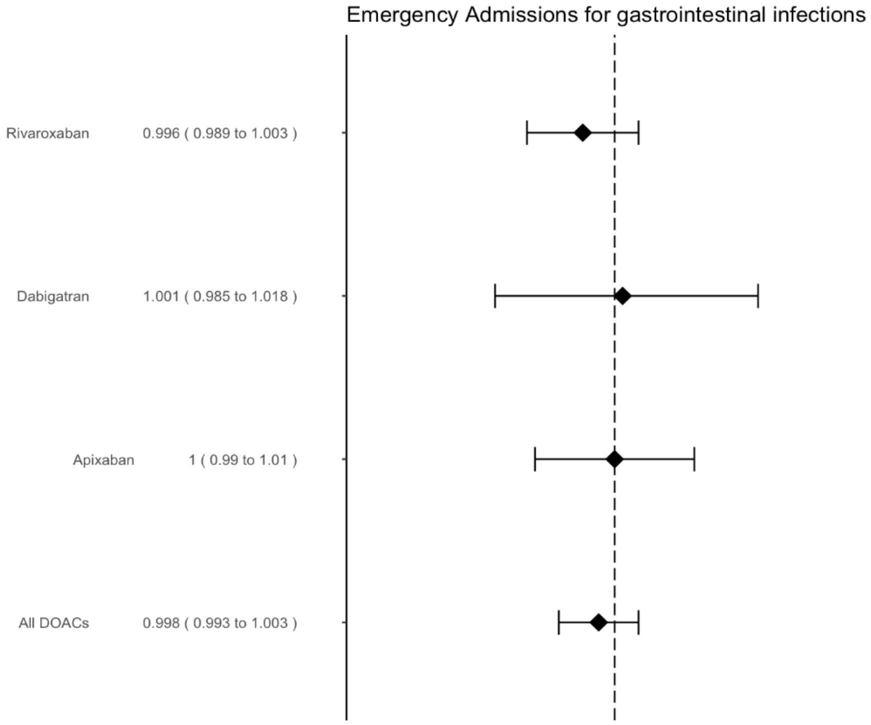

4. General Estimating equation - with exchangeable correlation structure.

Estimates from regression model showing the relative change in the rate of emergency admissions for bleeding and clotting complications associated with each additional 10% of DOACs prescribed as a proportion of all anticoagulants

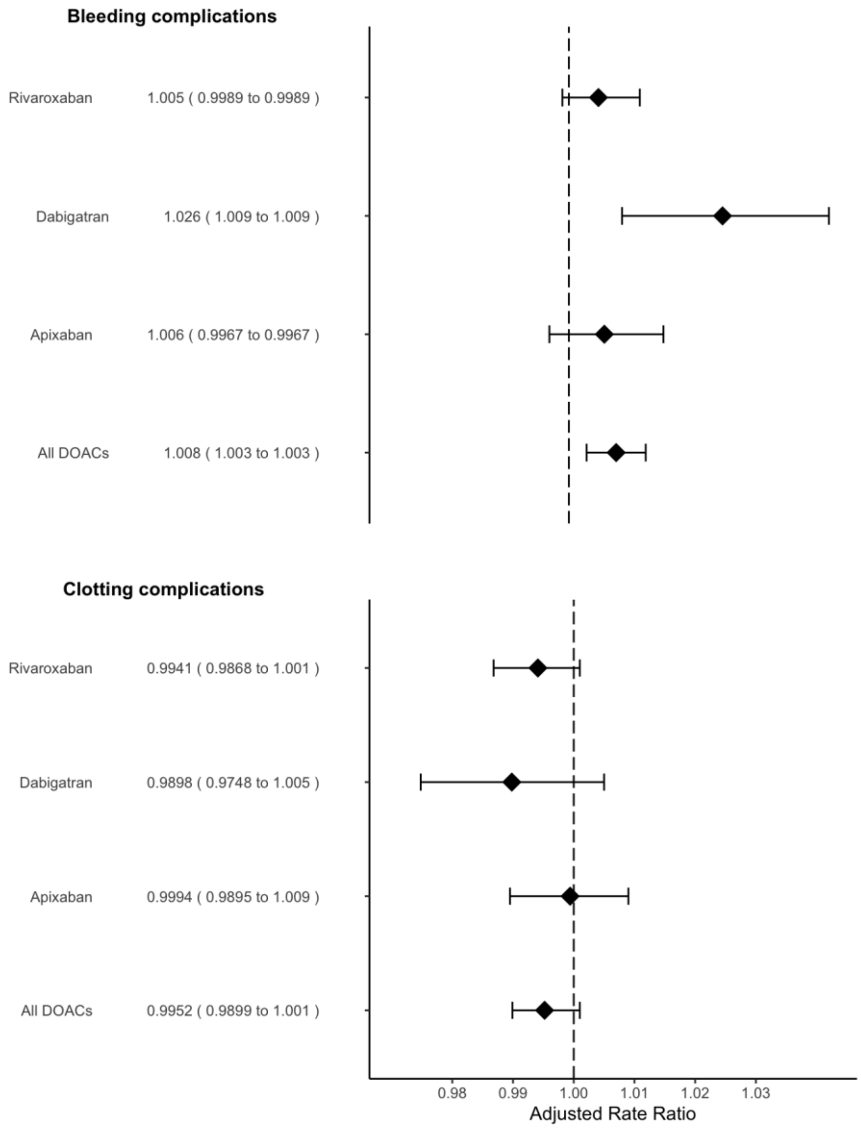

5. General Estimating equation - autoregressive(AR-1) correlation structure. Estimates from regression model showing the relative change in the rate of emergency admissions for bleeding and clotting complications associated with each additional 10% of DOACs prescribed as a proportion of all anticoagulants

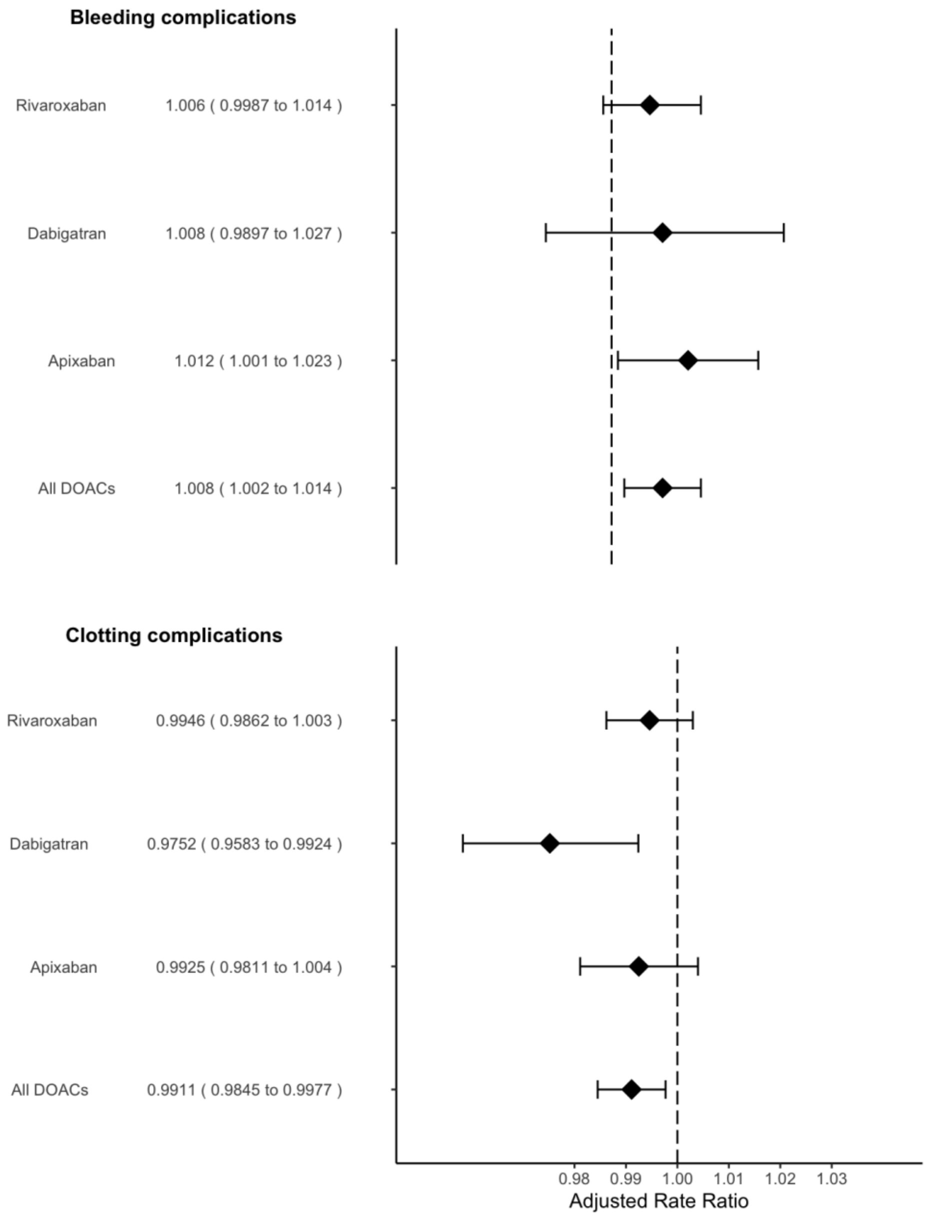

STROBE Statement—checklist of items that should be included in reports of observational studies
Paper submission:
Alfirevic et al. Has the introduction of direct oral anticoagulants (DOACs) in England increased emergency admissions for bleeding conditions? A longitudinal ecological study.

	Item No	Recommendation
**Title and abstract**	1	(*a*) We've indicated the study's design in the title or the abstract
		(*b*) We've provided in the abstract an informative and balanced summary of what was done and what was found, page 2
**Introduction**		
Background/rationale	2	We've explained the scientific background and rationale for the investigation being reported, pages 3 and 4
Objectives	3	State specific objectives, including any prespecified hypotheses- done, page 4, line 12 to 18
**Methods**		
Study design	4	Key elements of study design were presented on page 5, lines 12 to 27
Setting	5	The setting, locations, and relevant dates, including periods of recruitment, exposure, follow-up, and data collection were presented on pages 4 and 5
Participants	6	We included all GP practices in England with a registered population size greater than 1000, which had data for all 6 years (N=5508 GP practices with 33,048 practice years of data. Page 5
		Inclusions/exclusions and missing data are outlined in Supplementary material- Appendix 2
Variables	7	Clearly defined outcomes (BNF and ICD10 codes) are presented in Supplementary material, Appendix 1
Data sources/ measurement	8*	Sources of data and details of methods of assessment are presented on pages 4 and 5. Describe comparability of assessment methods if there is more than one group
Bias	9	Potential sources of bias such as confounding from unobserved factors that vary between GP practice populations, we used a fixed effects approach and a dummy variable for each year to account for the national trend in prescribing and emergency admissions. In addition, we performed sensitivity analyses
Study size	10	All eligible GP practices in England were included in this study
Quantitative variables	11	We've explained how quantitative variables were handled in the analyses on pages 5 and 6
Statistical methods	12	(*a*) Describe all statistical methods, including those used to control for confounding- done, page 5 and 6
		(*b*) Describe any methods used to examine subgroups and interactions, done page 5 and 6
		(*c*) Explain how missing data were addressed- done flow chart Supplementary material Appendix 2
		(*e*) Sensitivity analyses are described on page 6

Continued on next page

For peer review only - http://bmjopen.bmj.com/site/about/guidelines.xhtml

For peer review only

**Results**		
Participants	13*	(a) Report numbers of individuals at each stage of study-done, Supplementary material Appendix 2
		(b) Give reasons for non-participation at each stage- done Appendix 2
		(c) Flow diagram- done and shown in Appendix 2
Descriptive data	14*	(a) Give characteristics of study participants – GP practice data included, no data on individual participants are available
		(b) We've indicated the number of practices with missing data in Appendix 2
		(c) *F*ollow-up time of 6 years has been clearly shown in the manuscript (page 5 and 7) and Figures 1 and 2
Outcome data	15*	
		Outcome data are described in the manuscript, page 7
Main results	16	(*a*) We have given unadjusted estimates and their precision (95% confidence interval), page 7
		(*c*) We haven't translated estimates of relative risk into absolute risk
Other analyses	17	We have reported sensitivity analyses, page 7, lines 50-59
**Discussion**		
Key results	18	Summarise key results with reference to study objectives- done, page 8
Limitations	19	Discuss limitations of the study, taking into account sources of potential bias or imprecision. Discuss both direction and magnitude of any potential bias- done page 9 line 22
Interpretation	20	Give a cautious overall interpretation of results considering objectives, limitations, multiplicity of analyses, results from similar studies, and other relevant evidence done page 10
Generalisability	21	Discuss the generalisability (external validity) of the study results – we have compared our results with previous studies, page 10
**Other information**		
Funding	22	Give the source of funding and the role of the funders for the present study and, if applicable, for the original study on which the present article is based- Done, we have included a sentence at the end of the manuscript to acknowledge our NIHR CLAHRC NWC funding

*Give information separately for cases and controls in case-control studies and, if applicable, for exposed and unexposed groups in cohort and cross-sectional studies.

Note: An Explanation and Elaboration article discusses each checklist item and gives methodological background and published examples of transparent reporting. The STROBE checklist is best used in conjunction with this article (freely available on the Web sites of PLoS Medicine at http://www.plosmedicine.org/, Annals of Internal Medicine at http://www.annals.org/, and Epidemiology at http://www.epidem.com/). Information on the STROBE Initiative is available at www.strobe-statement.org.

For peer review only

BMJ Open

Has the introduction of direct oral anticoagulants (DOACs) in England increased emergency admissions for bleeding conditions? A longitudinal ecological study.

Journal:	*BMJ Open*
Manuscript ID	bmjopen-2019-033357.R2
Article Type:	Original research
Date Submitted by the Author:	07-Jan-2020
Complete List of Authors:	Alfirevic, Ana; University of Liverpool, Molecular and Clinical Pharmacology<br>Downing, Jennifer; University of Liverpool, Molecular and Clinical Pharmacology<br>Daras, Konstantinos ; University of Liverpool School of Environmental Sciences, Geography and Planning<br>Comerford, Terence; University of Liverpool<br>Pirmohamed, Munir; University of Liverpool, Molecular and Clinical Pharmacology<br>Barr, Ben
**Primary Subject Heading**:	Public health
Secondary Subject Heading:	Pharmacology and therapeutics
Keywords:	CLINICAL PHARMACOLOGY, PRIMARY CARE, PUBLIC HEALTH

SCHOLARONE™
Manuscripts

I, the Submitting Author has the right to grant and does grant on behalf of all authors of the Work (as defined in the below author licence), an exclusive licence and/or a non-exclusive licence for contributions from authors who are: i) UK Crown employees; ii) where BMJ has agreed a CC-BY licence shall apply, and/or iii) in accordance with the terms applicable for US Federal Government officers or employees acting as part of their official duties; on a worldwide, perpetual, irrevocable, royalty-free basis to BMJ Publishing Group Ltd ("BMJ") its licensees and where the relevant Journal is co-owned by BMJ to the co-owners of the Journal, to publish the Work in this journal and any other BMJ products and to exploit all rights, as set out in our licence.

The Submitting Author accepts and understands that any supply made under these terms is made by BMJ to the Submitting Author unless you are acting as an employee on behalf of your employer or a postgraduate student of an affiliated institution which is paying any applicable article publishing charge ("APC") for Open Access articles. Where the Submitting Author wishes to make the Work available on an Open Access basis (and intends to pay the relevant APC), the terms of reuse of such Open Access shall be governed by a Creative Commons licence – details of these licences and which Creative Commons licence will apply to this Work are set out in our licence referred to above.

Other than as permitted in any relevant BMJ Author's Self Archiving Policies, I confirm this Work has not been accepted for publication elsewhere, is not being considered for publication elsewhere and does not duplicate material already published. I confirm all authors consent to publication of this Work and authorise the granting of this licence.

For peer review only

Has the introduction of direct oral anticoagulants (DOACs) in England increased emergency admissions for bleeding conditions? A longitudinal ecological study.

Ana Alfirevic[1], Jennifer Downing[1], Konstantinos Daras[2], Terence Comerford[3], Munir Pirmohamed[1] and Ben Barr[2]

[1] The Wolfson Centre for Personalised Medicine, Department of Molecular and Clinical Pharmacology, Institute of Translational Medicine, University of Liverpool, UK

[2] Department of Public Health and Policy, Institute of Psychology, Health and Society, University of Liverpool, UK

[3] CLAHRC NWC Public Advisor Group, University of Liverpool, UK

Corresponding Author:

Ana Alfirevic

The Wolfson Centre for Personalised Medicine,

Department of Molecular and Clinical Pharmacology,

Institute of Translational Medicine

University of Liverpool

Block A, The Waterhouse Building

1-5 Brownlow Street

Liverpool, L69 3GL

Tel: +44 (0)151 7945551

E-mail: Ana.Alfirevic@liv.ac.uk

For peer review only - http://bmjopen.bmj.com/site/about/guidelines.xhtml

ABSTRACT

Objective. There is concern about long term safety of direct oral coagulants (DOACs) in clinical practice. Our aim was to investigate whether the introduction of DOACs compared with vitamin-K antagonists in England was associated with a change in admissions for bleeding or thromboembolic complications.

Setting: 5508 General Practitioner (GP) practices in England between 2011 and 2016.

Participants: All GP practices in England with a registered population size of greater than 1000 that had data for all 6 years.

Main outcome measure: The rate of emergency admissions to hospital for bleeding or thromboembolism, per 100,000 population for each GP practice in England.

Main exposure measure: The annual number of DOAC items prescribed for each GP practice population as a proportion of all anticoagulant items prescribed.

Design. This longitudinal ecological study used panel regression models to investigate the association between trends in DOAC prescribing within GP practice populations and trends in emergency admission rates for bleeding and thromboembolic conditions, whilst controlling for confounders.

Results. For each additional 10% of DOACs prescribed as a proportion of all anticoagulants, there was a 0.9% increase in bleeding complications (rate ratio (RR) 1.009 95% confidence intervals (CI) 1.004 to 1.013). The introduction of DOACs between 2011 and 2016 was associated with additional 4929 (95%CI 2489 to 7370) emergency admissions for bleeding complications. Increased DOAC prescribing was associated with a slight decline in admission for thromboembolic conditions.

Conclusion. Our data show that the rapid increase in prescribing of DOACs after changes in the NICE guidance in 2014 may have been associated with a higher rate of emergency admissions for bleeding conditions. These consequences need to be considered in assessing the benefits and costs of the widespread use of DOACs.

Word count 282

Key words Direct oral anticoagulants, warfarin, emergency admissions, bleeding, thromboembolism

For peer review only

Article Summary

Strengths and limitations of this study

- The majority of GP practices in England were included in our analyses and therefore we could estimate effects across the whole population
- Longitudinal data analyses included the changes in the guidelines for prescribing anticoagulants
- We used effectiveness and safety data on anticoagulants used in England
- It was not possible to link information on the prescribing for specific individuals to particular hospital admissions due to the ecological study design
- No control for adherence to medications was possible

For peer review only

Introduction

Prescribing of direct oral anticoagulants (DOACs) such as dabigatran, rivaroxaban, apixaban and edoxaban that were licenced between 2013 and 2015, has increased rapidly in the UK. DOACs are used for stroke prevention in atrial fibrillation (AF), treatment of venous thromboembolism (VTE), and medical and surgical thromboprophylaxis.

Warfarin has been the primary oral anticoagulant in patients with AF and VTE for over 60 years. Its use is associated with large inter-individual variability in dose requirements, narrow therapeutic index, slow onset of action, low time in therapeutic range and concomitant drug and food interactions. In addition, regular International Normalised Ratio (INR) monitoring required for optimising loading and maintenance doses is burdensome to the patients and healthcare professionals. The DOACs are recommended by NICE guidelines for the treatment of adults with venous thromboembolism, prevention of recurrent deep vein thrombosis, and stroke prevention in patients with non-valvular atrial fibrillation[1]. Several non-inferiority randomised controlled trials have demonstrated that DOACs have comparable efficacy and safety profiles with warfarin [2-5]. In addition, in clinical trials fixed-dose administration was proposed for DOACs, without the need for routine laboratory monitoring. This has led to widespread uptake of DOACs into clinical practice. Although DOACs have been shown to be cost-effective in RCTs[6], their high cost compared with the cost of warfarin (inclusive of INR testing) has major budget implications for the NHS in the UK and other healthcare services globally[7]. Concern about the escalating costs to healthcare is increasing not only for DOACs, but also when complications occur, for their reversal agents such as idarucizumab or andexanet alpha[8].

Data on the comparative safety and efficacy of DOACs in real-life observational studies has also become available more recently[6]. For instance, a recent large observational study in the UK found that apixaban was associated with decreased risk of bleeding complications, but rivaroxaban and low dose apixaban were associated with an increased risk of all-cause mortality compared to warfarin[1]. A large study (>14,500 patients) conducted in Scotland demonstrated that patients taking rivaroxaban may be

For peer review only - http://bmjopen.bmj.com/site/about/guidelines.xhtml

at increased risk of bleeding compared with other DOACs[9]. Recent concern about bleeding risks associated with DOACs has prompted the EMA to launch a safety review (https://www.ema.europa.eu/documents/other/direct-oral-anticoagulants-doacs-ema-starts-review-study-bleeding-risk-direct-oral-anticoagulants_en.pdf).

In this study we determined whether differences in prescribing of DOACs compared to warfarin between General Practitioner (GP) practices across England has been associated with variation of trends in emergency hospital admissions for bleeding and thromboembolic events from GP practice registered populations.

Methods

Setting and data sources.

We identified DOACs as dabigatran etexilate, apixaban, edoxaban or rivaroxaban using the BNF codes given in Appendix 1. We used the following NHS Digital data:

1) Practice level prescribing data that are published and made available by the NHS Business Service Authority (BSA) each month. We used the Practice Prescribing Data File with the full 15-digit BNF code to calculate the annual DOAC prescribing rate as the percentage of DOAC items prescribed in proportion to all anticoagulants items prescribed for each GP practice in England between 2011 and 2016. The prescribing rate for each separate DOAC (dabigatran etexilate, apixaban, edoxaban or rivaroxaban) was calculated as the percentage of items of these medications prescribed as a proportion of all anticoagulants items prescribed. A prescription item refers to a single item prescribed on a prescription form, generally a course of medicine and is routinely used to measure trends in prescribing. We used the Net Ingredient Costs of these items included in this data set to calculate the prescribing costs.

2) Hospital Episode Statistics data were used to calculate the annual emergency admission rate for bleeding and clotting conditions for each GP practice. The ICD10 diagnostic codes included in this

For peer review only - http://bmjopen.bmj.com/site/about/guidelines.xhtml

indicator is given in Appendix 1. Rates per 100,000 were calculated using annual data on the number of people registered with each GP practice provided by NHS Digital.

To additionally control for trends in the population age profile and underlying trends in morbidity, we also calculated the annual proportion of a GP population that was over 75 and used annual data on the prevalence of Atrial Fibrillation (AF), Coronary Heart Disease (CHD) and Chronic Kidney Disease (CKD) for each GP practice population using data reported through the Quality and Outcome Framework (QOF) returns.

We included all GP practices in England with a registered population size of greater than 1000 that had data for all 6 years. We excluded GP practices with values for prevalence estimates from QOF that were clear outliers (more than 2 interquartile ranges (IQRs) below the first quartile or above the third quartile) as these probably represent reporting errors. In addition, we excluded practices where the practice registered population has changed by more than 20% between consecutive years as this would have reflected a large change in the practice population probable due to practice mergers or closures. This provided 5508 practices for the final analysis, each providing 6 years of data – i.e. 33,048 practice years of data. A flow chart is given in Appendix 2 detailing exclusions.

Analyses

Initially, we investigated geographical pattern of increases in DOACs prescribing across England. We mapped GP level prescribing data to Lower Super Output Areas (LSOA), based on the proportion of each GP practice's population that lived in each LSOA. Next, we plotted national maps for the DOAC prescribing rate each year. We then used a fixed effects Poisson regression model to investigate the association between the trend in prescribing within a GP practice and the trend in the rate of admissions for bleeding conditions. The outcome measure was the number of admissions and the log of the GP registered population was used as an offset. As there is potential confounding from unobserved factors that vary between GP practice populations, we used a fixed effects approach to remove these between-GP practice differences[10]. This conservative approach is the equivalent to including dummy variables

For peer review only - http://bmjopen.bmj.com/site/about/guidelines.xhtml

for each GP practice so that the model assesses the association between the trend in prescribing and the trend in hospital admissions within each GP practice. We additionally included a dummy variable for each year to account for the national trend in prescribing and emergency admissions. To additionally control for differential trends in need for anticoagulants and risk of bleeding complications in each GP population, we included annual measures of the prevalence of AF, CHD and CKD and the proportion of the GP population over 75-year olds. We used Huber-White clustered standard errors that are robust to divergence of the data from the assumptions of a Poisson distribution and the clustering of variance between GP practices[11]. To investigate whether there were different effects associated with different types of DOACs, we repeated the analysis for dabigatran, apixaban and rivaroxoban. As edoxaban was only licenced in the middle of 2015, there was insufficient data to analyse this separately. We present the effect estimates (Rate Ratios) per additional 10% of DOACs prescribed, rather than for each additional 1 percentage point increase as this reflects a more meaningful level of increase than a 1 percentage point increase. This is achieved by dividing the rate ratio by 10 to give the effect per 10 percentage points of DOAC prescribing.

Ethical approval was not required for this ecological study data analyses.

Sensitivity analysis

We subjected our analysis to a number of tests to assess the robustness of our findings. We estimated a negative binomial model as opposed to a Poisson model which is more robust to over-dispersion in the data, estimated General Estimating Equations with exchangeable and autoregressive correlation structures and replicated our models using data from all GP practices (i.e. not applying any exclusion). To test the specificity of results, we estimated our model using a gastro-intestinal infection as a Nonequivalent Dependent Variables (NDV)[12]. This outcome should *not* be influenced by a change in the exposure but could be influenced by unobserved confounding factors that influence general trends in hospitalization in a GP practice population.

Patient involvement

No patients were involved in setting the research question or the outcome measures, nor were they involved in developing plans for design or implementation of the study. Patients and patient

For peer review only - http://bmjopen.bmj.com/site/about/guidelines.xhtml

representatives within the NIHR CLAHRC NWC were asked to revise the manuscript in particular the lay language summary. The results of the study are going to be disseminated to the relevant patient and public groups through the CLAHRC NWC communication programme.

Results

Figure 1 shows the trend in the number of items and net drug costs of DOACs prescribed in England by GP practices between 2011 and 2016. Between 2011 and 2016, the number of items of anticoagulants prescribed had risen from 0.7 million to 1.4 million per month and the proportion of these that were DOACs had risen from <1% to 35%. Costs of anticoagulants rose more steeply, from £1.6 million per month in 2011 to £26 million per month by the end of 2016. This rise in costs was due to increased prescribing of DOACs, which by the end of 2016 accounted for 94% of the cost of all anticoagulant prescribing.

Figure 2 shows the geographical pattern of DOAC prescribing each year. The increase in uptake of DOACs has not been consistent across the country, with the prescribing rate increasing markedly more in some part of the country compared to others. In particular, areas around Somerset, Kent, and to a lesser extent the North East, appeared to have adopted DOACs sooner than other areas. By 2016 there was markedly lower usage of DOACs in the North West and East Anglia.

The results of the regression model indicate that differences in the trends in DOAC prescribing between GP practices from 2011 to 2016 were associated with differences in the trends in admissions for bleeding conditions. For each additional 10% of DOACs prescribed as a proportion of all anticoagulants, there was a 0.9% increase in emergency admissions for bleeding complications (RR:1.009 95% CI 1.004to 1.013). We present the effect estimates per additional 10% of DOACs prescribed as this reflects a more meaningful level of increase than a 1 percentage point increase. In other words for each additional 1% of DOACs prescribed as a proportion of all anticoagulants, there was a 0.07% increase in emergency admissions for bleeding complications (RR:1.0007 95% CI 1.0002 to 1.0013). To give the effect per 10% of DOACs prescribed presented above we have just divided the

For peer review only - http://bmjopen.bmj.com/site/about/guidelines.xhtml

results for 1 percent change by 10. Increased risk of emergency admissions for bleeding complications was particularly associated with increased use of dabigatran. For each additional 10% of DOACs prescribed as a proportion of all anticoagulants, there was a 0.5% reduction in emergency admissions for clotting complications (RR:0.995 95% CI 0.99 to 1.0004, p=0.065) see Figure 3. The p-value of 0.065 indicates that there was a relatively high probability (6.5%) that this result could have occurred even if there was no true association between DOAC prescribing and emergency admissions for clotting complications.

Figure 4 shows the trend in emergency admissions for bleeding conditions between 2011 and 2016 and the trend, estimated from the regression model that would have been expected if DOACs had not been introduced. Overall the introduction of DOACs between 2011 and 2016 was associated with 1.94 additional emergency admissions for bleeding complications per 100,000 people per year [0.98 to 2.9], the equivalent to a total of 4929 emergency admissions for bleeding complications (95% CI 2489 to 7370).

Our results were similar when using alternative model specifications (see Appendix 3). We found similar results when using a negative binomial model as opposed to a Poisson regression, when using General Estimating Equations with alternative correlation structures and when replicating our model using data from all GP practices (i.e. not applying any exclusions). We found no association between the trend in DOAC prescribing and emergency admissions when applying the model with a non-equivalent dependant variable (gastro-intestinal infections).

Discussion

In this study, we demonstrated a sharp increase in the uptake of DOACs by general practitioners in the UK between 2011 and 2016, compared with the current standard, warfarin. The uptake of DOACs has not been consistent and there are geographical areas with markedly higher rate of DOAC prescribing. Worryingly, higher rates of DOAC prescribing were associated with higher rates of hospital admissions for bleeding events, with a slight decline in emergency admissions for thromboembolic complications.

For peer review only

For peer review only - http://bmjopen.bmj.com/site/about/guidelines.xhtml

We projected that with every 10% increase of DOAC prescribing as a proportion of all anticoagulants, there was a 0.9% increase in emergency admissions for bleeding complications. Moreover, we estimated from our regression model that between 2011 and 2016, there were an additional 4929 emergency admissions for bleeding complications, more than would have been expected if DOACs had not been introduced. At the end of 2016, 94% of the total expenditure on anticoagulants prescribed by GP practices in England was spent on DOACs, which represented only 35% of all prescribed anticoagulants.

The strengths and limitations of the study

The strength of this study is that we analysed data over several years that included the changes in the guidelines for prescribing anticoagulants. In 2014, the UK National Institute for Health and Care Excellence (NICE) guidance recommended the use of DOACs and warfarin taking into consideration clinician and patient preferences. Comparable efficacy and the evidence from RCTs of better safety of DOACs compared with warfarin, combined with no need for regular monitoring and a wider therapeutic range, resulted in a major increase in prescribing[1 3 4 6]. We captured this increase in our longitudinal analyses. Our sample includes the majority of GP practices in England and is therefore able to estimate effects across the whole population. Our model assessed the association between the trend in prescribing and the trend in hospital admissions within each GP practice and we included a dummy variable for each year to account for the national trends. Any bias in our model of the association between our main exposure variable and outcome is therefore unlikely to be the result of time-invariant confounders that differ between GP practice population, or from any changes over time that effect all GP practices equally. We found a similar effect when using alternative models. When replicating the analysis with a common emergency admission we would not expect to be affected by DOAC prescribing such as gastrointestinal infections, we found, as expected, no association. Additional strength of our study is that we included in our analyses both, effectiveness and safety data on anticoagulants used in England. We investigated whether increased bleeding risk has been compensated by a reduction in risk from clotting conditions and found that although estimates of Rate Ratios were less than 1, confidence intervals crossed 1, indicating that this result could have occurred even if there

For peer review only - http://bmjopen.bmj.com/site/about/guidelines.xhtml

was no true association between DOAC prescribing and emergency admissions for clotting complications.

A number of limitations remain. Firstly, the ecological design meant that it was not possible to link information on the prescribing for specific individuals to particular hospital admissions. Therefore, the associations observed at the aggregate level may not reflect associations at the level of the individual patients. Secondly, whilst our analysis adjusts for a number of observed and unobserved confounders, it is still possible that there are unobserved trends in factors that increase DOAC prescribing whilst also increasing risk of bleeding complications. Bias could be introduced for example if there are differential trends between GP practices in underlying risk of bleeding complications, that are not accounted for by our control variables and this increased risk of bleeding complications led to increased DOAC prescribing in these practices. As our outcomes were measured at the population level our study will, however, be less at risk of selection bias, than studies using individual follow up, where this results from clinical decisions to preferentially prescribe DOACs to patients with higher risk of bleeding complications. Thirdly, we could not control for adherence to medications in our study. Adherence to anticoagulants has been reviewed recently[13] with some contradictory reports. Although it is expected that adherence would be better for DOACs because of no need for regular monitoring, regular INR tests required to ensure the correct dose of warfarin lead to stringent check-ups for adherence, while DOACs monitoring is not recommended and therefore adherence declines after the initial period[14]. With long term adherence monitoring our estimates would have been more precise.

Comparisons with previous studies

Our results differ from the findings of several large-scale non-inferiority RCTs that have demonstrated better bleeding profiles of DOACs over warfarin[5 15-20]. This may not be surprising as trial data may provide limited information on relatively rare adverse effects due to low power and short follow up. In addition, participants in clinical trials may be younger with a fewer comorbidities. For example, renal impairment that occurs in the older population may be relevant. Renal clearance is more dominant for DOACs compared with warfarin, as 80%, 50%, 36% and 27% of unchanged dabigatran, edoxaban,

For peer review only - http://bmjopen.bmj.com/site/about/guidelines.xhtml

rivaroxaban and apixaban respectively, are excreted in urine[21]. In addition, there is limited information available on the risk-benefit profile of DOACs in patients with severe renal function impairment[22-25] particularly in patients with diverse ethnic backgrounds[26]. Recently, high inter-individual variability in DOAC plasma levels was observed in clinical practice [27].

Our findings also differ from the results of a recent retrospective cohort study in patients with intracranial haemorrhage who were previously prescribed warfarin or DOACs. Prior use of DOACs compared with prior use of warfarin was associated with lower risk of hospital mortality[28]. A recent network meta-analysis by Lopez-Lopez also showed that DOACs are safer than warfarin in relation to major and intracranial bleeding[6]. However interestingly, in that meta-analysis the risk of gastrointestinal bleeding was higher with dabigatran, edoxaban and rivaroxaban than with warfarin. In addition, edoxaban (30mg and 60 mg twice daily) significantly increased the risk of clinically relevant bleeding compared with warfarin[6]. Although our study uses a longitudinal ecological study design, at least in part, our results are comparable with previous studies. In a large (>59,000 participants) population-based observational study conducted in Canada and the US, the risk of hospital admissions for major bleeding or all-cause mortality in the first 90 days of treatment was similar for DOACs and warfarin [29]. Furthermore, in a study that used an US commercial database of 38 million people warfarin users were hospitalised longer, stayed longer in an intensive care units than dabigatran or rivaroxaban users, but there was no difference in 30- or 90-day all-cause mortality[30].

It has been estimated that the cost of DOACs will rise sharply before patent expiry (in 2022) and by the year 2020 will constitute approximately 5% of the total NHS drug budget[14]. The drugs budget is the NHS's second biggest cost after its staff[31]. Cost of warfarin including INR monitoring for one patient per annum has been estimated to be £220 (https://www.gwh.nhs.uk/media/236108/doacs-for-dvt-pe-august-2016-v-9.pdf), while the cost of rivaroxaban (15mg bd for 3/52 loading (provided by hospital), then 20mg o.d. has been estimated to £657 p.a. (https://www.gwh.nhs.uk/media/236108/doacs-for-dvt-pe-august-2016-v-9.pdf). Similar costs have been estimated for other DOACs. Additionally, there is a huge discrepancy in price of agents that are used to reverse the anticoagulant effects of warfarin

For peer review only - http://bmjopen.bmj.com/site/about/guidelines.xhtml

(antidote vitamin K at £0.38) or DOACs (idarucizumab, reversal agent for dabigatran at £2,400 for a single treatment course of 2X 2.5 g infusions (https://www.nice.org.uk/advice/esnm73) or andexanet alpha, reversal agent for factor Xa inhibitors approved by the FDA in May 2018, at approximately £1,500). These high costs of DOACs could be avoided if warfarin treatment is optimised, for example through novel methods such as genotype-guided dosing[32 33] and point-of-care INR monitoring[34], and only individuals who are at higher risk of developing bleeding events, for example those with variant alleles that increase the risk of bleeding from warfarin[35], could be prescribed DOACs. Genotype-guided dosing of warfarin has been shown to be cost-effective[36]. It has been shown previously that approximately 55% of variability in warfarin dose requirements can be estimated from clinical and genetic data of three polymorphisms in the warfarin molecular target (vitamin K epoxide reductase gene) and metabolising enzyme (CYP2C9 gene) and genotype-guided dosing can reduce the risk of major bleeding[37 38].

Conclusions

This study is one of a few studies in the UK[1 39 40] that have evaluated the association between the rise in prescribing of DOACs after changes in NICE guidance in 2014 and emergency hospital admissions for bleeding events. We found that the rate of emergency admissions for bleeding conditions increased to a greater extent in GP practices that were more likely to prescribe DOACs compared with warfarin. With rapidly increasing use of DOACs, these potential adverse consequences need to be taken into account when assessing the benefits and costs of anticoagulant treatment in clinical practice. It is not clear whether the DOACs are being prescribed without adequate notice being taken of restrictions and warnings in the summary of product characteristics, and in addition, whether closer monitoring is required in certain patient groups to increase the safety of use in clinical practice. The recently announced review by the EMA is thus important in order to further optimise the use of all oral anticoagulants so that risks are minimised while at the same time maximising benefits.

For peer review only - http://bmjopen.bmj.com/site/about/guidelines.xhtml

PPI statement

The study was supported by a patient advisory group, which provided input to the NIHR- CLAHRC NWC programme of research. This patient advisory group met on a regular basis for the duration of the study. Due to data protection restrictions and the very technical methods required to do our data analyses they were not involved in the analysis part of the project. PPI representatives will however, write a plain language summary and design a leaflet for dissemination to their peers and distributing to patient groups.

Author contributions

Ana Alfirevic- Substantial contributions to the conception and design of the work, drafting a manuscript. Agreed to be accountable for all aspects of the work in ensuring that questions related to the accuracy or integrity of any part of the work are appropriately investigated and resolved.

Ben Barr- Substantial contribution to the design of the work, the acquisition, analysis and interpretation of data. Agreed to be accountable for all aspects of the work in ensuring that questions related to the accuracy or integrity of any part of the work are appropriately investigated and resolved.

Munir Pirmohamed- Substantial contribution to the interpretation of the results, critical revision of the manuscript for important intellectual content and final approval of the manuscript.

Jennifer Downing- Critical revision of the manuscript for important intellectual content and final approval of the manuscript.

Konstantinos Daras- Substantial contribution to data acquisition, data analysis and interpretation of data for the work. Critical revision of the manuscript for important intellectual content and final approval of the manuscript

Terence Comerford- Critical revision of the manuscript for important intellectual content and final approval of the manuscript. Preparation of plain language summary for dissemination of findings.

Patient consent for publication: *Not required.*
Funding: *This work was supported by the National Institute for Health Research, Collaboration for Leadership and Health Research and Care North West Coast (NIHR CLAHRC NWC) and the state Department of Health and Social Care.*
Disclaimer: *The views expressed are those of the author(s) and not necessarily those of the NHS, the NIHR or the Department of Health.*
Competing interests: *We have read and understood BMJ policy on declaration of interests and declare the following: All authors have completed the ICMJE uniform disclosure form at http://www.icmje.org/coi_disclosure.pdf and declare: no support from any organisation for the submitted work [or describe if any]; no financial relationships with any organisations that might have an interest in the submitted work in the previous three years [or describe if any], no other relationships or activities that could appear to have influenced the submitted work [or describe if any].*

Provenance and peer review: *Not commissioned; externally peer reviewed.*
Data sharing statement: *The dataset from this study will be made available on request to benbarr@liverpool.ac.uk*

For peer review only - http://bmjopen.bmj.com/site/about/guidelines.xhtml

References

1. Vinogradova Y, Coupland C, Hill T, et al. Risks and benefits of direct oral anticoagulants versus warfarin in a real world setting: cohort study in primary care. *BMJ (Clinical research ed* 2018;362:k2505. doi: 10.1136/bmj.k2505
2. Agnelli G, Buller HR, Cohen A, et al. Oral apixaban for the treatment of acute venous thromboembolism. *The New England journal of medicine* 2013;369(9):799-808. doi: 10.1056/NEJMoa1302507
3. Investigators E, Bauersachs R, Berkowitz SD, et al. Oral rivaroxaban for symptomatic venous thromboembolism. *The New England journal of medicine* 2010;363(26):2499-510. doi: 10.1056/NEJMoa1007903
4. Investigators E-P, Buller HR, Prins MH, et al. Oral rivaroxaban for the treatment of symptomatic pulmonary embolism. *The New England journal of medicine* 2012;366(14):1287-97. doi: 10.1056/NEJMoa1113572
5. Schulman S, Kearon C, Kakkar AK, et al. Dabigatran versus warfarin in the treatment of acute venous thromboembolism. *The New England journal of medicine* 2009;361(24):2342-52. doi: 10.1056/NEJMoa0906598
6. Lopez-Lopez JA, Sterne JAC, Thom HHZ, et al. Oral anticoagulants for prevention of stroke in atrial fibrillation: systematic review, network meta-analysis, and cost effectiveness analysis. *BMJ (Clinical research ed* 2017;359:j5058. doi: 10.1136/bmj.j5058
7. Protty MB, Hayes J. Dawn of the direct-acting oral anticoagulants: trends in oral anticoagulant prescribing in Wales 2009-2015. *Journal of clinical pharmacy and therapeutics* 2017;42(2):132-34. doi: 10.1111/jcpt.12481
8. Burnett A, Siegal D, Crowther M. Specific antidotes for bleeding associated with direct oral anticoagulants. *BMJ (Clinical research ed* 2017;357:j2216. doi: 10.1136/bmj.j2216
9. Mueller T, Alvarez-Madrazo S, Robertson C, et al. Comparative safety and effectiveness of direct oral anticoagulants in patients with atrial fibrillation in clinical practice in Scotland. *British journal of clinical pharmacology* 2019;85(2):422-31. doi: 10.1111/bcp.13814
10. Gunasekara FI, Richardson K, Carter K, et al. Fixed effects analysis of repeated measures data. *International journal of epidemiology* 2014;43(1):264-9. doi: 10.1093/ije/dyt221 [published Online First: 2013/12/25]
11. Rogers W. Regression standard errors in clustered samples. *Stata Technical Bulletin* 1994;3
12. Shadish WR, Cook TD, Thomas. CD. Experimental and Quasi-Experimental Designs for Generalised Causal Inference: Houghton Mifflin 2002.
13. Obamiro KO, Chalmers L, Bereznicki LR. A Summary of the Literature Evaluating Adherence and Persistence with Oral Anticoagulants in Atrial Fibrillation. *American journal of cardiovascular drugs : drugs, devices, and other interventions* 2016;16(5):349-63. doi: 10.1007/s40256-016-0171-6
14. Burn J, Pirmohamed M. Direct oral anticoagulants versus warfarin: is new always better than the old? *Open heart* 2018;5(1):e000712. doi: 10.1136/openhrt-2017-000712
15. Connolly SJ, Ezekowitz MD, Yusuf S, et al. Dabigatran versus warfarin in patients with atrial fibrillation. *The New England journal of medicine* 2009;361(12):1139-51. doi: 10.1056/NEJMoa0905561
16. Ezekowitz MD, Connolly S, Parekh A, et al. Rationale and design of RE-LY: randomized evaluation of long-term anticoagulant therapy, warfarin, compared with dabigatran. *American heart journal* 2009;157(5):805-10, 10 e1-2. doi: 10.1016/j.ahj.2009.02.005

For peer review only - http://bmjopen.bmj.com/site/about/guidelines.xhtml

17. Ogawa S, Shinohara Y, Kanmuri K. Safety and efficacy of the oral direct factor xa inhibitor apixaban in Japanese patients with non-valvular atrial fibrillation. -The ARISTOTLE-J study. *Circulation journal : official journal of the Japanese Circulation Society* 2011;75(8):1852-9.
18. Patel MR, Mahaffey KW, Garg J, et al. Rivaroxaban versus warfarin in nonvalvular atrial fibrillation. *The New England journal of medicine* 2011;365(10):883-91. doi: 10.1056/NEJMoa1009638
19. Ruff CT, Giugliano RP, Antman EM, et al. Evaluation of the novel factor Xa inhibitor edoxaban compared with warfarin in patients with atrial fibrillation: design and rationale for the Effective aNticoaGulation with factor xA next GEneration in Atrial Fibrillation-Thrombolysis In Myocardial Infarction study 48 (ENGAGE AF-TIMI 48). *American heart journal* 2010;160(4):635-41. doi: 10.1016/j.ahj.2010.06.042
20. Weitz JI, Connolly SJ, Patel I, et al. Randomised, parallel-group, multicentre, multinational phase 2 study comparing edoxaban, an oral factor Xa inhibitor, with warfarin for stroke prevention in patients with atrial fibrillation. *Thromb Haemost* 2010;104(3):633-41. doi: 10.1160/TH10-01-0066
21. Lutz J, Jurk K, Schinzel H. Direct oral anticoagulants in patients with chronic kidney disease: patient selection and special considerations. *International journal of nephrology and renovascular disease* 2017;10:135-43. doi: 10.2147/IJNRD.S105771
22. Buckley LF, Rybak E, Aldemerdash A, et al. Direct oral anticoagulants in patients with atrial fibrillation and renal impairment, extremes in weight, or advanced age. *Clinical cardiology* 2017;40(1):46-52. doi: 10.1002/clc.22591
23. Fanikos J, Burnett AE, Mahan CE, et al. Renal Function and Direct Oral Anticoagulant Treatment for Venous Thromboembolism. *The American journal of medicine* 2017;130(10):1137-43. doi: 10.1016/j.amjmed.2017.06.004
24. Fanikos J, Burnett AE, Mahan CE, et al. Renal Function Considerations for Stroke Prevention in Atrial Fibrillation. *The American journal of medicine* 2017;130(9):1015-23. doi: 10.1016/j.amjmed.2017.04.015
25. Zou R, Tao J, Shi W, et al. Meta-analysis of safety and efficacy for direct oral anticoagulation treatment of non-valvular atrial fibrillation in relation to renal function. *Thrombosis research* 2017;160:41-50. doi: 10.1016/j.thromres.2017.10.013
26. Wang KL, Chiu CC, Giugliano RP, et al. Drug Class, Renal Elimination, and Outcomes of Direct Oral Anticoagulants in Asian Patients: A Meta-Analysis. *J Stroke Cerebrovasc Dis* 2018;27(4):857-64. doi: 10.1016/j.jstrokecerebrovasdis.2017.10.027
27. Testa S, Tripodi A, Legnani C, et al. Plasma levels of direct oral anticoagulants in real life patients with atrial fibrillation: Results observed in four anticoagulation clinics. *Thrombosis research* 2016;137:178-83. doi: 10.1016/j.thromres.2015.12.001 [published Online First: 2015/12/18]
28. Inohara T, Xian Y, Liang L, et al. Association of Intracerebral Hemorrhage Among Patients Taking Non-Vitamin K Antagonist vs Vitamin K Antagonist Oral Anticoagulants With In-Hospital Mortality. *JAMA* 2018;319(5):463-73. doi: 10.1001/jama.2017.21917
29. Jun M, Lix LM, Durand M, et al. Comparative safety of direct oral anticoagulants and warfarin in venous thromboembolism: multicentre, population based, observational study. *BMJ (Clinical research ed* 2017;359:j4323. doi: 10.1136/bmj.j4323
30. Charlton B, Adeboyeje G, Barron JJ, et al. Length of hospitalization and mortality for bleeding during treatment with warfarin, dabigatran, or rivaroxaban. *PLoS One* 2018;13(3):e0193912. doi: 10.1371/journal.pone.0193912
31. The L. Personalised medicine in the UK. *Lancet* 2018;391(10115):e1. doi: 10.1016/S0140-6736(17)33261-0

For peer review only - http://bmjopen.bmj.com/site/about/guidelines.xhtml

32. Yang T, Zhou Y, Chen C, et al. Genotype-guided dosing versus conventional dosing of warfarin: A meta-analysis of 15 randomized controlled trials. *Journal of clinical pharmacy and therapeutics* 2018 doi: 10.1111/jcpt.12782
33. Jorgensen AL, Prince C, Fitzgerald G, et al. Implementation of genotype-guided dosing of warfarin with point-of-care genetic testing in three UK clinics: a matched cohort study. *BMC Med* 2019;17(1):76. doi: 10.1186/s12916-019-1308-7
34. Wool GD. Benefits and Pitfalls of Point-of-Care Coagulation Testing for Anticoagulation Management: An ACLPS Critical Review. *American journal of clinical pathology* 2019;151(1):1-17. doi: 10.1093/ajcp/aqy087
35. Kawai VK, Cunningham A, Vear SI, et al. Genotype and risk of major bleeding during warfarin treatment. *Pharmacogenomics* 2014;15(16):1973-83. doi: 10.2217/pgs.14.153
36. Pink J, Pirmohamed M, Lane S, et al. Cost-effectiveness of pharmacogenetics-guided warfarin therapy vs. alternative anticoagulation in atrial fibrillation. *Clin Pharmacol Ther* 2014;95(2):199-207. doi: 10.1038/clpt.2013.190
37. Gage BF, Bass AR, Lin H, et al. Effect of Genotype-Guided Warfarin Dosing on Clinical Events and Anticoagulation Control Among Patients Undergoing Hip or Knee Arthroplasty: The GIFT Randomized Clinical Trial. *JAMA* 2017;318(12):1115-24. doi: 10.1001/jama.2017.11469
38. Pirmohamed M, Burnside G, Eriksson N, et al. A randomized trial of genotype-guided dosing of warfarin. *The New England journal of medicine* 2013;369(24):2294-303. doi: 10.1056/NEJMoa1311386
39. Green L, Tan J, Antoniou S, et al. Haematological management of major bleeding associated with direct oral anticoagulants - UK experience. *Br J Haematol* 2019 doi: 10.1111/bjh.15808
40. Green L, Tan J, Morris JK, et al. A three-year prospective study of the presentation and clinical outcomes of major bleeding episodes associated with oral anticoagulant use in the UK (ORANGE study). *Haematologica* 2018;103(4):738-45. doi: 10.3324/haematol.2017.182220

For peer review only

For peer review only - http://bmjopen.bmj.com/site/about/guidelines.xhtml

Figure legends

Figure 1. Trend in anticoagulant prescribing items and costs from GP practices in England and the proportion of direct oral anticoagulants of all anticoagulants prescribed. In 2014, NICE guidance on use of direct oral anticoagulants has changed.

Figure 2. Geographical pattern in DOAC prescribing as a proportion of all anticoagulant prescribing items in England 2011-2016 that includes changes in NICE guidance in 2014.

Figure 3 Estimates from regression model showing the relative change in the rate of emergency admissions for bleeding and clotting complications associated with each additional 10% of DOACs prescribed as a proportion of all anticoagulants.

Figure 4. Trend in emergency admissions for bleeding conditions between 2011 and 2016 and the trend, estimated from the regression model that would have been expected if DOACs had not been introduced.

For peer review only

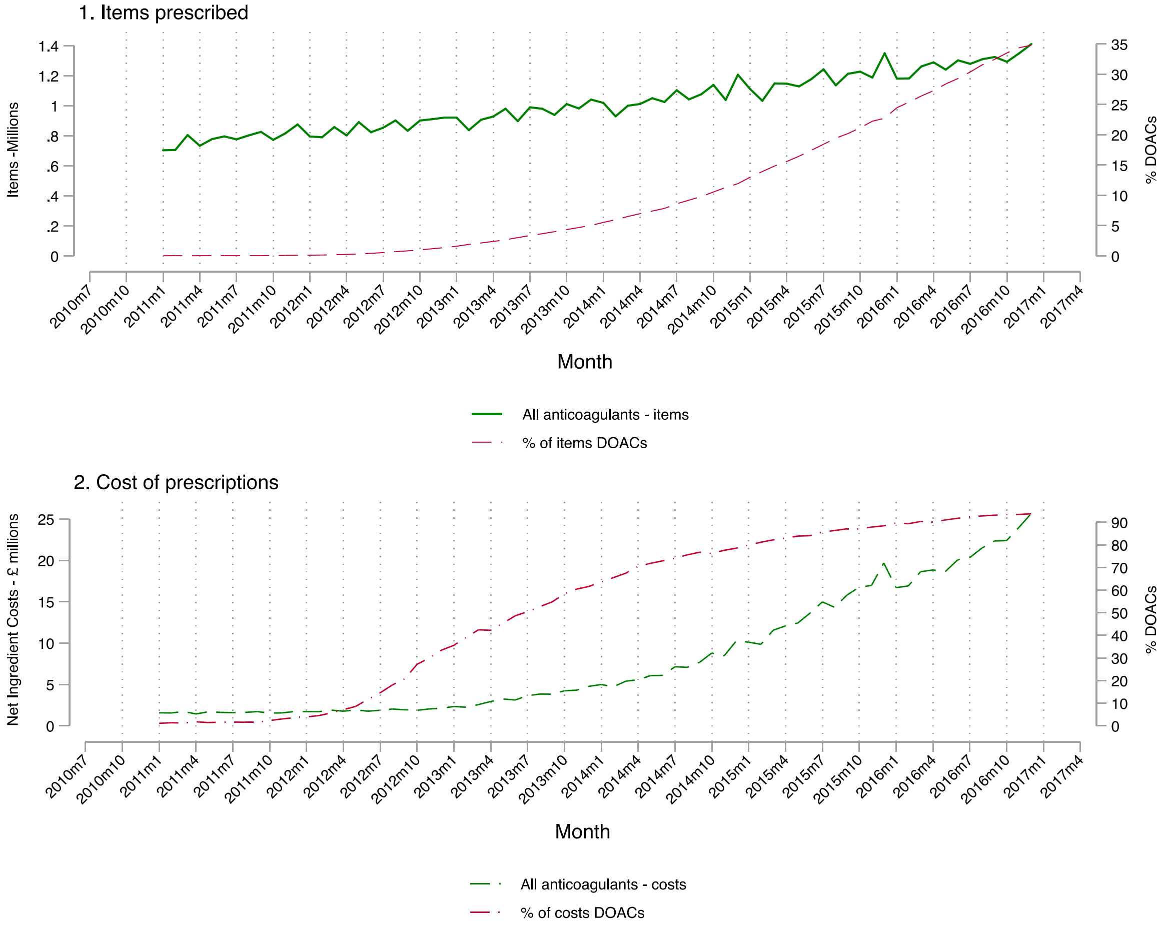
1. Items prescribed
Items -Millions
% DOACs
Month
All anticoagulants - items
% of items DOACs
2. Cost of prescriptions
Net Ingredient Costs - £ millions
% DOACs
Month
All anticoagulants - costs
% of costs DOACs

ew only

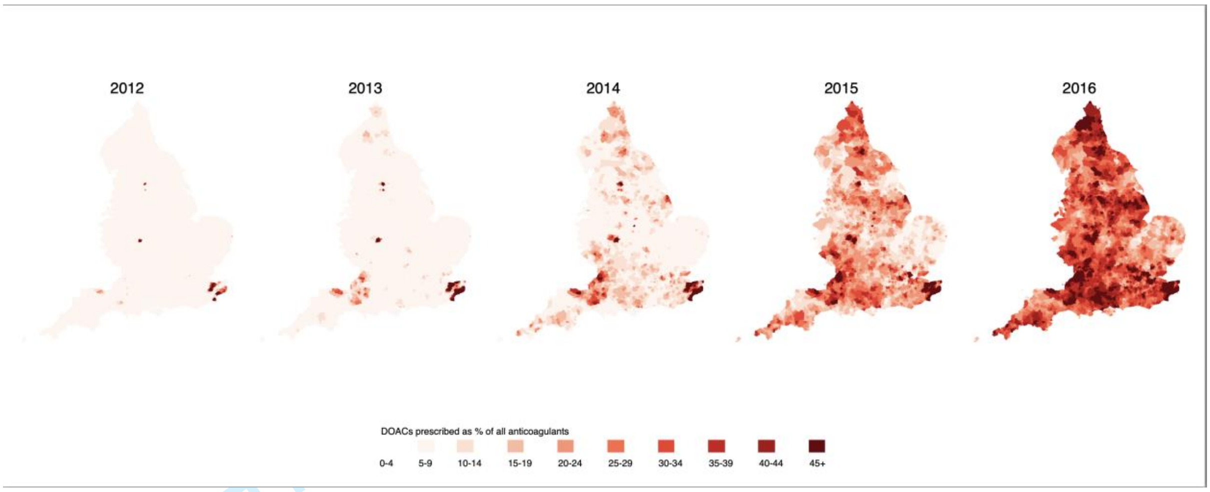

For peer review only

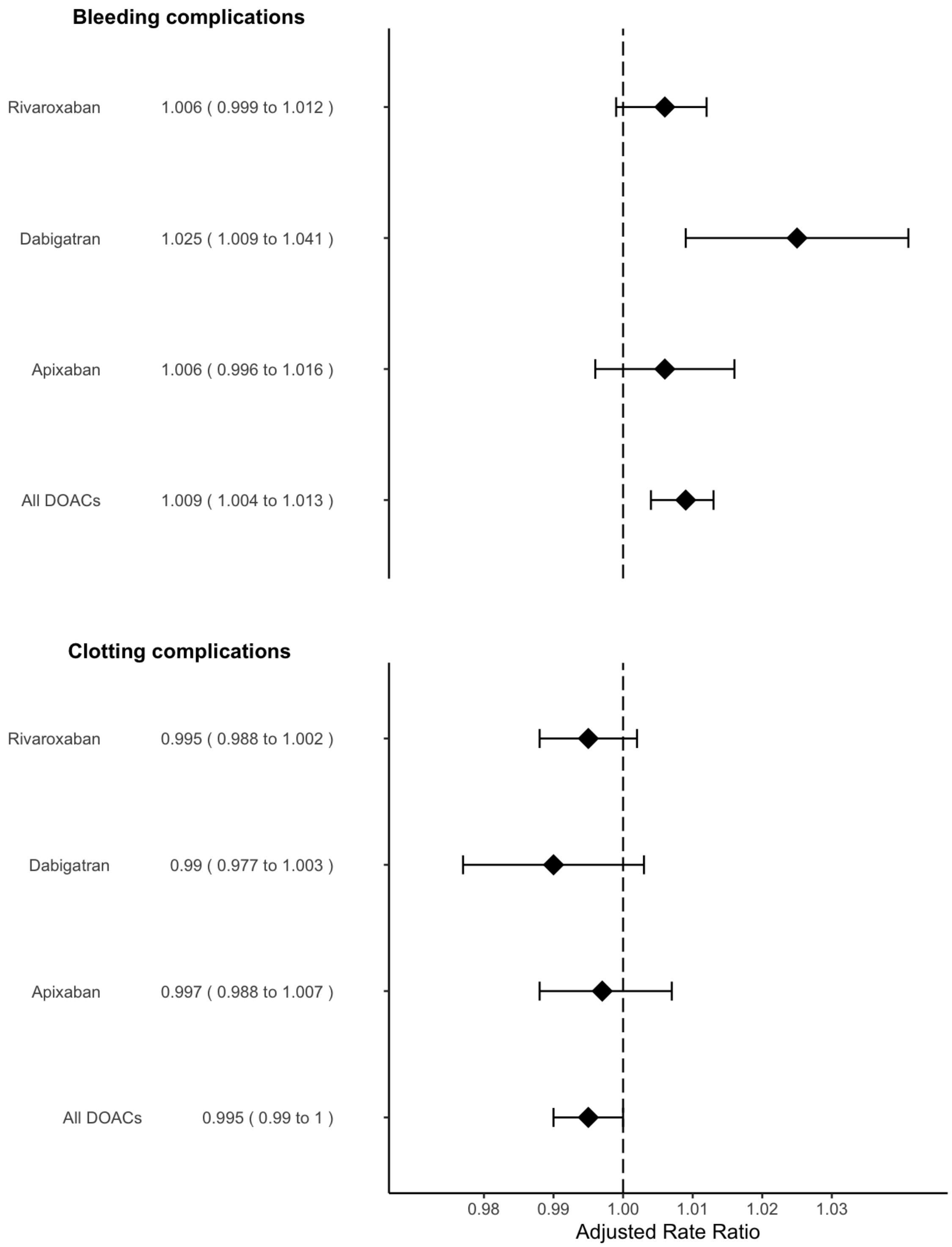
Bleeding complications
Rivaroxaban
1.006 (0.999 to 1.012)
Dabigatran
1.025 (1.009 to 1.041)
Apixaban
1.006 (0.996 to 1.016)
All DOACs
1.009 (1.004 to 1.013)
Clotting complications
Rivaroxaban
0.995 (0.988 to 1.002)
Dabigatran
0.99 (0.977 to 1.003)
Apixaban
0.997 (0.988 to 1.007)
All DOACs
0.995 (0.99 to 1)
0.98
0.99
1.00
1.01
1.02
1.03
Adjusted Rate Ratio

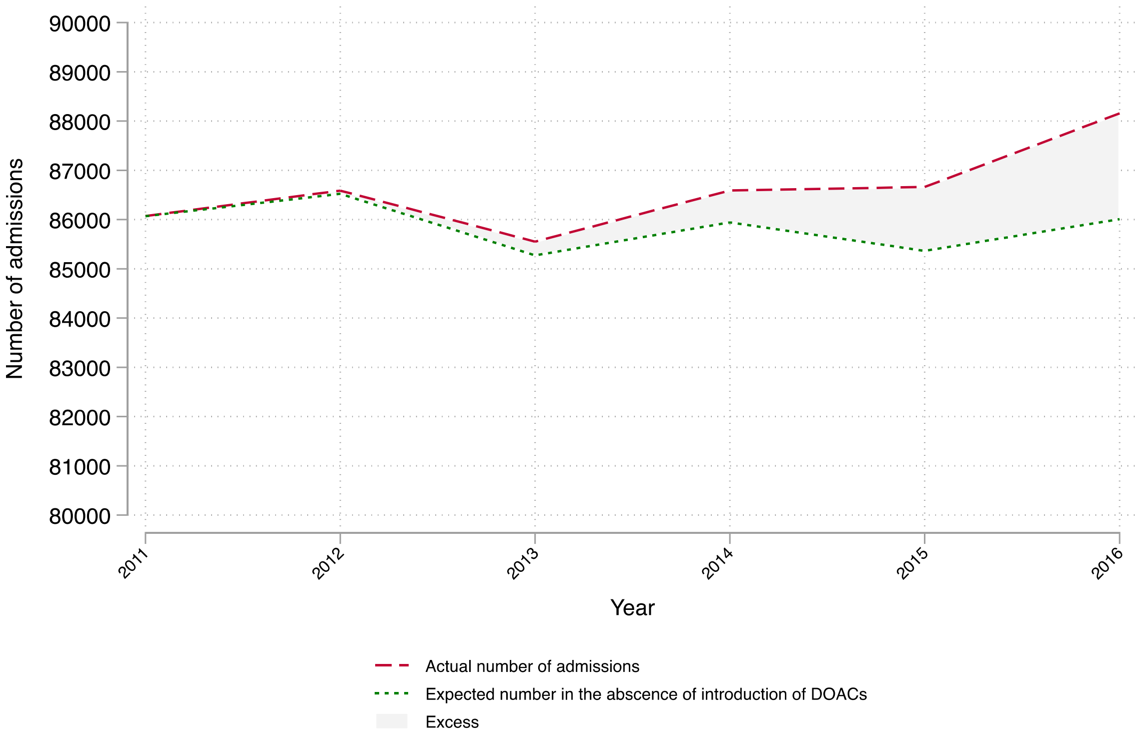

r review only

Appendix 1 BNF codes and ICD10 codes used to define exposure and outcome variables.

BNF codes used to define prescribing

0208020V - Warfarin

0208020X0 – Dabigatran

0208020X0 – edoxaban

0208020Y – rivaroxaban

0208020Z0 - Apixiban

ICD 10 Codes used to define outcomes

1. **Bleeding Complications**

ICD10 code	Name
M79.81	traumatic haematoma of the soft tissue
M25.00	Hemarthrosis, unspecified joint
K92	Other diseases of digestive system
K92.0	Haematemesis
K92.1	Melaena
K92.2	Gastrointestinal haemorrhage, unspecified
K62.5	Haemorrhage of anus and rectum
R58	Haemorrhage, not elsewhere classified
R04.0	Epistaxis
R04.1	Haemorrhage from throat
R04.2	Haemoptysis
R04.8	Haemorrhage from other sites in respiratory passages

For peer review only

R04.9	Haemorrhage from respiratory passages, unspecified
R31	Unspecified haematuria
N02	Recurrent or persistent haematuria
I60	Subarachnoid haemorrhage
I60.0	Subarachnoid haemorrhage from carotid siphon and bifurcation
I60.1	Subarachnoid haemorrhage from middle cerebral artery
I60.2	Subarachnoid haemorrhage from anterior communicating artery
I60.3	Subarachnoid haemorrhage from posterior communicating artery
I60.4	Subarachnoid haemorrhage from basilar artery
I60.5	Subarachnoid haemorrhage from vertebral artery
I60.6	Subarachnoid haemorrhage from other intracranial arteries
I60.7	Subarachnoid haemorrhage from intracranial artery, unspecified
I60.8	Other subarachnoid haemorrhage
I60.9	Subarachnoid haemorrhage, unspecified
I61	Intracerebral haemorrhage
I61.0	Intracerebral haemorrhage in hemisphere, subcortical
I61.1	Intracerebral haemorrhage in hemisphere, cortical
I61.2	Intracerebral haemorrhage in hemisphere, unspecified
I61.3	Intracerebral haemorrhage in brain stem
I61.4	Intracerebral haemorrhage in cerebellum
I61.5	Intracerebral haemorrhage, intraventricular
I61.6	Intracerebral haemorrhage, multiple localized
I61.8	Other intracerebral haemorrhage
I61.9	Intracerebral haemorrhage, unspecified
I62	Other nontraumatic intracranial haemorrhage
I62.0	Subdural haemorrhage (acute)(nontraumatic)
I62.1	Nontraumatic extradural haemorrhage

For peer review only

I62.9	Intracranial haemorrhage (nontraumatic), unspecified

2. **Clotting Complications**

**ICD10 code**	**Name**
I63	Cerebral infarction
I64	Stroke, not specified as haemorrhage or infarction
I26	Pulmonary embolism
I82	Other venous embolism and thrombosis
I80.0	Phlebitis and thrombophlebitis of superficial vessels of lower extremities
I80.1	Phlebitis and thrombophlebitis of femoral vein
I80.2	Deep vein thrombosis, Phlebitis and thrombophlebitis of other deep vessels of lower extremities
I80.3	Phlebitis and thrombophlebitis of lower extremities, unspecified
I80.8	Phlebitis and thrombophlebitis of other sites
I80.9	Phlebitis and thrombophlebitis of unspecified site

For peer review only

Appendix 2 flow outlining inclusion/ exclusion and missing data

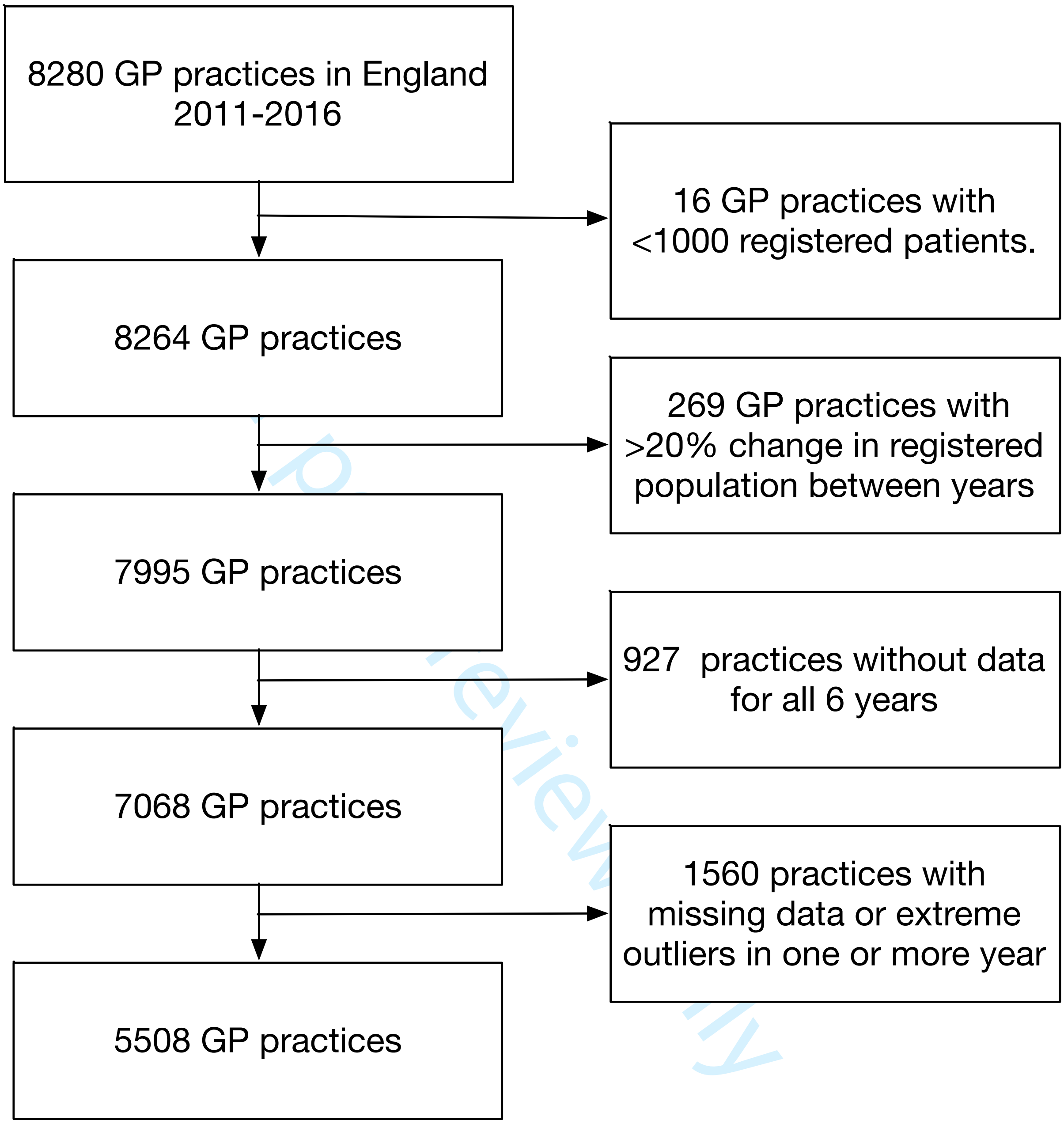

Appendix 3 alternative model specifications.

1. **Model using all GP practices – without applying exclusion criteria (8032 GP practices – 248 with missing data). Estimates from regression model showing the relative change in the rate of emergency admissions for bleeding and clotting complications associated with each additional 10% of DOACs prescribed as a proportion of all anticoagulants**

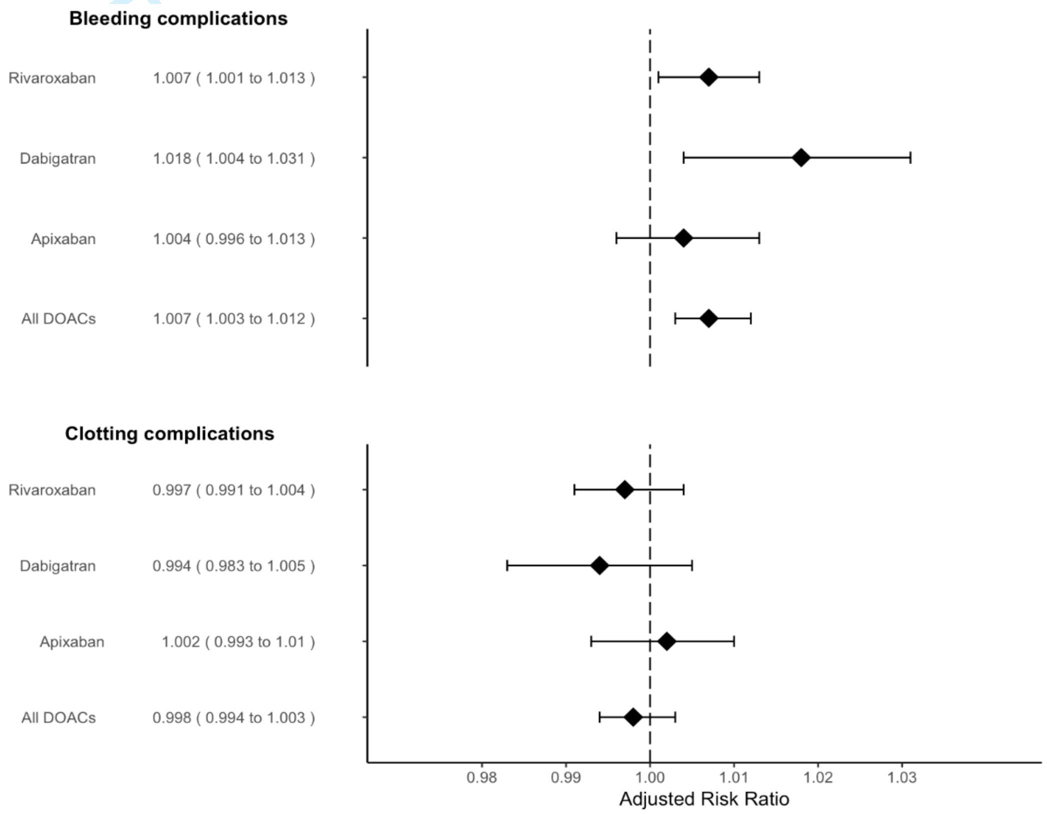

2. Negative Binomial Model

Estimates from regression model showing the relative change in the rate of emergency admissions for bleeding and clotting complications associated with each additional 10% of DOACs prescribed as a proportion of all anticoagulants

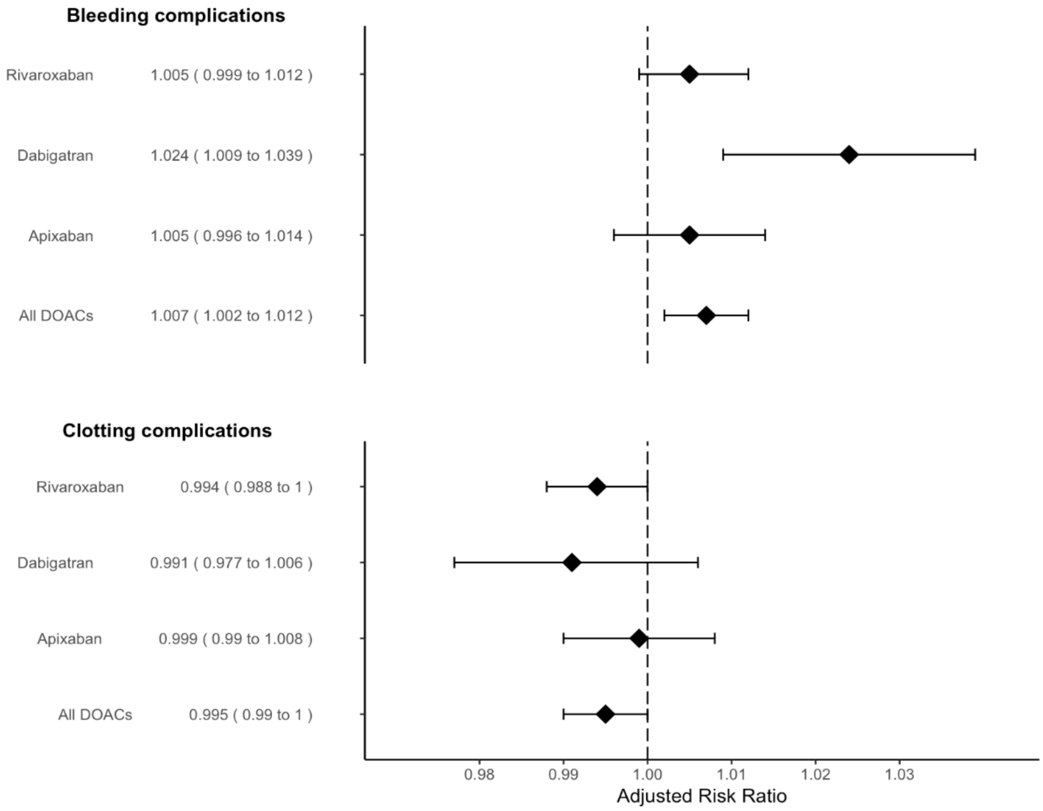

only

3. Non-equivalent dependent variable.

Estimates from regression model showing the relative change in the rate of emergency admissions for gastrointestinal admissions associated with each additional 10% of DOACs prescribed as a proportion of all anticoagulants.

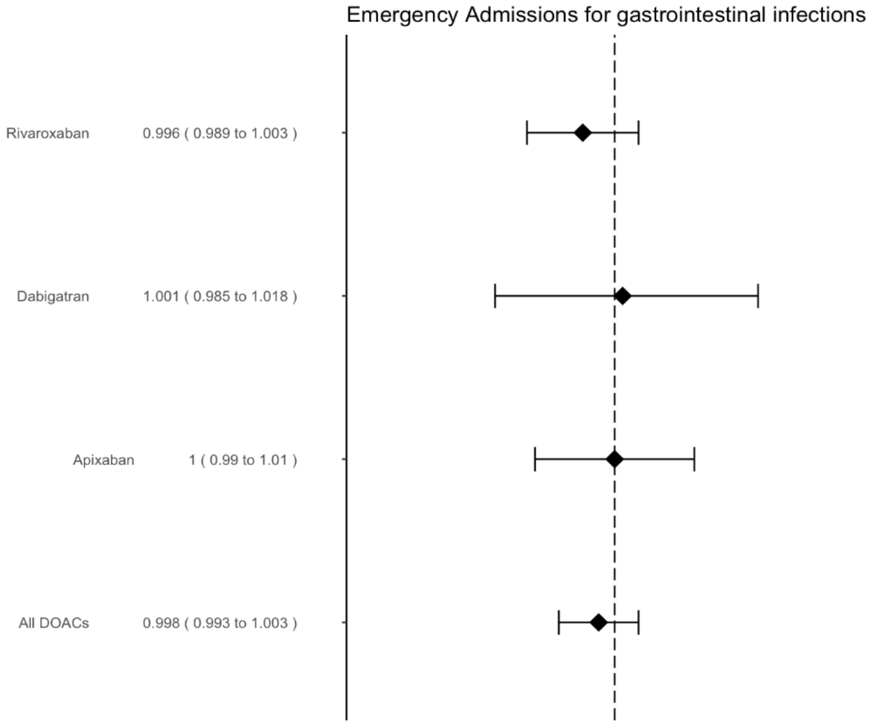

For peer review only

4. General Estimating equation - with exchangeable correlation structure.

Estimates from regression model showing the relative change in the rate of emergency admissions for bleeding and clotting complications associated with each additional 10% of DOACs prescribed as a proportion of all anticoagulants

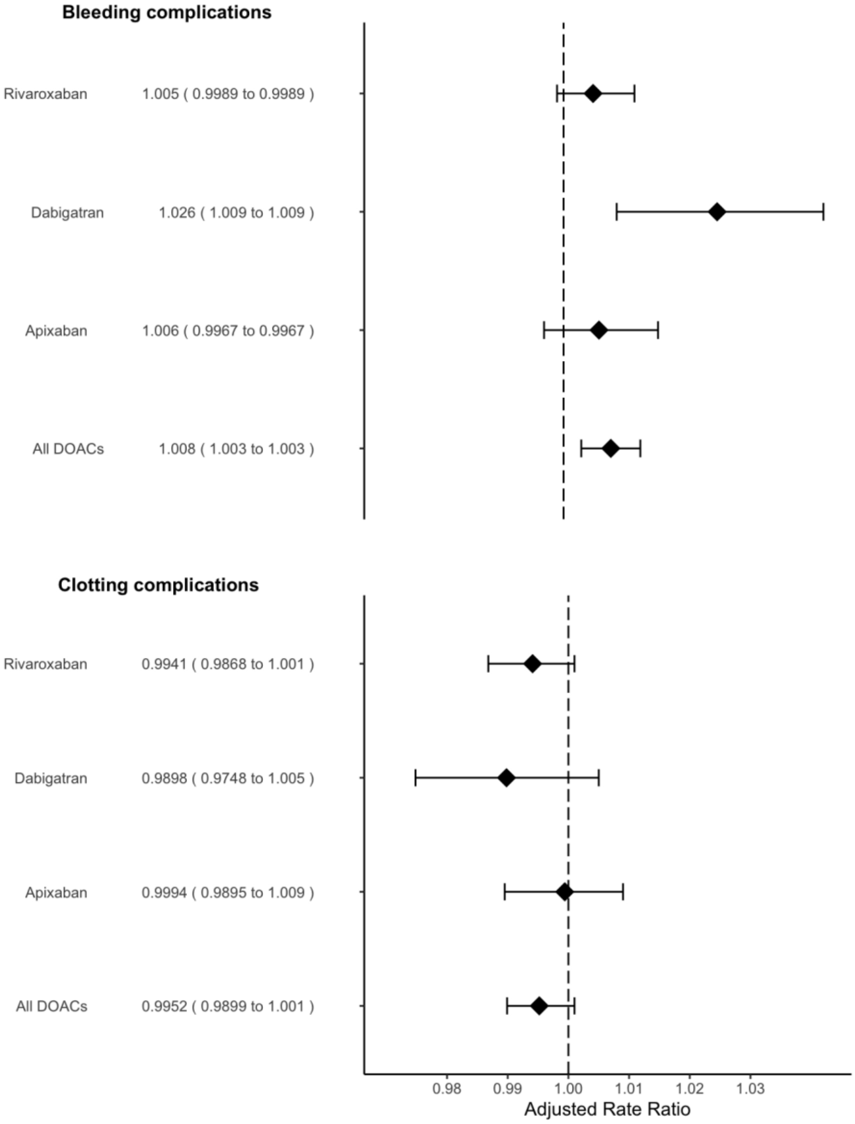

5. General Estimating equation - autoregressive(AR-1) correlation structure. Estimates from regression model showing the relative change in the rate of emergency admissions for bleeding and clotting complications associated with each additional 10% of DOACs prescribed as a proportion of all anticoagulants

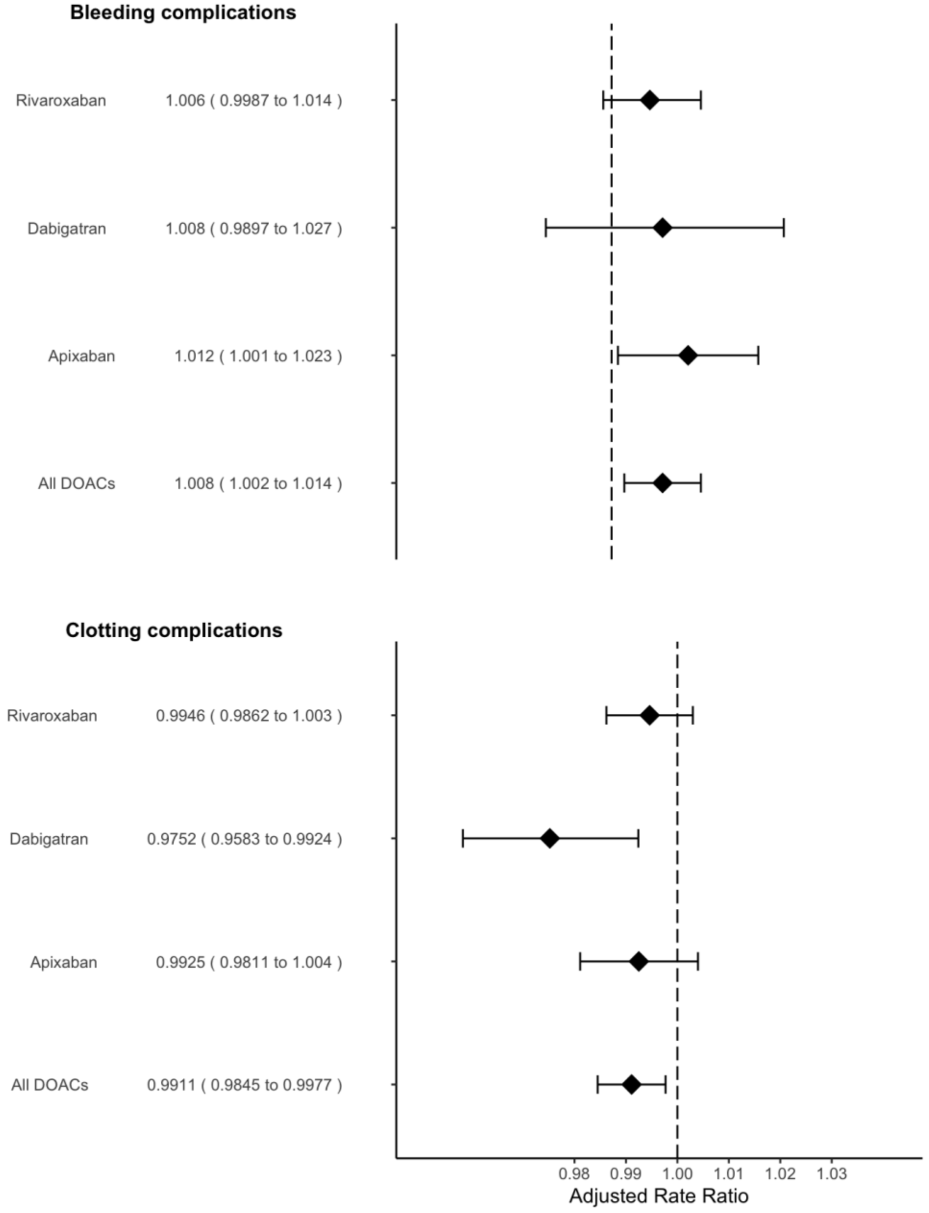

STROBE Statement—checklist of items that should be included in reports of observational studies
Paper submission:
Alfirevic et al. Has the introduction of direct oral anticoagulants (DOACs) in England increased emergency admissions for bleeding conditions? A longitudinal ecological study.

	Item No	Recommendation
**Title and abstract**	1	(*a*) We've indicated the study's design in the title or the abstract
		(*b*) We've provided in the abstract an informative and balanced summary of what was done and what was found, page 2
**Introduction**		
Background/rationale	2	We've explained the scientific background and rationale for the investigation being reported, pages 3 and 4
Objectives	3	State specific objectives, including any prespecified hypotheses- done, page 4, line 12 to 18
**Methods**		
Study design	4	Key elements of study design were presented on page 5, lines 12 to 27
Setting	5	The setting, locations, and relevant dates, including periods of recruitment, exposure, follow-up, and data collection were presented on pages 4 and 5
Participants	6	We included all GP practices in England with a registered population size greater than 1000, which had data for all 6 years (N=5508 GP practices with 33,048 practice years of data. Page 5
		Inclusions/exclusions and missing data are outlined in Supplementary material- Appendix 2
Variables	7	Clearly defined outcomes (BNF and ICD10 codes) are presented in Supplementary material, Appendix 1
Data sources/ measurement	8*	Sources of data and details of methods of assessment are presented on pages 4 and 5. Describe comparability of assessment methods if there is more than one group
Bias	9	Potential sources of bias such as confounding from unobserved factors that vary between GP practice populations, we used a fixed effects approach and a dummy variable for each year to account for the national trend in prescribing and emergency admissions. In addition, we performed sensitivity analyses
Study size	10	All eligible GP practices in England were included in this study
Quantitative variables	11	We've explained how quantitative variables were handled in the analyses on pages 5 and 6
Statistical methods	12	(*a*) Describe all statistical methods, including those used to control for confounding- done, page 5 and 6
		(*b*) Describe any methods used to examine subgroups and interactions, done page 5 and 6
		(*c*) Explain how missing data were addressed- done flow chart Supplementary material Appendix 2
		(*e*) Sensitivity analyses are described on page 6

Continued on next page

For peer review only - http://bmjopen.bmj.com/site/about/guidelines.xhtml

**Results**		
Participants	13*	(a) Report numbers of individuals at each stage of study-done, Supplementary material Appendix 2
		(b) Give reasons for non-participation at each stage- done Appendix 2
		(c) Flow diagram- done and shown in Appendix 2
Descriptive data	14*	(a) Give characteristics of study participants – GP practice data included, no data on individual participants are available
		(b) We've indicated the number of practices with missing data in Appendix 2
		(c) *F*ollow-up time of 6 years has been clearly shown in the manuscript (page 5 and 7) and Figures 1 and 2
Outcome data	15*	
		Outcome data are described in the manuscript, page 7
Main results	16	(*a*) We have given unadjusted estimates and their precision (95% confidence interval), page 7
		(*c*) We haven't translated estimates of relative risk into absolute risk
Other analyses	17	We have reported sensitivity analyses, page 7, lines 50-59
**Discussion**		
Key results	18	Summarise key results with reference to study objectives- done, page 8
Limitations	19	Discuss limitations of the study, taking into account sources of potential bias or imprecision. Discuss both direction and magnitude of any potential bias- done page 9 line 22
Interpretation	20	Give a cautious overall interpretation of results considering objectives, limitations, multiplicity of analyses, results from similar studies, and other relevant evidence done page 10
Generalisability	21	Discuss the generalisability (external validity) of the study results – we have compared our results with previous studies, page 10
**Other information**		
Funding	22	Give the source of funding and the role of the funders for the present study and, if applicable, for the original study on which the present article is based- Done, we have included a sentence at the end of the manuscript to acknowledge our NIHR CLAHRC NWC funding

*Give information separately for cases and controls in case-control studies and, if applicable, for exposed and unexposed groups in cohort and cross-sectional studies.

Note: An Explanation and Elaboration article discusses each checklist item and gives methodological background and published examples of transparent reporting. The STROBE checklist is best used in conjunction with this article (freely available on the Web sites of PLoS Medicine at http://www.plosmedicine.org/, Annals of Internal Medicine at http://www.annals.org/, and Epidemiology at http://www.epidem.com/). Information on the STROBE Initiative is available at www.strobe-statement.org.

For peer review only

BMJ Open

Has the introduction of direct oral anticoagulants (DOACs) in England increased emergency admissions for bleeding conditions? A longitudinal ecological study.

Journal:	*BMJ Open*
Manuscript ID	bmjopen-2019-033357.R3
Article Type:	Original research
Date Submitted by the Author:	28-Feb-2020

Complete List of Authors:	Alfirevic, Ana; University of Liverpool, Molecular and Clinical Pharmacology<br>Downing, Jennifer; University of Liverpool, Molecular and Clinical Pharmacology<br>Daras, Konstantinos ; University of Liverpool School of Environmental Sciences, Geography and Planning<br>Comerford, Terence; University of Liverpool<br>Pirmohamed, Munir; University of Liverpool, Molecular and Clinical Pharmacology<br>Barr, Ben

**Primary Subject Heading**:	Public health
Secondary Subject Heading:	Pharmacology and therapeutics, Cardiovascular medicine
Keywords:	CLINICAL PHARMACOLOGY, PRIMARY CARE, PUBLIC HEALTH

SCHOLARONE™
Manuscripts

I, the Submitting Author has the right to grant and does grant on behalf of all authors of the Work (as defined in the below author licence), an exclusive licence and/or a non-exclusive licence for contributions from authors who are: i) UK Crown employees; ii) where BMJ has agreed a CC-BY licence shall apply, and/or iii) in accordance with the terms applicable for US Federal Government officers or employees acting as part of their official duties; on a worldwide, perpetual, irrevocable, royalty-free basis to BMJ Publishing Group Ltd ("BMJ") its licensees and where the relevant Journal is co-owned by BMJ to the co-owners of the Journal, to publish the Work in this journal and any other BMJ products and to exploit all rights, as set out in our licence.

The Submitting Author accepts and understands that any supply made under these terms is made by BMJ to the Submitting Author unless you are acting as an employee on behalf of your employer or a postgraduate student of an affiliated institution which is paying any applicable article publishing charge ("APC") for Open Access articles. Where the Submitting Author wishes to make the Work available on an Open Access basis (and intends to pay the relevant APC), the terms of reuse of such Open Access shall be governed by a Creative Commons licence – details of these licences and which Creative Commons licence will apply to this Work are set out in our licence referred to above.

Other than as permitted in any relevant BMJ Author's Self Archiving Policies, I confirm this Work has not been accepted for publication elsewhere, is not being considered for publication elsewhere and does not duplicate material already published. I confirm all authors consent to publication of this Work and authorise the granting of this licence.

For peer review only

Has the introduction of direct oral anticoagulants (DOACs) in England increased emergency admissions for bleeding conditions? A longitudinal ecological study.

Ana Alfirevic[1], Jennifer Downing[1], Konstantinos Daras[2], Terence Comerford[3], Munir Pirmohamed[1] and Ben Barr[2]

[1] The Wolfson Centre for Personalised Medicine, Department of Molecular and Clinical Pharmacology, Institute of Translational Medicine, University of Liverpool, UK

[2] Department of Public Health and Policy, Institute of Psychology, Health and Society, University of Liverpool, UK

[3] CLAHRC NWC Public Advisor Group, University of Liverpool, UK

Corresponding Author:

Ana Alfirevic

The Wolfson Centre for Personalised Medicine,

Department of Molecular and Clinical Pharmacology,

Institute of Translational Medicine

University of Liverpool

Block A, The Waterhouse Building

1-5 Brownlow Street

Liverpool, L69 3GL

Tel: +44 (0)151 7945551

E-mail: Ana.Alfirevic@liv.ac.uk

For peer review only

ABSTRACT

Objective. There is concern about long term safety of direct oral coagulants (DOACs) in clinical practice. Our aim was to investigate whether the introduction of DOACs compared with vitamin-K antagonists in England was associated with a change in admissions for bleeding or thromboembolic complications.

Setting: 5508 General Practitioner (GP) practices in England between 2011 and 2016.

Participants: All GP practices in England with a registered population size of greater than 1000 that had data for all 6 years.

Main outcome measure: The rate of emergency admissions to hospital for bleeding or thromboembolism, per 100,000 population for each GP practice in England.

Main exposure measure: The annual number of DOAC items prescribed for each GP practice population as a proportion of all anticoagulant items prescribed.

Design. This longitudinal ecological study used panel regression models to investigate the association between trends in DOAC prescribing within GP practice populations and trends in emergency admission rates for bleeding and thromboembolic conditions, whilst controlling for confounders.

Results. For each additional 10% of DOACs prescribed as a proportion of all anticoagulants, there was a 0.9% increase in bleeding complications (rate ratio (RR) 1.008 95% confidence intervals (CI) 1.003 to 1.013). The introduction of DOACs between 2011 and 2016 was associated with additional 4929 (95%CI 2489 to 7370) emergency admissions for bleeding complications. Increased DOAC prescribing was associated with a slight decline in admission for thromboembolic conditions.

Conclusion. Our data show that the rapid increase in prescribing of DOACs after changes in the NICE guidance in 2014 may have been associated with a higher rate of emergency admissions for bleeding conditions. These consequences need to be considered in assessing the benefits and costs of the widespread use of DOACs.

Word count 282

Key words Direct oral anticoagulants, warfarin, emergency admissions, bleeding, thromboembolism

For peer review only

For peer review only - http://bmjopen.bmj.com/site/about/guidelines.xhtml

Article Summary

Strengths and limitations of this study

- The majority of GP practices in England were included in our analyses and therefore we could estimate effects across the whole population
- Longitudinal data analyses included the changes in the guidelines for prescribing anticoagulants
- We used effectiveness and safety data on anticoagulants used in England
- It was not possible to link information on the prescribing for specific individuals to particular hospital admissions due to the ecological study design
- No control for adherence to medications was possible

For peer review only

For peer review only - http://bmjopen.bmj.com/site/about/guidelines.xhtml

Introduction

Prescribing of direct oral anticoagulants (DOACs) such as dabigatran, rivaroxaban, apixaban and edoxaban that were licenced between 2013 and 2015, has increased rapidly in the UK. DOACs are used for stroke prevention in atrial fibrillation (AF), treatment of venous thromboembolism (VTE), and medical and surgical thromboprophylaxis.

Warfarin has been the primary oral anticoagulant in patients with AF and VTE for over 60 years. Its use is associated with large inter-individual variability in dose requirements, narrow therapeutic index, slow onset of action, low time in therapeutic range and concomitant drug and food interactions. In addition, regular International Normalised Ratio (INR) monitoring required for optimising loading and maintenance doses is burdensome to the patients and healthcare professionals. The DOACs are recommended by NICE guidelines for the treatment of adults with venous thromboembolism, prevention of recurrent deep vein thrombosis, and stroke prevention in patients with non-valvular atrial fibrillation[1]. Several non-inferiority randomised controlled trials have demonstrated that DOACs have comparable efficacy and safety profiles with warfarin [2-5]. In addition, in clinical trials fixed-dose administration was proposed for DOACs, without the need for routine laboratory monitoring. This has led to widespread uptake of DOACs into clinical practice. Although DOACs have been shown to be cost-effective in RCTs[6], their high cost compared with the cost of warfarin (inclusive of INR testing) has major budget implications for the NHS in the UK and other healthcare services globally[7]. Concern about the escalating costs to healthcare is increasing not only for DOACs, but also when complications occur, for their reversal agents such as idarucizumab or andexanet alpha[8].

Data on the comparative safety and efficacy of DOACs in real-life observational studies has also become available more recently[6]. For instance, a recent large observational study in the UK found that apixaban was associated with decreased risk of bleeding complications, but rivaroxaban and low dose apixaban were associated with an increased risk of all-cause mortality compared to warfarin[1]. A large study (>14,500 patients) conducted in Scotland demonstrated that patients taking rivaroxaban may be

For peer review only - http://bmjopen.bmj.com/site/about/guidelines.xhtml

at increased risk of bleeding compared with other DOACs[9]. Recent concern about bleeding risks associated with DOACs has prompted the EMA to launch a safety review (https://www.ema.europa.eu/documents/other/direct-oral-anticoagulants-doacs-ema-starts-review-study-bleeding-risk-direct-oral-anticoagulants_en.pdf).

In this study we determined whether differences in prescribing of DOACs compared to warfarin between General Practitioner (GP) practices across England has been associated with variation of trends in emergency hospital admissions for bleeding and thromboembolic events from GP practice registered populations.

Methods

Setting and data sources.

We identified DOACs as dabigatran etexilate, apixaban, edoxaban or rivaroxaban using the BNF codes given in Appendix 1. We used the following NHS Digital data:

1) Practice level prescribing data that are published and made available by the NHS Business Service Authority (BSA) each month. We used the Practice Prescribing Data File with the full 15-digit BNF code to calculate the annual DOAC prescribing rate as the percentage of DOAC items prescribed in proportion to all anticoagulants items prescribed for each GP practice in England between 2011 and 2016. The prescribing rate for each separate DOAC (dabigatran etexilate, apixaban, edoxaban or rivaroxaban) was calculated as the percentage of items of these medications prescribed as a proportion of all anticoagulants items prescribed. A prescription item refers to a single item prescribed on a prescription form, generally a course of medicine and is routinely used to measure trends in prescribing. We used the Net Ingredient Costs of these items included in this data set to calculate the prescribing costs.

2) Hospital Episode Statistics data were used to calculate the annual emergency admission rate for bleeding and clotting conditions for each GP practice. The ICD10 diagnostic codes included in this

For peer review only - http://bmjopen.bmj.com/site/about/guidelines.xhtml

indicator is given in Appendix 1. Rates per 100,000 were calculated using annual data on the number of people registered with each GP practice provided by NHS Digital.

To additionally control for trends in the population age profile and underlying trends in morbidity, we also calculated the annual proportion of a GP population that was over 75 and used annual data on the prevalence of Atrial Fibrillation (AF), Coronary Heart Disease (CHD) and Chronic Kidney Disease (CKD) for each GP practice population using data reported through the Quality and Outcome Framework (QOF) returns.

We included all GP practices in England with a registered population size of greater than 1000 that had data for all 6 years. We excluded GP practices with values for prevalence estimates from QOF that were clear outliers (more than 2 interquartile ranges (IQRs) below the first quartile or above the third quartile) as these probably represent reporting errors. In addition, we excluded practices where the practice registered population has changed by more than 20% between consecutive years as this would have reflected a large change in the practice population probable due to practice mergers or closures. This provided 5508 practices for the final analysis, each providing 6 years of data – i.e. 33,048 practice years of data. A flow chart is given in Appendix 2 detailing exclusions.

Analyses

Initially, we investigated geographical pattern of increases in DOACs prescribing across England. We mapped GP level prescribing data to Lower Super Output Areas (LSOA), based on the proportion of each GP practice's population that lived in each LSOA. Next, we plotted national maps for the DOAC prescribing rate each year. We then used a fixed effects Poisson regression model to investigate the association between the trend in prescribing within a GP practice and the trend in the rate of admissions for bleeding conditions. The outcome measure was the number of admissions and the log of the GP registered population was used as an offset. As there is potential confounding from unobserved factors that vary between GP practice populations, we used a fixed effects approach to remove these between-GP practice differences[10]. This conservative approach is the equivalent to including dummy variables

For peer review only - http://bmjopen.bmj.com/site/about/guidelines.xhtml

for each GP practice so that the model assesses the association between the trend in prescribing and the trend in hospital admissions within each GP practice. We additionally included a dummy variable for each year to account for the national trend in prescribing and emergency admissions. To additionally control for differential trends in need for anticoagulants and risk of bleeding complications in each GP population, we included annual measures of the prevalence of AF, CHD and CKD and the proportion of the GP population over 75-year olds. We used a Generalised Estimating Equation to account for the clustering of variance between GP practices[11]. To investigate whether there were different effects associated with different types of DOACs, we repeated the analysis for dabigatran, apixaban and rivaroxoban. As edoxaban was only licenced in the middle of 2015, there was insufficient data to analyse this separately. We present the effect estimates (Rate Ratios) per additional 10% of DOACs prescribed, rather than for each additional 1 percentage point increase as this reflects a more meaningful level of increase than a 1 percentage point increase. This is achieved by dividing the rate ratio by 10 to give the effect per 10 percentage points of DOAC prescribing.

Ethical approval was not required for this ecological study data analyses.

Sensitivity analysis

We subjected our analysis to a number of tests to assess the robustness of our findings. We estimated a negative binomial model as opposed to a Poisson model which is more robust to over-dispersion in the data, estimated a model using Huber-White clustered standard errors instead of a General Estimating Equations, [12] a model with an autoregressive correlation structures and replicated our models using data from all GP practices (i.e. not applying any exclusion). To test the specificity of results, we estimated our model using a gastro-intestinal infection as a Nonequivalent Dependent Variables (NDV)[13]. This outcome should *not* be influenced by a change in the exposure but could be influenced by unobserved confounding factors that influence general trends in hospitalization in a GP practice population.

Patient involvement

No patients were involved in setting the research question or the outcome measures, nor were they involved in developing plans for design or implementation of the study. Patients and patient

For peer review only - http://bmjopen.bmj.com/site/about/guidelines.xhtml

representatives within the NIHR CLAHRC NWC were asked to revise the manuscript in particular the lay language summary. The results of the study are going to be disseminated to the relevant patient and public groups through the CLAHRC NWC communication programme.

Results

Figure 1 shows the trend in the number of items and net drug costs of DOACs prescribed in England by GP practices between 2011 and 2016. Between 2011 and 2016, the number of items of anticoagulants prescribed had risen from 0.7 million to 1.4 million per month and the proportion of these that were DOACs had risen from <1% to 35%. Costs of anticoagulants rose more steeply, from £1.6 million per month in 2011 to £26 million per month by the end of 2016. This rise in costs was due to increased prescribing of DOACs, which by the end of 2016 accounted for 94% of the cost of all anticoagulant prescribing.

Figure 2 shows the geographical pattern of DOAC prescribing each year. The increase in uptake of DOACs has not been consistent across the country, with the prescribing rate increasing markedly more in some part of the country compared to others. In particular, areas around Somerset, Kent, and to a lesser extent the North East, appeared to have adopted DOACs sooner than other areas. By 2016 there was markedly lower usage of DOACs in the North West and East Anglia.

The results of the regression model indicate that differences in the trends in DOAC prescribing between GP practices from 2011 to 2016 were associated with differences in the trends in admissions for bleeding conditions. For each additional 10% of DOACs prescribed as a proportion of all anticoagulants, there was a 0.8% increase in emergency admissions for bleeding complications (RR:1.008 95% CI 1.004to 1.013). We present the effect estimates per additional 10% of DOACs prescribed as this reflects a more meaningful level of increase than a 1 percentage point increase. In other words for each additional 1% of DOACs prescribed as a proportion of all anticoagulants, there was a 0.08% increase in emergency admissions for bleeding complications (RR:1.0008 95% CI 1.0003 to 1.0013). To give the effect per 10% of DOACs prescribed presented above we have just divided the

For peer review only - http://bmjopen.bmj.com/site/about/guidelines.xhtml

results for 1 percent change by 10. Increased risk of emergency admissions for bleeding complications was particularly associated with increased use of dabigatran. For each additional 10% of DOACs prescribed as a proportion of all anticoagulants, there was a 0.5% reduction in emergency admissions for clotting complications (RR:0.995 95% CI 0.99 to 1.0004, p=0.08 see Figure 3. The confidence intervals suggest that this result could have occurred even if there was no true association between DOAC prescribing and emergency admissions for clotting complications.

Figure 4 shows the trend in emergency admissions for bleeding conditions between 2011 and 2016 and the trend, estimated from the regression model that would have been expected if DOACs had not been introduced. Overall the introduction of DOACs between 2011 and 2016 was associated with 1.94 additional emergency admissions for bleeding complications per 100,000 people per year [0.98 to 2.9], the equivalent to a total of 4929 emergency admissions for bleeding complications (95% CI 2489 to 7370).

Our results were similar when using alternative model specifications (see Appendix 3). We found similar results when using a negative binomial model as opposed to a Poisson regression, when using Huber White standard errors or General Estimating Equations with alternative correlation structures and when replicating our model using data from all GP practices (i.e. not applying any exclusions). We found no association between the trend in DOAC prescribing and emergency admissions when applying the model with a non-equivalent dependant variable (gastro-intestinal infections).

Discussion

In this study, we demonstrated a sharp increase in the uptake of DOACs by general practitioners in the UK between 2011 and 2016, compared with the current standard, warfarin. The uptake of DOACs has not been consistent and there are geographical areas with markedly higher rate of DOAC prescribing. Worryingly, higher rates of DOAC prescribing were associated with higher rates of hospital admissions for bleeding events, with a slight decline in emergency admissions for thromboembolic complications. We projected that with every 10% increase of DOAC prescribing as a proportion of all anticoagulants,

For peer review only - http://bmjopen.bmj.com/site/about/guidelines.xhtml

there was a 0.9% increase in emergency admissions for bleeding complications. Moreover, we estimated from our regression model that between 2011 and 2016, there were an additional 4929 emergency admissions for bleeding complications, more than would have been expected if DOACs had not been introduced. At the end of 2016, 94% of the total expenditure on anticoagulants prescribed by GP practices in England was spent on DOACs, which represented only 35% of all prescribed anticoagulants.

The strengths and limitations of the study

The strength of this study is that we analysed data over several years that included the changes in the guidelines for prescribing anticoagulants. In 2014, the UK National Institute for Health and Care Excellence (NICE) guidance recommended the use of DOACs and warfarin taking into consideration clinician and patient preferences. Comparable efficacy and the evidence from RCTs of better safety of DOACs compared with warfarin, combined with no need for regular monitoring and a wider therapeutic range, resulted in a major increase in prescribing[1 3 4 6]. We captured this increase in our longitudinal analyses. Our sample includes the majority of GP practices in England and is therefore able to estimate effects across the whole population. Our model assessed the association between the trend in prescribing and the trend in hospital admissions within each GP practice and we included a dummy variable for each year to account for the national trends. Any bias in our model of the association between our main exposure variable and outcome is therefore unlikely to be the result of time-invariant confounders that differ between GP practice population, or from any changes over time that effect all GP practices equally. We found a similar effect when using alternative models. When replicating the analysis with a common emergency admission we would not expect to be affected by DOAC prescribing such as gastrointestinal infections, we found, as expected, no association. Additional strength of our study is that we included in our analyses both, effectiveness and safety data on anticoagulants used in England. We investigated whether increased bleeding risk has been compensated by a reduction in risk from clotting conditions and found that although estimates of Rate Ratios were less than 1, confidence intervals crossed 1, indicating that this result could have occurred even if there was no true association between DOAC prescribing and emergency admissions for clotting complications.

For peer review only - http://bmjopen.bmj.com/site/about/guidelines.xhtml

A number of limitations remain. Firstly, the ecological design meant that it was not possible to link information on the prescribing for specific individuals to particular hospital admissions. Therefore, the associations observed at the aggregate level may not reflect associations at the level of the individual patients. Secondly, whilst our analysis adjusts for a number of observed and unobserved confounders, it is still possible that there are unobserved trends in factors that increase DOAC prescribing whilst also increasing risk of bleeding complications. Bias could be introduced for example if there are differential trends between GP practices in underlying risk of bleeding complications, that are not accounted for by our control variables and this increased risk of bleeding complications led to increased DOAC prescribing in these practices. As our outcomes were measured at the population level our study will, however, be less at risk of selection bias, than studies using individual follow up, where this results from clinical decisions to preferentially prescribe DOACs to patients with higher risk of bleeding complications. Thirdly, we could not control for adherence to medications in our study. Adherence to anticoagulants has been reviewed recently[14] with some contradictory reports. Although it is expected that adherence would be better for DOACs because of no need for regular monitoring, regular INR tests required to ensure the correct dose of warfarin lead to stringent check-ups for adherence, while DOACs monitoring is not recommended and therefore adherence declines after the initial period[15]. With long term adherence monitoring our estimates would have been more precise.

Comparisons with previous studies

Our results differ from the findings of several large-scale non-inferiority RCTs that have demonstrated better bleeding profiles of DOACs over warfarin[5 16-21]. This may not be surprising as trial data may provide limited information on relatively rare adverse effects due to low power and short follow up. In addition, participants in clinical trials may be younger with a fewer comorbidities. For example, renal impairment that occurs in the older population may be relevant. Renal clearance is more dominant for DOACs compared with warfarin, as 80%, 50%, 36% and 27% of unchanged dabigatran, edoxaban, rivaroxaban and apixaban respectively, are excreted in urine[22]. In addition, there is limited information available on the risk-benefit profile of DOACs in patients with severe renal function impairment[23-26]

For peer review only - http://bmjopen.bmj.com/site/about/guidelines.xhtml

particularly in patients with diverse ethnic backgrounds[27]. Recently, high inter-individual variability in DOAC plasma levels was observed in clinical practice [28].

Our findings also differ from the results of a recent retrospective cohort study in patients with intracranial haemorrhage who were previously prescribed warfarin or DOACs. Prior use of DOACs compared with prior use of warfarin was associated with lower risk of hospital mortality[29]. A recent network meta-analysis by Lopez-Lopez also showed that DOACs are safer than warfarin in relation to major and intracranial bleeding[6]. However interestingly, in that meta-analysis the risk of gastrointestinal bleeding was higher with dabigatran, edoxaban and rivaroxaban than with warfarin. In addition, edoxaban (30mg and 60 mg twice daily) significantly increased the risk of clinically relevant bleeding compared with warfarin[6]. Although our study uses a longitudinal ecological study design, at least in part, our results are comparable with previous studies. In a large (>59,000 participants) population-based observational study conducted in Canada and the US, the risk of hospital admissions for major bleeding or all-cause mortality in the first 90 days of treatment was similar for DOACs and warfarin [30]. Furthermore, in a study that used an US commercial database of 38 million people warfarin users were hospitalised longer, stayed longer in an intensive care units than dabigatran or rivaroxaban users, but there was no difference in 30- or 90-day all-cause mortality[31].

It has been estimated that the cost of DOACs will rise sharply before patent expiry (in 2022) and by the year 2020 will constitute approximately 5% of the total NHS drug budget[15]. The drugs budget is the NHS's second biggest cost after its staff[32]. Cost of warfarin including INR monitoring for one patient per annum has been estimated to be £220 (https://www.gwh.nhs.uk/media/236108/doacs-for-dvt-pe-august-2016-v-9.pdf), while the cost of rivaroxaban (15mg bd for 3/52 loading (provided by hospital), then 20mg o.d. has been estimated to £657 p.a. (https://www.gwh.nhs.uk/media/236108/doacs-for-dvt-pe-august-2016-v-9.pdf). Similar costs have been estimated for other DOACs. Additionally, there is a huge discrepancy in price of agents that are used to reverse the anticoagulant effects of warfarin (antidote vitamin K at £0.38) or DOACs (idarucizumab, reversal agent for dabigatran at £2,400 for a single treatment course of 2X 2.5 g infusions (https://www.nice.org.uk/advice/esnm73) or andexanet

For peer review only - http://bmjopen.bmj.com/site/about/guidelines.xhtml

alpha, reversal agent for factor Xa inhibitors approved by the FDA in May 2018, at approximately £1,500). These high costs of DOACs could be avoided if warfarin treatment is optimised, for example through novel methods such as genotype-guided dosing[33 34] and point-of-care INR monitoring[35], and only individuals who are at higher risk of developing bleeding events, for example those with variant alleles that increase the risk of bleeding from warfarin[36], could be prescribed DOACs. Genotype-guided dosing of warfarin has been shown to be cost-effective[37]. It has been shown previously that approximately 55% of variability in warfarin dose requirements can be estimated from clinical and genetic data of three polymorphisms in the warfarin molecular target (vitamin K epoxide reductase gene) and metabolising enzyme (CYP2C9 gene) and genotype-guided dosing can reduce the risk of major bleeding[38 39].

Conclusions

This study is one of a few studies in the UK[1 40 41] that have evaluated the association between the rise in prescribing of DOACs after changes in NICE guidance in 2014 and emergency hospital admissions for bleeding events. We found that the rate of emergency admissions for bleeding conditions increased to a greater extent in GP practices that were more likely to prescribe DOACs compared with warfarin. With rapidly increasing use of DOACs, these potential adverse consequences need to be taken into account when assessing the benefits and costs of anticoagulant treatment in clinical practice. It is not clear whether the DOACs are being prescribed without adequate notice being taken of restrictions and warnings in the summary of product characteristics, and in addition, whether closer monitoring is required in certain patient groups to increase the safety of use in clinical practice. The recently announced review by the EMA is thus important in order to further optimise the use of all oral anticoagulants so that risks are minimised while at the same time maximising benefits.

PPI statement

For peer review only - http://bmjopen.bmj.com/site/about/guidelines.xhtml

The study was supported by a patient advisory group, which provided input to the NIHR- CLAHRC NWC programme of research. This patient advisory group met on a regular basis for the duration of the study. Due to data protection restrictions and the very technical methods required to do our data analyses they were not involved in the analysis part of the project. PPI representatives will however, write a plain language summary and design a leaflet for dissemination to their peers and distributing to patient groups.

Author contributions

Ana Alfirevic- Substantial contributions to the conception and design of the work, drafting a manuscript. Agreed to be accountable for all aspects of the work in ensuring that questions related to the accuracy or integrity of any part of the work are appropriately investigated and resolved.

Ben Barr- Substantial contribution to the design of the work, the acquisition, analysis and interpretation of data. Agreed to be accountable for all aspects of the work in ensuring that questions related to the accuracy or integrity of any part of the work are appropriately investigated and resolved.

Munir Pirmohamed- Substantial contribution to the interpretation of the results, critical revision of the manuscript for important intellectual content and final approval of the manuscript.

Jennifer Downing- Critical revision of the manuscript for important intellectual content and final approval of the manuscript.

Konstantinos Daras- Substantial contribution to data acquisition, data analysis and interpretation of data for the work. Critical revision of the manuscript for important intellectual content and final approval of the manuscript

Terence Comerford- Critical revision of the manuscript for important intellectual content and final approval of the manuscript. Preparation of plain language summary for dissemination of findings.

Patient consent for publication: *Not required.*
Funding: *This work was supported by the National Institute for Health Research, Collaboration for Leadership and Health Research and Care North West Coast (NIHR CLAHRC NWC) and the state Department of Health and Social Care.*
Disclaimer: *The views expressed are those of the author(s) and not necessarily those of the NHS, the NIHR or the Department of Health.*
Competing interests: *We have read and understood BMJ policy on declaration of interests and declare the following: All authors have completed the ICMJE uniform disclosure form at http://www.icmje.org/coi_disclosure.pdf and declare: no support from any organisation for the submitted work [or describe if any]; no financial relationships with any organisations that might have an interest in the submitted work in the previous three years [or describe if any], no other relationships or activities that could appear to have influenced the submitted work [or describe if any].*

Provenance and peer review: *Not commissioned; externally peer reviewed.*
Data sharing statement: *The dataset from this study will be made available on request to benbarr@liverpool.ac.uk*

For peer review only - http://bmjopen.bmj.com/site/about/guidelines.xhtml

References

1. Vinogradova Y, Coupland C, Hill T, et al. Risks and benefits of direct oral anticoagulants versus warfarin in a real world setting: cohort study in primary care. *BMJ (Clinical research ed* 2018;362:k2505. doi: 10.1136/bmj.k2505
2. Agnelli G, Buller HR, Cohen A, et al. Oral apixaban for the treatment of acute venous thromboembolism. *The New England journal of medicine* 2013;369(9):799-808. doi: 10.1056/NEJMoa1302507
3. Investigators E, Bauersachs R, Berkowitz SD, et al. Oral rivaroxaban for symptomatic venous thromboembolism. *The New England journal of medicine* 2010;363(26):2499-510. doi: 10.1056/NEJMoa1007903
4. Investigators E-P, Buller HR, Prins MH, et al. Oral rivaroxaban for the treatment of symptomatic pulmonary embolism. *The New England journal of medicine* 2012;366(14):1287-97. doi: 10.1056/NEJMoa1113572
5. Schulman S, Kearon C, Kakkar AK, et al. Dabigatran versus warfarin in the treatment of acute venous thromboembolism. *The New England journal of medicine* 2009;361(24):2342-52. doi: 10.1056/NEJMoa0906598
6. Lopez-Lopez JA, Sterne JAC, Thom HHZ, et al. Oral anticoagulants for prevention of stroke in atrial fibrillation: systematic review, network meta-analysis, and cost effectiveness analysis. *BMJ (Clinical research ed* 2017;359:j5058. doi: 10.1136/bmj.j5058
7. Protty MB, Hayes J. Dawn of the direct-acting oral anticoagulants: trends in oral anticoagulant prescribing in Wales 2009-2015. *Journal of clinical pharmacy and therapeutics* 2017;42(2):132-34. doi: 10.1111/jcpt.12481
8. Burnett A, Siegal D, Crowther M. Specific antidotes for bleeding associated with direct oral anticoagulants. *BMJ (Clinical research ed* 2017;357:j2216. doi: 10.1136/bmj.j2216
9. Mueller T, Alvarez-Madrazo S, Robertson C, et al. Comparative safety and effectiveness of direct oral anticoagulants in patients with atrial fibrillation in clinical practice in Scotland. *British journal of clinical pharmacology* 2019;85(2):422-31. doi: 10.1111/bcp.13814
10. Gunasekara FI, Richardson K, Carter K, et al. Fixed effects analysis of repeated measures data. *International journal of epidemiology* 2014;43(1):264-9. doi: 10.1093/ije/dyt221 [published Online First: 2013/12/25]
11. Harding JW, Hilbe JM. Generalized Estimating Equations. 2 edition ed. Boca Raton, FL: Chapman and Hall/CRC 2012.
12. Rogers W. Regression standard errors in clustered samples. *Stata Technical Bulletin* 1994;3
13. Shadish WR, Cook TD, Thomas. CD. Experimental and Quasi-Experimental Designs for Generalised Causal Inference: Houghton Mifflin 2002.
14. Obamiro KO, Chalmers L, Bereznicki LR. A Summary of the Literature Evaluating Adherence and Persistence with Oral Anticoagulants in Atrial Fibrillation. *American journal of cardiovascular drugs : drugs, devices, and other interventions* 2016;16(5):349-63. doi: 10.1007/s40256-016-0171-6
15. Burn J, Pirmohamed M. Direct oral anticoagulants versus warfarin: is new always better than the old? *Open heart* 2018;5(1):e000712. doi: 10.1136/openhrt-2017-000712
16. Connolly SJ, Ezekowitz MD, Yusuf S, et al. Dabigatran versus warfarin in patients with atrial fibrillation. *The New England journal of medicine* 2009;361(12):1139-51. doi: 10.1056/NEJMoa0905561

For peer review only - http://bmjopen.bmj.com/site/about/guidelines.xhtml

17. Ezekowitz MD, Connolly S, Parekh A, et al. Rationale and design of RE-LY: randomized evaluation of long-term anticoagulant therapy, warfarin, compared with dabigatran. *American heart journal* 2009;157(5):805-10, 10 e1-2. doi: 10.1016/j.ahj.2009.02.005
18. Ogawa S, Shinohara Y, Kanmuri K. Safety and efficacy of the oral direct factor xa inhibitor apixaban in Japanese patients with non-valvular atrial fibrillation. -The ARISTOTLE-J study. *Circulation journal : official journal of the Japanese Circulation Society* 2011;75(8):1852-9.
19. Patel MR, Mahaffey KW, Garg J, et al. Rivaroxaban versus warfarin in nonvalvular atrial fibrillation. *The New England journal of medicine* 2011;365(10):883-91. doi: 10.1056/NEJMoa1009638
20. Ruff CT, Giugliano RP, Antman EM, et al. Evaluation of the novel factor Xa inhibitor edoxaban compared with warfarin in patients with atrial fibrillation: design and rationale for the Effective aNticoaGulation with factor xA next GEneration in Atrial Fibrillation-Thrombolysis In Myocardial Infarction study 48 (ENGAGE AF-TIMI 48). *American heart journal* 2010;160(4):635-41. doi: 10.1016/j.ahj.2010.06.042
21. Weitz JI, Connolly SJ, Patel I, et al. Randomised, parallel-group, multicentre, multinational phase 2 study comparing edoxaban, an oral factor Xa inhibitor, with warfarin for stroke prevention in patients with atrial fibrillation. *Thromb Haemost* 2010;104(3):633-41. doi: 10.1160/TH10-01-0066
22. Lutz J, Jurk K, Schinzel H. Direct oral anticoagulants in patients with chronic kidney disease: patient selection and special considerations. *International journal of nephrology and renovascular disease* 2017;10:135-43. doi: 10.2147/IJNRD.S105771
23. Buckley LF, Rybak E, Aldemerdash A, et al. Direct oral anticoagulants in patients with atrial fibrillation and renal impairment, extremes in weight, or advanced age. *Clinical cardiology* 2017;40(1):46-52. doi: 10.1002/clc.22591
24. Fanikos J, Burnett AE, Mahan CE, et al. Renal Function and Direct Oral Anticoagulant Treatment for Venous Thromboembolism. *The American journal of medicine* 2017;130(10):1137-43. doi: 10.1016/j.amjmed.2017.06.004
25. Fanikos J, Burnett AE, Mahan CE, et al. Renal Function Considerations for Stroke Prevention in Atrial Fibrillation. *The American journal of medicine* 2017;130(9):1015-23. doi: 10.1016/j.amjmed.2017.04.015
26. Zou R, Tao J, Shi W, et al. Meta-analysis of safety and efficacy for direct oral anticoagulation treatment of non-valvular atrial fibrillation in relation to renal function. *Thrombosis research* 2017;160:41-50. doi: 10.1016/j.thromres.2017.10.013
27. Wang KL, Chiu CC, Giugliano RP, et al. Drug Class, Renal Elimination, and Outcomes of Direct Oral Anticoagulants in Asian Patients: A Meta-Analysis. *J Stroke Cerebrovasc Dis* 2018;27(4):857-64. doi: 10.1016/j.jstrokecerebrovasdis.2017.10.027
28. Testa S, Tripodi A, Legnani C, et al. Plasma levels of direct oral anticoagulants in real life patients with atrial fibrillation: Results observed in four anticoagulation clinics. *Thrombosis research* 2016;137:178-83. doi: 10.1016/j.thromres.2015.12.001 [published Online First: 2015/12/18]
29. Inohara T, Xian Y, Liang L, et al. Association of Intracerebral Hemorrhage Among Patients Taking Non-Vitamin K Antagonist vs Vitamin K Antagonist Oral Anticoagulants With In-Hospital Mortality. *JAMA* 2018;319(5):463-73. doi: 10.1001/jama.2017.21917
30. Jun M, Lix LM, Durand M, et al. Comparative safety of direct oral anticoagulants and warfarin in venous thromboembolism: multicentre, population based, observational study. *BMJ (Clinical research ed* 2017;359:j4323. doi: 10.1136/bmj.j4323
31. Charlton B, Adeboyeje G, Barron JJ, et al. Length of hospitalization and mortality for bleeding during treatment with warfarin, dabigatran, or rivaroxaban. *PLoS One* 2018;13(3):e0193912. doi: 10.1371/journal.pone.0193912

For peer review only - http://bmjopen.bmj.com/site/about/guidelines.xhtml

32. The L. Personalised medicine in the UK. *Lancet* 2018;391(10115):e1. doi: 10.1016/S0140-6736(17)33261-0
33. Yang T, Zhou Y, Chen C, et al. Genotype-guided dosing versus conventional dosing of warfarin: A meta-analysis of 15 randomized controlled trials. *Journal of clinical pharmacy and therapeutics* 2018 doi: 10.1111/jcpt.12782
34. Jorgensen AL, Prince C, Fitzgerald G, et al. Implementation of genotype-guided dosing of warfarin with point-of-care genetic testing in three UK clinics: a matched cohort study. *BMC Med* 2019;17(1):76. doi: 10.1186/s12916-019-1308-7
35. Wool GD. Benefits and Pitfalls of Point-of-Care Coagulation Testing for Anticoagulation Management: An ACLPS Critical Review. *American journal of clinical pathology* 2019;151(1):1-17. doi: 10.1093/ajcp/aqy087
36. Kawai VK, Cunningham A, Vear SI, et al. Genotype and risk of major bleeding during warfarin treatment. *Pharmacogenomics* 2014;15(16):1973-83. doi: 10.2217/pgs.14.153
37. Pink J, Pirmohamed M, Lane S, et al. Cost-effectiveness of pharmacogenetics-guided warfarin therapy vs. alternative anticoagulation in atrial fibrillation. *Clin Pharmacol Ther* 2014;95(2):199-207. doi: 10.1038/clpt.2013.190
38. Gage BF, Bass AR, Lin H, et al. Effect of Genotype-Guided Warfarin Dosing on Clinical Events and Anticoagulation Control Among Patients Undergoing Hip or Knee Arthroplasty: The GIFT Randomized Clinical Trial. *JAMA* 2017;318(12):1115-24. doi: 10.1001/jama.2017.11469
39. Pirmohamed M, Burnside G, Eriksson N, et al. A randomized trial of genotype-guided dosing of warfarin. *The New England journal of medicine* 2013;369(24):2294-303. doi: 10.1056/NEJMoa1311386
40. Green L, Tan J, Antoniou S, et al. Haematological management of major bleeding associated with direct oral anticoagulants - UK experience. *Br J Haematol* 2019 doi: 10.1111/bjh.15808
41. Green L, Tan J, Morris JK, et al. A three-year prospective study of the presentation and clinical outcomes of major bleeding episodes associated with oral anticoagulant use in the UK (ORANGE study). *Haematologica* 2018;103(4):738-45. doi: 10.3324/haematol.2017.182220

For peer review only

For peer review only - http://bmjopen.bmj.com/site/about/guidelines.xhtml

Figure legends

Figure 1. Trend in anticoagulant prescribing items and costs from GP practices in England and the proportion of direct oral anticoagulants of all anticoagulants prescribed. In 2014, NICE guidance on use of direct oral anticoagulants has changed.

Figure 2. Geographical pattern in DOAC prescribing as a proportion of all anticoagulant prescribing items in England 2011-2016 that includes changes in NICE guidance in 2014.

Figure 3 Estimates from regression model showing the relative change in the rate of emergency admissions for bleeding and clotting complications associated with each additional 10% of DOACs prescribed as a proportion of all anticoagulants.

Figure 4. Trend in emergency admissions for bleeding conditions between 2011 and 2016 and the trend, estimated from the regression model that would have been expected if DOACs had not been introduced.

For peer review only

For peer review only - http://bmjopen.bmj.com/site/about/guidelines.xhtml

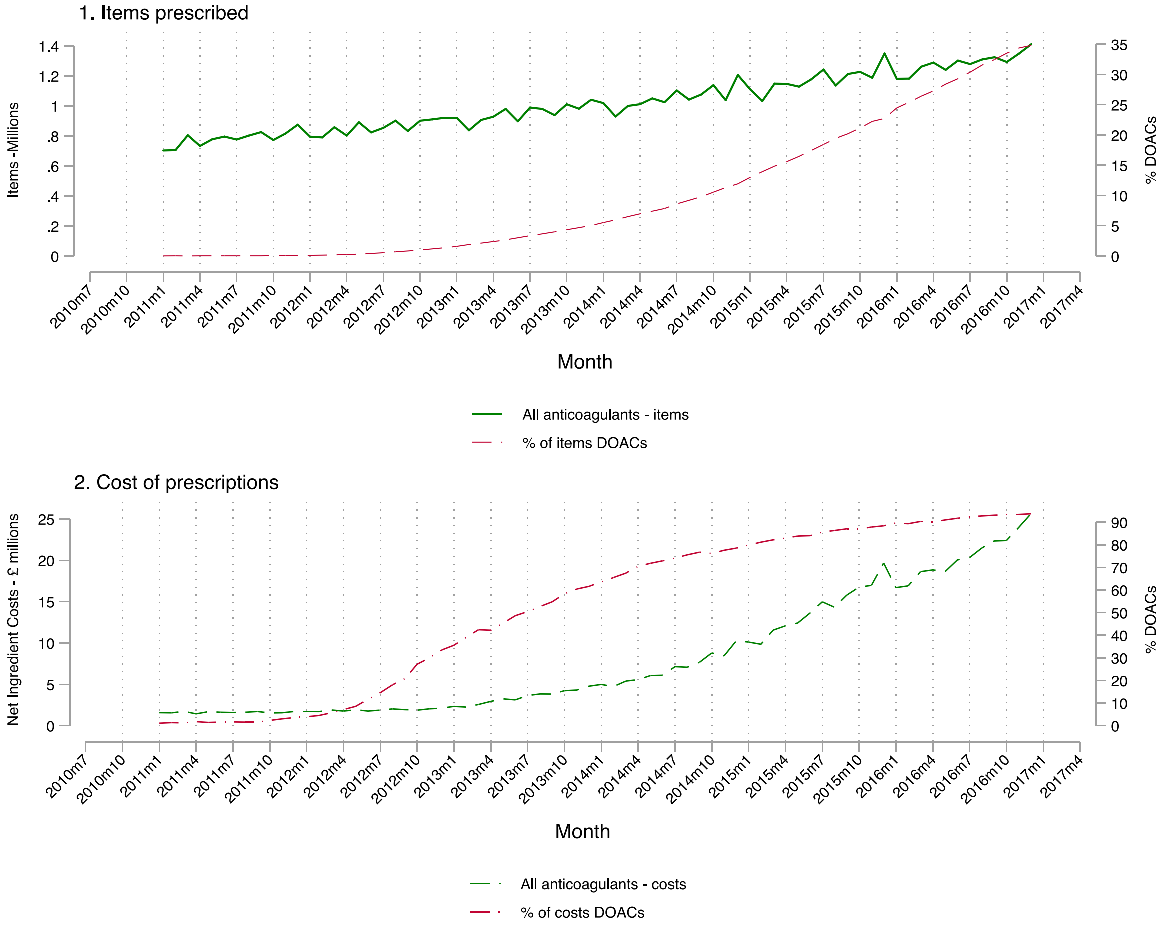
1. Items prescribed
Items -Millions
% DOACs
Month
All anticoagulants - items
% of items DOACs
2. Cost of prescriptions
Net Ingredient Costs - £ millions
% DOACs
Month
All anticoagulants - costs
% of costs DOACs

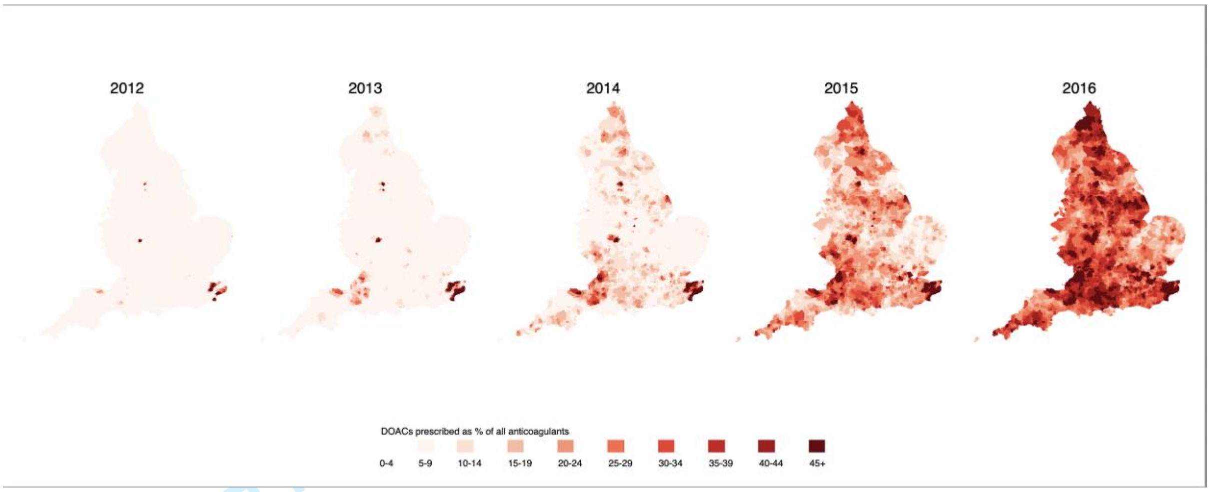

For peer review only

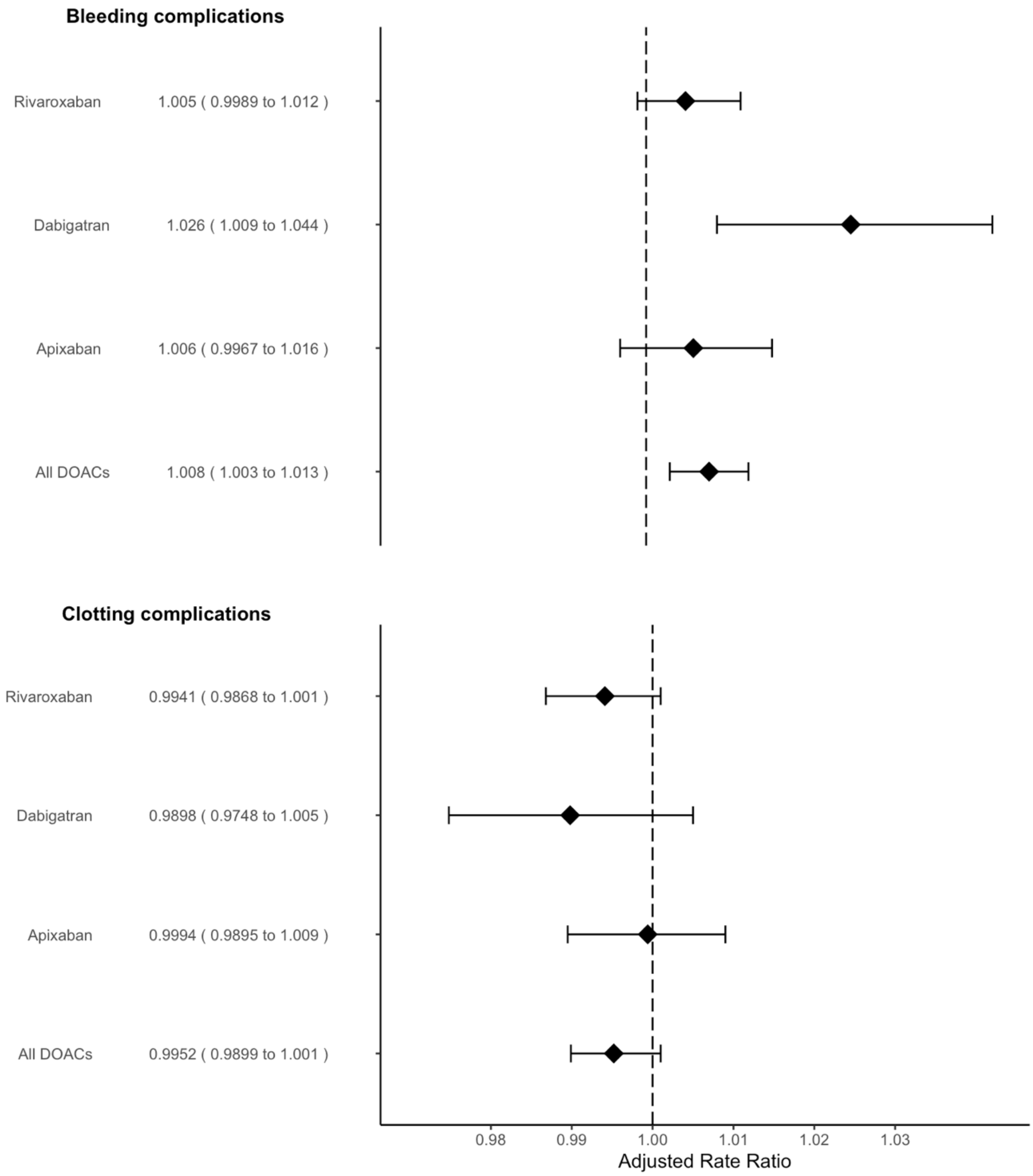
Bleeding complications
Rivaroxaban 1.005 (0.9989 to 1.012)
Dabigatran 1.026 (1.009 to 1.044)
Apixaban 1.006 (0.9967 to 1.016)
All DOACs 1.008 (1.003 to 1.013)
Clotting complications
Rivaroxaban 0.9941 (0.9868 to 1.001)
Dabigatran 0.9898 (0.9748 to 1.005)
Apixaban 0.9994 (0.9895 to 1.009)
All DOACs 0.9952 (0.9899 to 1.001)
0.98
0.99
1.00
1.01
1.02
1.03
Adjusted Rate Ratio

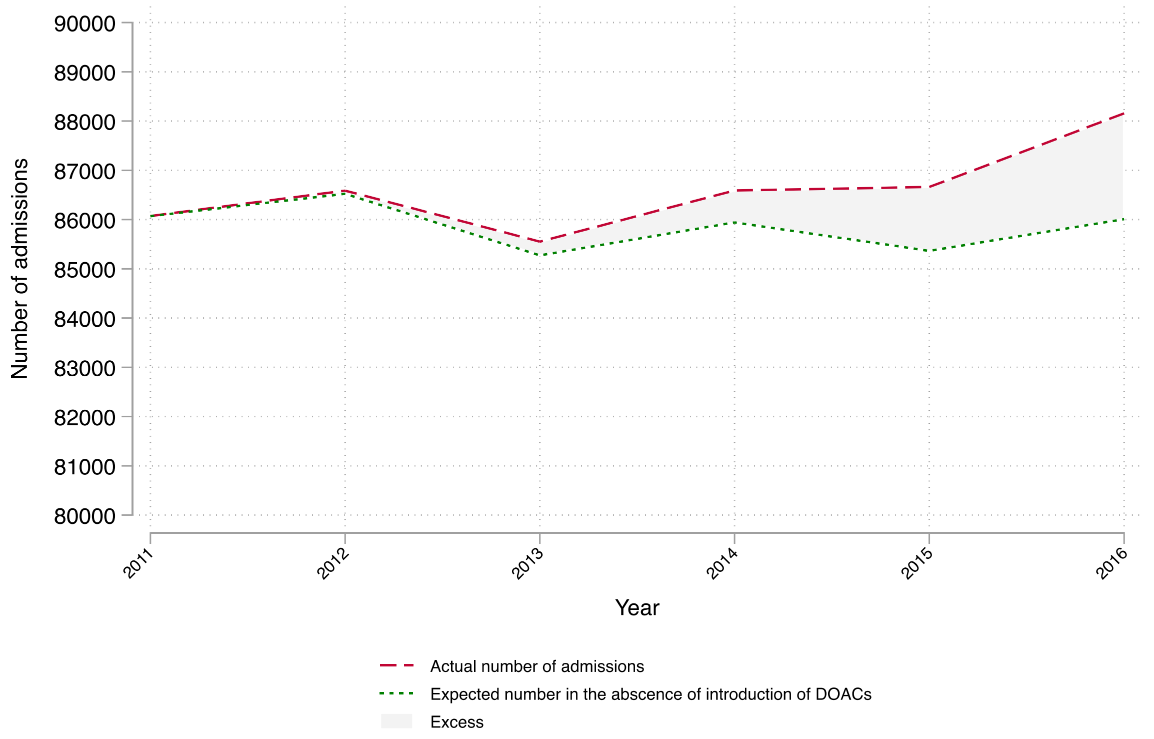

For peer review only

Appendix 1 BNF codes and ICD10 codes used to define exposure and outcome variables.

BNF codes used to define prescribing

0208020V - Warfarin

0208020X0 – Dabigatran

0208020X0 – edoxaban

0208020Y – rivaroxaban

0208020Z0 - Apixiban

ICD 10 Codes used to define outcomes

1. **Bleeding Complications**

ICD10 code	Name
M79.81	traumatic haematoma of the soft tissue
M25.00	Hemarthrosis, unspecified joint
K92	Other diseases of digestive system
K92.0	Haematemesis
K92.1	Melaena
K92.2	Gastrointestinal haemorrhage, unspecified
K62.5	Haemorrhage of anus and rectum
R58	Haemorrhage, not elsewhere classified
R04.0	Epistaxis
R04.1	Haemorrhage from throat
R04.2	Haemoptysis
R04.8	Haemorrhage from other sites in respiratory passages

For peer review only

R04.9	Haemorrhage from respiratory passages, unspecified
R31	Unspecified haematuria
N02	Recurrent or persistent haematuria
I60	Subarachnoid haemorrhage
I60.0	Subarachnoid haemorrhage from carotid siphon and bifurcation
I60.1	Subarachnoid haemorrhage from middle cerebral artery
I60.2	Subarachnoid haemorrhage from anterior communicating artery
I60.3	Subarachnoid haemorrhage from posterior communicating artery
I60.4	Subarachnoid haemorrhage from basilar artery
I60.5	Subarachnoid haemorrhage from vertebral artery
I60.6	Subarachnoid haemorrhage from other intracranial arteries
I60.7	Subarachnoid haemorrhage from intracranial artery, unspecified
I60.8	Other subarachnoid haemorrhage
I60.9	Subarachnoid haemorrhage, unspecified
I61	Intracerebral haemorrhage
I61.0	Intracerebral haemorrhage in hemisphere, subcortical
I61.1	Intracerebral haemorrhage in hemisphere, cortical
I61.2	Intracerebral haemorrhage in hemisphere, unspecified
I61.3	Intracerebral haemorrhage in brain stem
I61.4	Intracerebral haemorrhage in cerebellum
I61.5	Intracerebral haemorrhage, intraventricular
I61.6	Intracerebral haemorrhage, multiple localized
I61.8	Other intracerebral haemorrhage
I61.9	Intracerebral haemorrhage, unspecified
I62	Other nontraumatic intracranial haemorrhage
I62.0	Subdural haemorrhage (acute)(nontraumatic)
I62.1	Nontraumatic extradural haemorrhage

For peer review only

I62.9	Intracranial haemorrhage (nontraumatic), unspecified

2. **Clotting Complications**

ICD10 code	Name
I63	Cerebral infarction
I64	Stroke, not specified as haemorrhage or infarction
I26	Pulmonary embolism
I82	Other venous embolism and thrombosis
I80.0	Phlebitis and thrombophlebitis of superficial vessels of lower extremities
I80.1	Phlebitis and thrombophlebitis of femoral vein
I80.2	Deep vein thrombosis, Phlebitis and thrombophlebitis of other deep vessels of lower extremities
I80.3	Phlebitis and thrombophlebitis of lower extremities, unspecified
I80.8	Phlebitis and thrombophlebitis of other sites
I80.9	Phlebitis and thrombophlebitis of unspecified site

For peer review only

Appendix 2 flow outlining inclusion/ exclusion and missing data

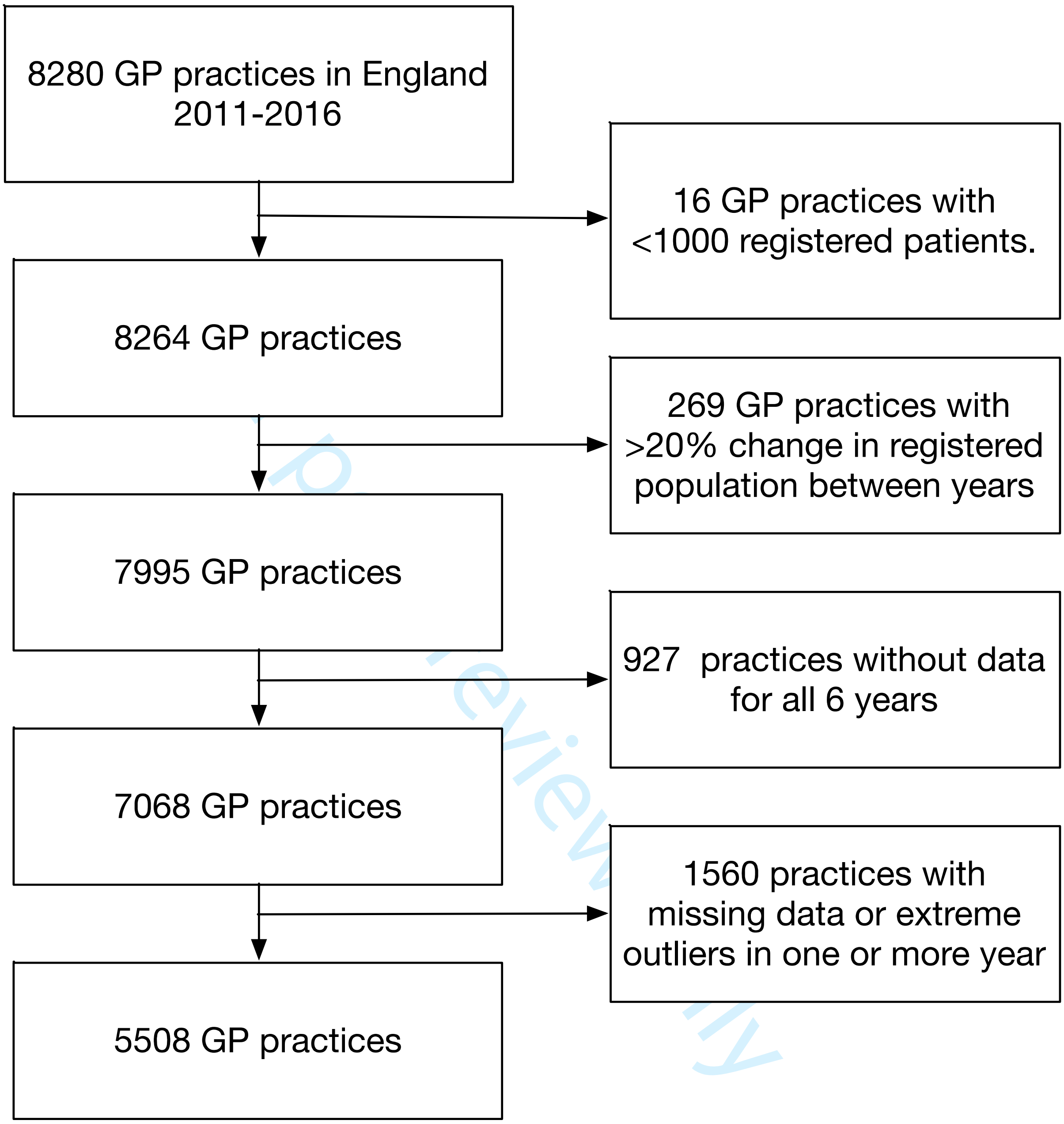

Appendix 3 alternative model specifications.

1. **Model using all GP practices – without applying exclusion criteria (8032 GP practices – 248 with missing data). Estimates from regression model showing the relative change in the rate of emergency admissions for bleeding and clotting complications associated with each additional 10% of DOACs prescribed as a proportion of all anticoagulants**

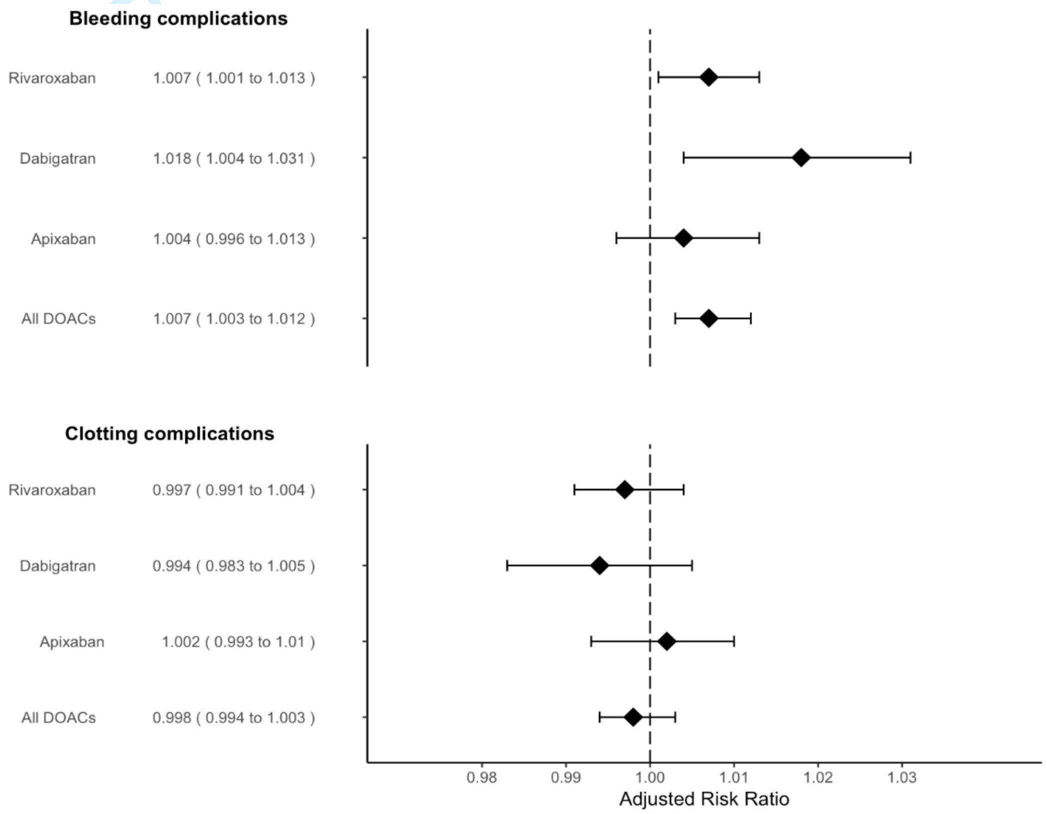

2. **Negative Binomial Model**

Estimates from regression model showing the relative change in the rate of emergency admissions for bleeding and clotting complications associated with each additional 10% of DOACs prescribed as a proportion of all anticoagulants

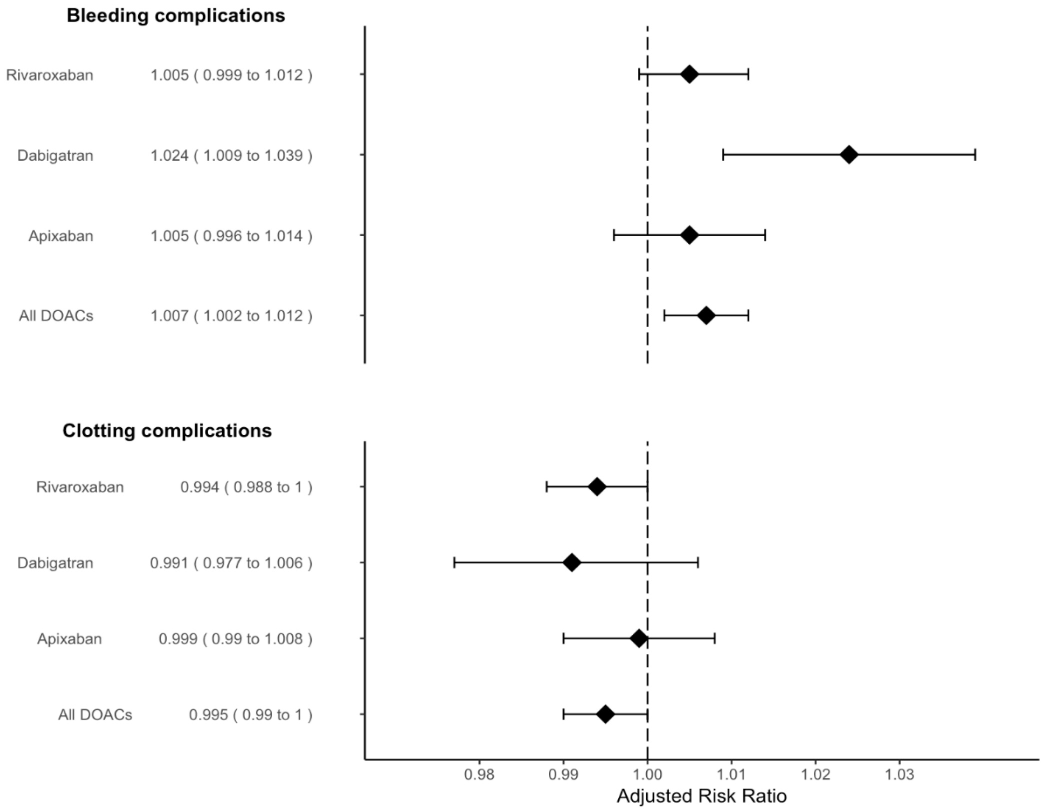

3. Non-equivalent dependent variable.

Estimates from regression model showing the relative change in the rate of emergency admissions for gastrointestinal admissions associated with each additional 10% of DOACs prescribed as a proportion of all anticoagulants.

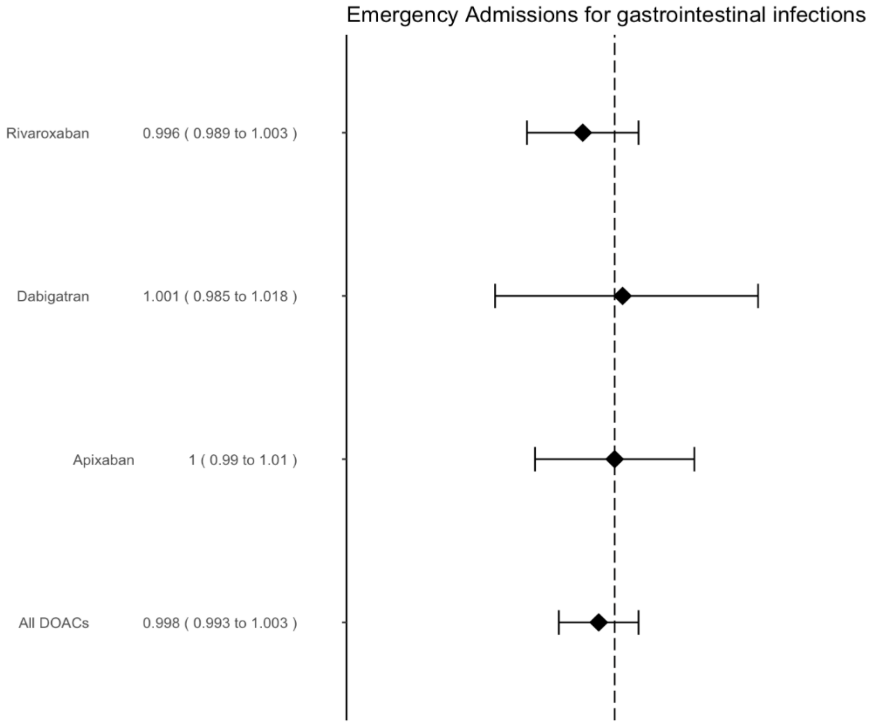

w only

4. **Huber White Standard Errors**

Estimates from regression model showing the relative change in the rate of emergency admissions for bleeding and clotting complications associated with each additional 10% of DOACs prescribed as a proportion of all anticoagulants

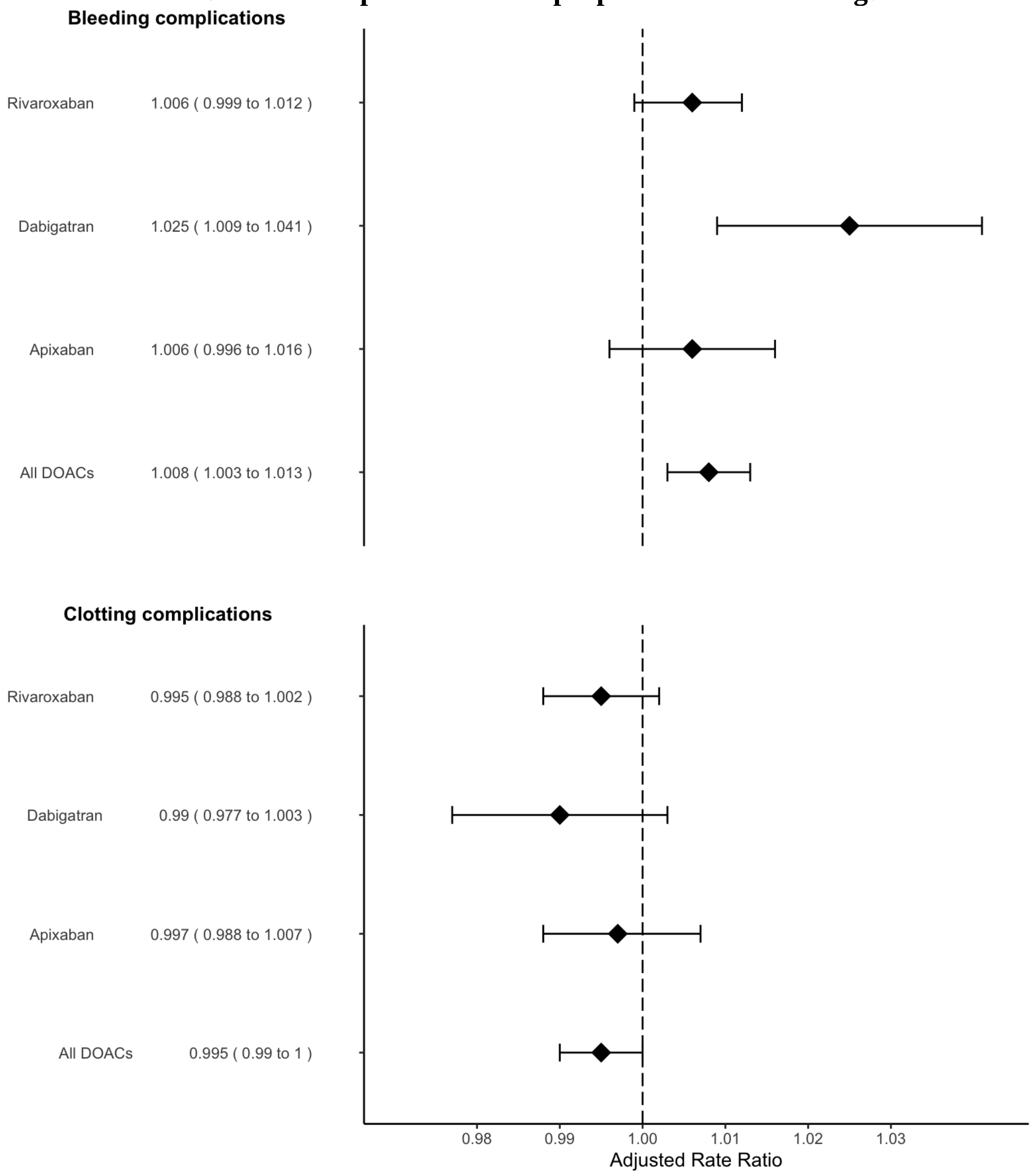

5. General Estimating equation - autoregressive(AR-1) correlation structure. Estimates from regression model showing the relative change in the rate of emergency admissions for bleeding and clotting complications associated with each additional 10% of DOACs prescribed as a proportion of all anticoagulants

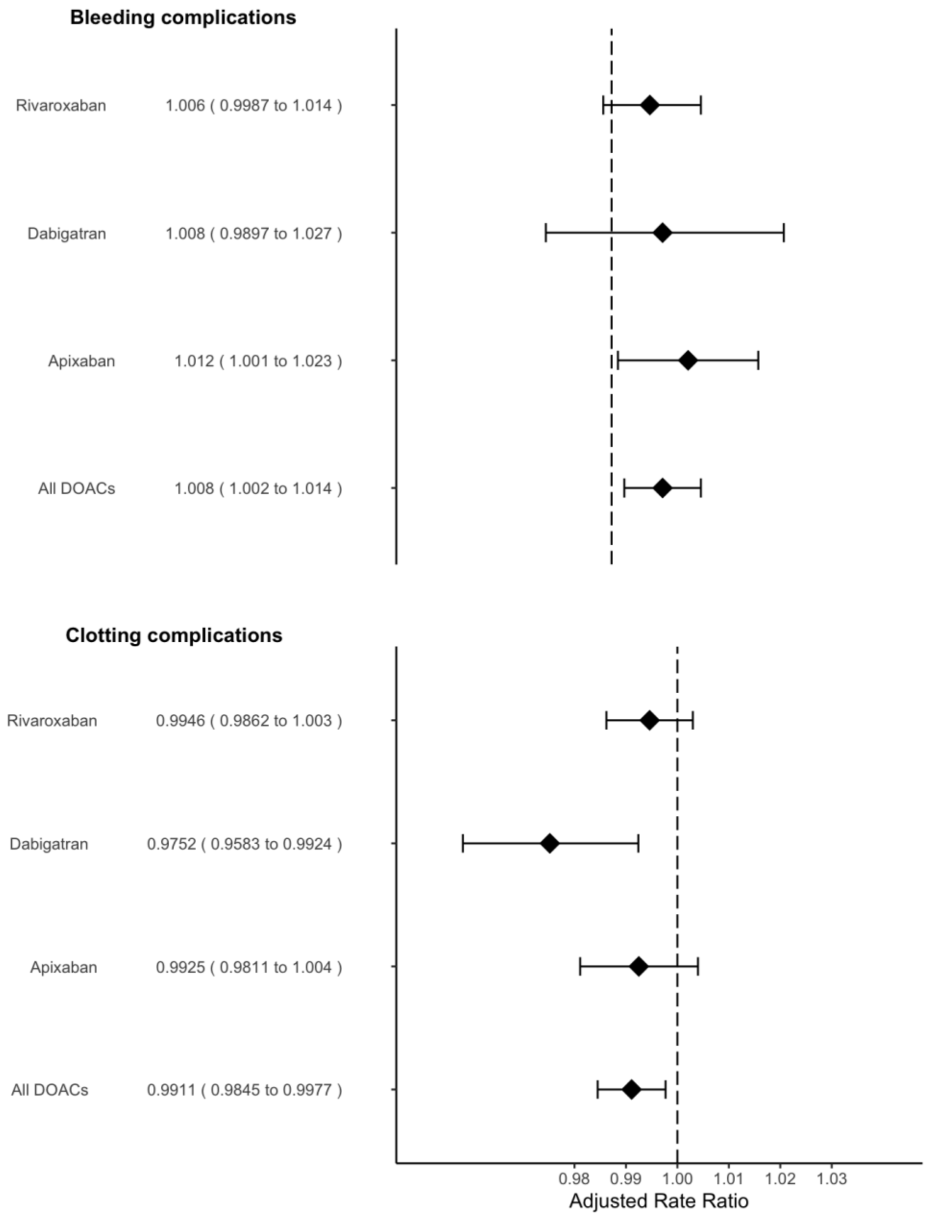

STROBE Statement—checklist of items that should be included in reports of observational studies
Paper submission:
Alfirevic et al. Has the introduction of direct oral anticoagulants (DOACs) in England increased emergency admissions for bleeding conditions? A longitudinal ecological study.

	Item No	Recommendation
**Title and abstract**	1	(*a*) We've indicated the study's design in the title or the abstract
		(*b*) We've provided in the abstract an informative and balanced summary of what was done and what was found, page 2
**Introduction**		
Background/rationale	2	We've explained the scientific background and rationale for the investigation being reported, pages 3 and 4
Objectives	3	State specific objectives, including any prespecified hypotheses- done, page 4, line 12 to 18
**Methods**		
Study design	4	Key elements of study design were presented on page 5, lines 12 to 27
Setting	5	The setting, locations, and relevant dates, including periods of recruitment, exposure, follow-up, and data collection were presented on pages 4 and 5
Participants	6	We included all GP practices in England with a registered population size greater than 1000, which had data for all 6 years (N=5508 GP practices with 33,048 practice years of data. Page 5
		Inclusions/exclusions and missing data are outlined in Supplementary material- Appendix 2
Variables	7	Clearly defined outcomes (BNF and ICD10 codes) are presented in Supplementary material, Appendix 1
Data sources/ measurement	8*	Sources of data and details of methods of assessment are presented on pages 4 and 5. Describe comparability of assessment methods if there is more than one group
Bias	9	Potential sources of bias such as confounding from unobserved factors that vary between GP practice populations, we used a fixed effects approach and a dummy variable for each year to account for the national trend in prescribing and emergency admissions. In addition, we performed sensitivity analyses
Study size	10	All eligible GP practices in England were included in this study
Quantitative variables	11	We've explained how quantitative variables were handled in the analyses on pages 5 and 6
Statistical methods	12	(*a*) Describe all statistical methods, including those used to control for confounding- done, page 5 and 6
		(*b*) Describe any methods used to examine subgroups and interactions, done page 5 and 6
		(*c*) Explain how missing data were addressed- done flow chart Supplementary material Appendix 2
		(*e*) Sensitivity analyses are described on page 6

Continued on next page

For peer review only - http://bmjopen.bmj.com/site/about/guidelines.xhtml

**Results**		
Participants	13*	(a) Report numbers of individuals at each stage of study-done, Supplementary material Appendix 2
		(b) Give reasons for non-participation at each stage- done Appendix 2
		(c) Flow diagram- done and shown in Appendix 2
Descriptive data	14*	(a) Give characteristics of study participants – GP practice data included, no data on individual participants are available
		(b) We've indicated the number of practices with missing data in Appendix 2
		(c) *F*ollow-up time of 6 years has been clearly shown in the manuscript (page 5 and 7) and Figures 1 and 2
Outcome data	15*	
		Outcome data are described in the manuscript, page 7
Main results	16	(*a*) We have given unadjusted estimates and their precision (95% confidence interval), page 7
		(*c*) We haven't translated estimates of relative risk into absolute risk
Other analyses	17	We have reported sensitivity analyses, page 7, lines 50-59
**Discussion**		
Key results	18	Summarise key results with reference to study objectives- done, page 8
Limitations	19	Discuss limitations of the study, taking into account sources of potential bias or imprecision. Discuss both direction and magnitude of any potential bias- done page 9 line 22
Interpretation	20	Give a cautious overall interpretation of results considering objectives, limitations, multiplicity of analyses, results from similar studies, and other relevant evidence done page 10
Generalisability	21	Discuss the generalisability (external validity) of the study results – we have compared our results with previous studies, page 10
**Other information**		
Funding	22	Give the source of funding and the role of the funders for the present study and, if applicable, for the original study on which the present article is based- Done, we have included a sentence at the end of the manuscript to acknowledge our NIHR CLAHRC NWC funding

*Give information separately for cases and controls in case-control studies and, if applicable, for exposed and unexposed groups in cohort and cross-sectional studies.

Note: An Explanation and Elaboration article discusses each checklist item and gives methodological background and published examples of transparent reporting. The STROBE checklist is best used in conjunction with this article (freely available on the Web sites of PLoS Medicine at http://www.plosmedicine.org/, Annals of Internal Medicine at http://www.annals.org/, and Epidemiology at http://www.epidem.com/). Information on the STROBE Initiative is available at www.strobe-statement.org.

For peer review only
